# A Mechanistic, Enantioselective, Physiologically Based Pharmacokinetic Model of Verapamil and Norverapamil, Built and Evaluated for Drug–Drug Interaction Studies

**DOI:** 10.3390/pharmaceutics12060556

**Published:** 2020-06-16

**Authors:** Nina Hanke, Denise Türk, Dominik Selzer, Sabrina Wiebe, Éric Fernandez, Peter Stopfer, Valerie Nock, Thorsten Lehr

**Affiliations:** 1 Clinical Pharmacy, Saarland University, 66123 Saarbrücken, Germany; n.hanke@mx.uni-saarland.de (N.H.); denise.tuerk@uni-saarland.de (D.T.); dominik.selzer@uni-saarland.de (D.S.); 2 Translational Medicine and Clinical Pharmacology, Boehringer Ingelheim Pharma GmbH & Co. KG, 88397 Biberach, Germany; sabrina.wiebe@boehringer-ingelheim.com (S.W.); eric_gerard.fernandez@boehringer-ingelheim.com (É.F.); peter.stopfer@boehringer-ingelheim.com (P.S.); valerie.nock@boehringer-ingelheim.com (V.N.); 3 Clinical Pharmacology and Pharmacoepidemiology, Heidelberg University Hospital, 69120 Heidelberg, Germany

**Keywords:** physiologically based pharmacokinetic (PBPK) modeling, verapamil, norverapamil, drug–drug interactions (DDIs), cytochrome P450 3A4 (CYP3A4), P-glycoprotein (Pgp), mechanism-based inactivation (MBI), non-competitive inhibition, model-informed drug discovery and development (MID3)

## Abstract

The calcium channel blocker and antiarrhythmic agent verapamil is recommended by the FDA for drug–drug interaction (DDI) studies as a moderate clinical CYP3A4 index inhibitor and as a clinical Pgp inhibitor. The purpose of the presented work was to develop a mechanistic whole-body physiologically based pharmacokinetic (PBPK) model to investigate and predict DDIs with verapamil. The model was established in PK-Sim^®^, using 45 clinical studies (dosing range 0.1–250 mg), including literature as well as unpublished Boehringer Ingelheim data. The verapamil R- and S-enantiomers and their main metabolites R- and S-norverapamil are represented in the model. The processes implemented to describe the pharmacokinetics of verapamil and norverapamil include enantioselective plasma protein binding, enantioselective metabolism by CYP3A4, non-stereospecific Pgp transport, and passive glomerular filtration. To describe the auto-inhibitory and DDI potential, mechanism-based inactivation of CYP3A4 and non-competitive inhibition of Pgp by the verapamil and norverapamil enantiomers were incorporated based on in vitro literature. The resulting DDI performance was demonstrated by prediction of DDIs with midazolam, digoxin, rifampicin, and cimetidine, with 21/22 predicted DDI AUC ratios or C_trough_ ratios within 1.5-fold of the observed values. The thoroughly built and qualified model will be freely available in the Open Systems Pharmacology model repository to support model-informed drug discovery and development.

## 1. Introduction

Verapamil is a voltage-dependent calcium channel blocker (class-IV antiarrhythmic agent), used to treat hypertension, angina pectoris, and supraventricular tachycardia. Approved in the United States since 1981, it was still the 145th most prescribed drug in the U.S. in 2017, with over 4 million prescriptions [1]. Verapamil inhibits cytochrome P450 3A4 (CYP3A4) and P-glycoprotein (Pgp), and therefore care should be exercised when verapamil is co-administered with drugs that are substrates of CYP3A4 or Pgp. However, the inhibitory potential of racemic R-/S-verapamil and of the less cardioactive R-verapamil (“dexverapamil”) [2,3] towards Pgp is also leveraged advantageously to improve the delivery of anti-cancer drugs [4,5,6]. The potency of newly developed Pgp inhibitors for the reversal of cancer multidrug resistance is routinely compared to the inhibitory potential of verapamil as a reference and benchmark.

In addition to its administration as a cardiovascular therapeutic and as a chemosensitizer together with anti-cancer drugs, verapamil is widely used in drug–drug interaction (DDI) studies and is recommended by the FDA as a moderate clinical CYP3A4 index inhibitor and as a clinical Pgp inhibitor [7]. The CYP3A4 DDIs are caused by mechanism-based inactivation of CYP3A4 by R-verapamil, S-verapamil, R-norverapamil, and S-norverapamil [8,9]. The Pgp DDIs are caused by non-competitive inhibition of Pgp by these four entities [10,11,12,13]. Apart from the intended impact of verapamil in DDI studies, pharmacokinetic DDIs in patients treated with verapamil are clinically relevant. The CYP3A4 substrate midazolam shows a 2.9-fold increase of its area under the plasma concentration-time curve (AUC) during co-administration with verapamil [14]. The Pgp substrate and narrow therapeutic index drug digoxin shows a 1.5-fold increase of its AUC during co-administration with verapamil [15] and a warning is issued in the verapamil label to adjust the dose of digoxin during verapamil therapy [16].

Verapamil is a BCS Class I drug of high solubility and high permeability. It is positively charged at physiological pH, has a chiral center at C2, and is administered as racemic mixture (1:1) of R- and S-verapamil. The enantiomers exhibit different pharmacokinetic and pharmacodynamic properties, with a 10-fold higher therapeutic potency of the S-enantiomer [17]. Although >90% of an oral dose of verapamil is absorbed, bioavailability is only 10–22% due to high first-pass metabolism [18], with ≤4% excreted unchanged in the urine [19]. Verapamil is stereoselectively bound to plasma proteins and stereoselectively metabolized, mainly by CYP3A4, resulting in 2-fold higher plasma concentrations of R-verapamil following intravenous administration of the racemate, and even 5-fold higher plasma concentrations of R-verapamil following oral administration of the racemate [17]. The main metabolic pathway is N-demethylation by CYP3A4, not disturbing the chiral center and producing R- and S-norverapamil with plasma concentrations that are barely detectable following intravenous administration, but that are equal to or exceeding those of the parent drug enantiomers following oral administration of verapamil [20]. Norverapamil assumedly retains about 20% of the vasodilating activity of verapamil (studied in dogs) and is itself predominantly metabolized by CYP3A4 [9,20].

The purpose of this study was to build and evaluate a whole-body physiologically based pharmacokinetic (PBPK) model of verapamil that mechanistically describes and predicts the nonlinear pharmacokinetics of verapamil and its DDIs. The R- and S-enantiomers of verapamil and norverapamil with their stereospecific plasma protein binding, metabolism and mechanism-based inactivation of CYP3A4 are represented individually and the model was qualified for DDI prediction with the CYP3A4 and Pgp victim drugs midazolam and digoxin, as well as with the CYP3A4 perpetrator drugs rifampicin and cimetidine. The model will be shared in the Open Systems Pharmacology model repository (www.open-systems-pharmacology.org) as a tool for the pharmacometric analysis of racemic verapamil and its enantiomers (e.g., dexverapamil) and for the investigation and prediction of verapamil CYP3A4 and Pgp DDIs during drug development and labeling. The Supplementary Materials to this manuscript were compiled as one comprehensive reference manual, providing documentation of the complete model performance assessment.

## 2. Materials and Methods

### 2.1. Software

The PBPK model was developed using the open-source PK-Sim^®^ and MoBi^®^ modeling software (Open Systems Pharmacology Suite 8.0, released under the GPLv2 license by the Open Systems Pharmacology community, www.open-systems-pharmacology.org). Published clinical study data were digitized with GetData Graph Digitizer 2.26.0.20 (© S. Fedorov). Model parameter optimization (Levenberg–Marquardt algorithm using multiple starting values) and sensitivity analysis were performed in PK-Sim^®^. Pharmacokinetic parameters and model performance measures were calculated in R 3.6.3 (The R Foundation for Statistical Computing, Vienna, Austria). Plots were generated in R and RStudio 1.2.5033 (RStudio PBC, Boston, MA, USA).

### 2.2. Clinical Data

Clinical studies of intravenous and oral administration in single- and multiple-dose regimens were collected and digitized from literature [21], complemented by unpublished verapamil plasma concentration-time profiles of two clinical trials previously conducted at Boehringer Ingelheim [22,23]. Both studies were performed in accordance with the Declaration of Helsinki and its later amendments. The first study [22] was approved by the local Independent Ethics Committee (Ethikkommission der Landesärztekammer Baden-Württemberg, Stuttgart, Germany) and by the Federal Institute for Drugs and Medical Devices (2008-039, 4034097, NCT02171533, EudraCT 2008-001021-34); the second study [23] was approved by the local Independent Ethics Committee (Ethikkommission der Ärztekammer Hamburg, Hamburg, Germany) and by the Federal Institute for Drugs and Medical Devices (PVN5656, 4042346, NCT03307252, EudraCT 2017-001549-29). All subjects gave their informed consent before they participated in the studies. In addition to verapamil plasma concentration-time profiles, measured fraction excreted in urine data were included for model development.

The gathered verapamil plasma profiles were divided into a training dataset, used for model building and parameter optimization, and a test dataset, used for model evaluation. To build the training dataset, clinical studies were selected to include intravenous and oral administration over the entire dosing range, as well as fraction excreted in urine data. If multiple studies of the same dose were available, studies with many participants, modern bioanalytical methods and frequent as well as late sampling were chosen for the training dataset. The remaining studies were assigned to the test dataset. The allocation of the utilized clinical studies to either training or test dataset is documented in the clinical study table, and the data of all included clinical studies are shown in semilogarithmic as well as linear plots in the Supplementary Materials and provided in the released PK-Sim^®^ file.

### 2.3. PBPK Model Building

Verapamil model building was started with a comprehensive literature search for physicochemical parameters and information on verapamil absorption, distribution, metabolism, and excretion (ADME) processes. This information was used to develop the model in predict-learn-confirm cycles, testing different reported values and the impact of different ADME processes.

Virtual mean individuals to simulate the collected clinical studies were generated according to the published demographic information, using the reported age, sex, ethnicity, body weight, and height, if available. If no information was provided, a default value was substituted (30 years of age, male, European, mean body weight and height characteristics from the PK-Sim^®^ population database).

Metabolic enzymes and transporters for the disposition of verapamil were implemented in agreement with the current literature and the PK-Sim^®^ expression database [24], to define their relative expression in each of the 27 compartments of the virtual individuals. Details on the distribution and localization of the implemented enzymes and transporters are provided in the system-dependent parameter table in the Supplementary Materials. Model input parameters that could not be informed from literature were optimized by fitting the model simulations of all studies assigned to the training dataset simultaneously to their respective observed data.

### 2.4. PBPK Model Evaluation

Model performance was evaluated with various methods. First, predicted plasma concentration-time profiles were compared to the profiles measured in the respective clinical studies. Second, the predicted plasma concentration values of all studies were plotted against their corresponding observed values in goodness-of-fit plots.

In addition, model performance was evaluated by comparison of predicted to observed AUC and maximum plasma concentration (C_max_) values. All AUC values (predicted as well as observed) were calculated from the time of drug administration to the time of the last concentration measurement (AUC_last_).

As quantitative measures of the model performance, the mean relative deviation (MRD) of all predicted plasma concentrations (Equation (1)) and the geometric mean fold error (GMFE) of all predicted AUC_last_ and C_max_ values (Equation (2)) were calculated. MRD and GMFE values ≤ 2 characterize an adequate model performance.

(1) $${\mathrm{MRD}=10}^{x};\;x=\sqrt{\frac{\sum_{i=1}^{k}{\;(\log}_{10}c_{\mathrm{predicted},i}{\;-\;\log}_{10}c_{\mathrm{observed},i}{)}^{2}}{k}}$$

where c_predicted,i_ = predicted plasma concentration, c_observed,i_ = corresponding observed plasma concentration, k = number of observed values.

(2) $${\mathrm{GMFE}=10}^{x};\;x=\frac{\sum_{i=1}^{m}{\;|\;\log}_{10}\;\left(\frac{{\mathrm{predicted}\;\mathrm{PK}\;\mathrm{parameter}}_{i}}{{\mathrm{observed}\;\mathrm{PK}\;\mathrm{parameter}}_{i}}\right)|\;}{m}$$

where predicted PK parameter_i_ = predicted AUC_last_ or C_max_ value, observed PK parameter_i_ = corresponding observed AUC_last_ or C_max_ value, m = number of studies.

Furthermore, physiological plausibility of the parameter estimates and sensitivity analysis results were assessed. A detailed description of the sensitivity calculation is provided in the Supplementary Materials.

### 2.5. DDI Modeling

To mechanistically model the DDIs, the interaction type (competitive inhibition, non-competitive inhibition, mechanism-based inactivation, induction, etc.) and the corresponding in vitro interaction parameters were extracted from literature. These processes were then incorporated into the perpetrator PBPK models, to dynamically compute the impact of the perpetrator on the victim drug. The mathematical implementation of the different interaction types is shown in the Supplementary Materials.

### 2.6. DDI Modeling Evaluation

The DDI modeling performance was assessed by comparison of predicted versus observed plasma concentration-time profiles of the victim drugs, administered alone and during perpetrator co-administration. Furthermore, predicted DDI AUC_last_ ratios (Equation (3)) and DDI C_max_ ratios (Equation (4)) were evaluated.

(3) $$\mathrm{DDI}\;{\mathrm{AUC}}_{\mathrm{last}}\;\mathrm{ratio}=\frac{{\mathrm{AUC}}_{\mathrm{last}}\;\mathrm{victim}\;\mathrm{drug}\;\mathrm{during}\;\mathrm{co}-\mathrm{administration}}{{\mathrm{AUC}}_{\mathrm{last}}\;\mathrm{victim}\;\mathrm{drug}\;\mathrm{control}}$$

(4) $$\mathrm{DDI}\;C_{\max}\;\mathrm{ratio}=\frac{C_{\max}\;\mathrm{victim}\;\mathrm{drug}\;\mathrm{during}\;\mathrm{co}-\mathrm{administration}}{C_{\max}\;\mathrm{victim}\;\mathrm{drug}\;\mathrm{control}}$$

As a quantitative measure of the prediction accuracy, GMFE values of the predicted DDI AUC_last_ ratios and DDI C_max_ ratios were calculated according to Equation (2).

## 3. Results

### 3.1. Verapamil PBPK Model Building and Evaluation

For PBPK model building and evaluation, 45 clinical studies of intravenous or oral administration were utilized, covering a broad dosing range of 0.1–250 mg verapamil, including seven studies with only one of the verapamil enantiomers (R- or S-verapamil) administered. A table listing all utilized clinical studies is provided in the Supplementary Materials.

To mechanistically describe the pharmacokinetics of racemic verapamil, the enantiomers of verapamil and norverapamil are represented separately in the model. This approach allows the incorporation of the enantioselective plasma protein binding and CYP3A4 metabolism as well as the simulation of clinical studies using enantiopure R- or S-verapamil. As the majority of the published clinical studies administered racemic verapamil (50% R-verapamil + 50% S-verapamil) and reported the plasma concentrations of total verapamil (sum of R- and S-verapamil) and total norverapamil (sum of R- and S-norverapamil), so-called “observers” were implemented into the PK-Sim file that conveniently and directly display total verapamil and total norverapamil in blood plasma and as fraction of dose excreted unchanged in urine. These observers are auxiliary simulation outputs, that add up the simulated plasma concentrations or fractions of dose of either R- and S-verapamil or those of R- and S-norverapamil, so that they can be instantly compared to clinical data reporting total verapamil or total norverapamil.

**Figure 1:**
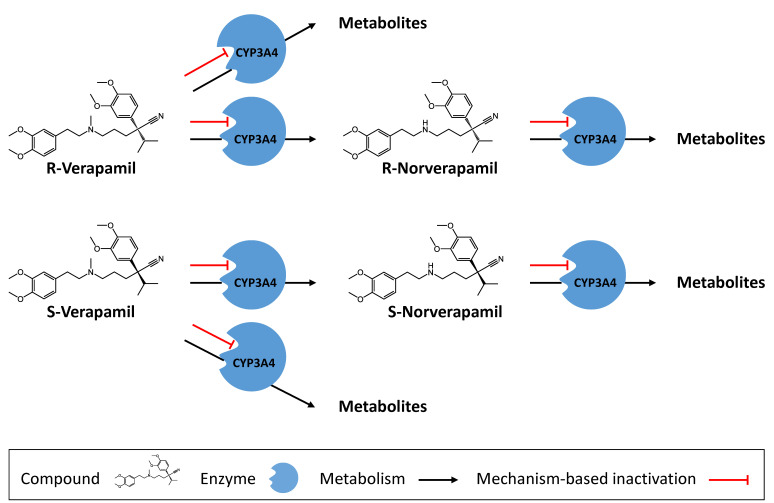
Verapamil metabolism and CYP3A4 inactivation. R-verapamil, S-verapamil, R-norverapamil, and S-norverapamil are either metabolized by CYP3A4 or they destroy a CYP3A4 molecule in an irreversible mechanism-based inactivation, depleting the CYP3A4 pool until new enzyme is synthesized. R- and S-verapamil are metabolized via two different CYP3A4-mediated pathways: N-demethylation (to produce R- and S-norverapamil) or N-dealkylation. The metabolites that are not modeled as stand-alone compounds (D617 and D620) are assumed to be no inhibitors of CYP3A4 and Pgp according to literature reports [8,11].

The processes implemented to describe the pharmacokinetics of verapamil are enantioselective plasma protein binding, enantioselective metabolism by CYP3A4 to different metabolites, non-stereospecific transport by Pgp (according to literature [25,26,27]), and passive glomerular filtration. R-verapamil is metabolized by CYP3A4 via two different pathways, either to generate R-norverapamil, or to produce other metabolites such as “R-D617” that are not represented in the model as stand-alone compounds. The generated metabolite R-norverapamil is eliminated via CYP3A4 as well. S-verapamil is also metabolized by CYP3A4 via two different pathways, either to generate S-norverapamil or to produce other metabolites such as “S-D617” that are not represented in the model as stand-alone compounds. The generated metabolite S-norverapamil is eliminated via CYP3A4 as well. All four modeled entities (R-verapamil, S-verapamil, R-norverapamil, S-norverapamil) are mechanism-based inactivators of CYP3A4; this auto-inactivation was implemented using in vitro values [9]. A schematic illustration of CYP3A4 metabolism and inactivation is given in Figure 1.

In addition to their CYP3A4 metabolism, all four compounds are substrates and non-competitive inhibitors of Pgp [10,12,13,28]; but contrary to their CYP3A4 metabolism, no stereospecificity of their Pgp transport was found in vitro or in vivo [25,26,27]. Small fractions of an orally administered verapamil dose are excreted in the urine as verapamil or norverapamil (3–4% as total verapamil and 6% as total norverapamil [19]). The parameters of the final enantiomer-parent-metabolite model are summarized in the verapamil and norverapamil drug-dependent parameter tables (Table 1 and Table 2). Details on the implemented drug metabolizing enzymes and transporters are provided in the system-dependent parameter table in the Supplementary Materials.

**Figure 2:**
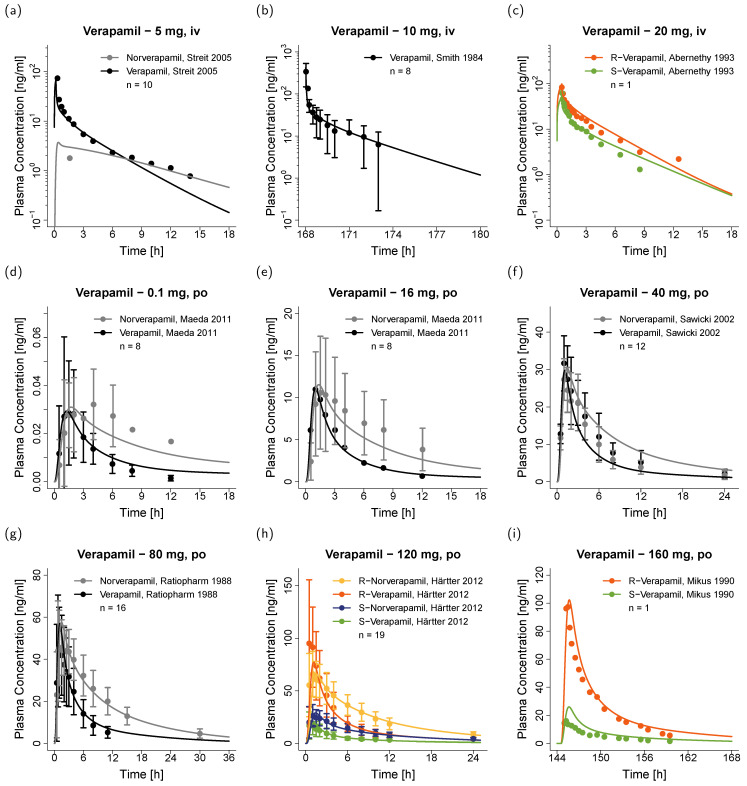
Verapamil plasma concentrations. Model predictions of verapamil and norverapamil plasma concentration-time profiles of representative (**a**–**c**) intravenous and (**d**–**i**) oral studies, compared to observed data [22,42,43,44,45,46,47,48]. Predictions are shown as lines, observed data are shown as dots ± SD. Black = total verapamil, grey = total norverapamil, orange = R-verapamil, yellow = R-norverapamil, green = S-verapamil, blue = S-norverapamil. Details on the study protocols and model predictions of the remaining studies used for model building and evaluation are provided in the Supplementary Materials. iv: intravenous, po: oral.

**Figure 3:**
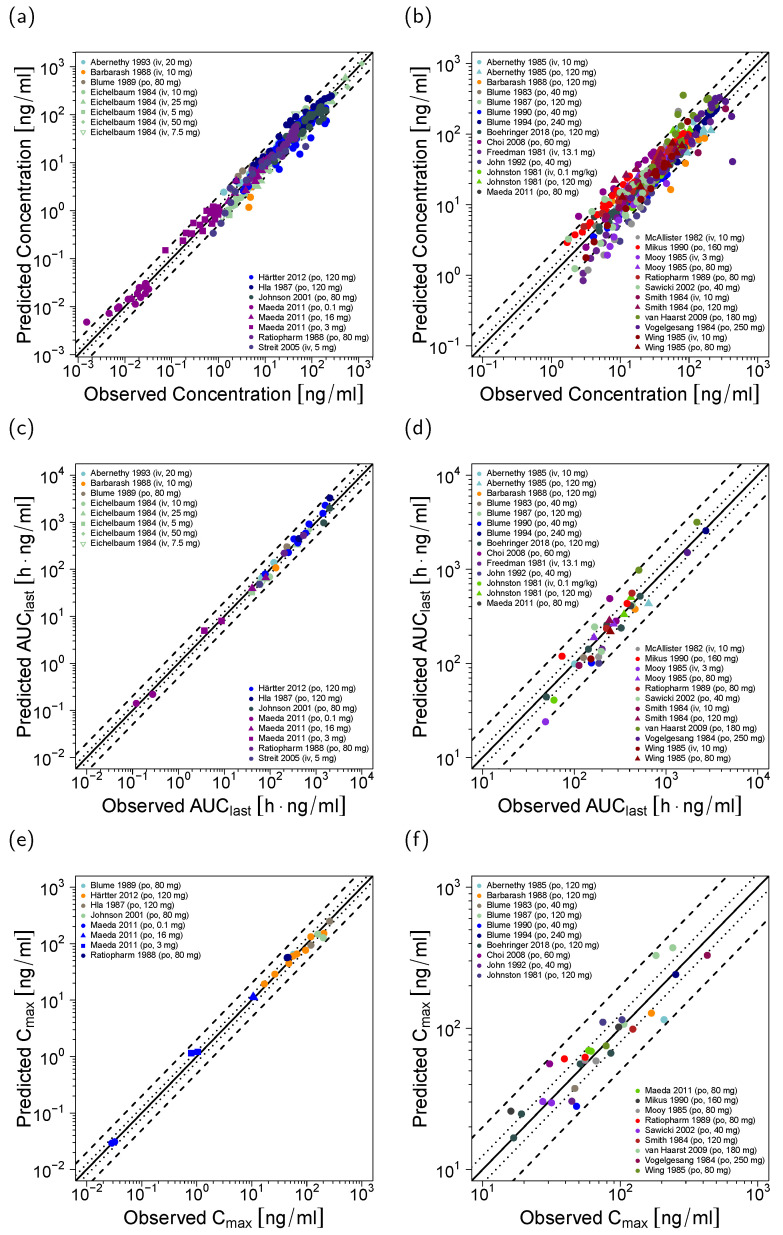
Goodness-of-fit plots illustrating the model performance for the training dataset (left) and the test dataset (right). Shown are predicted compared to observed values of (**a**,**b**) all measured verapamil and norverapamil plasma concentrations, (**c**,**d**) all AUC_last_ values, and (**e**,**f**) all C_max_ values. The solid line marks the line of identity, dotted lines indicate 1.25-fold, dashed lines indicate 2-fold deviation. Details on all studies are provided in the Supplementary Materials. iv: intravenous, po: oral.

The good model performance is illustrated in Figure 2, showing predicted plasma concentration-time profiles compared to the corresponding clinical data of representative studies. Predicted compared to observed plasma profiles of all 45 modeled studies are shown in the Supplementary Materials (semi-logarithmic as well as linear plots). Furthermore, plasma concentration goodness-of-fit plots are presented in Figure 3a,b and MRD values for all studies are listed in the Supplementary Materials.

In addition, the good model performance is demonstrated in plots (Figure 3c–f) and tables (Supplementary Materials) comparing the predicted to observed AUC_last_ and C_max_ values, showing low overall GMFE values of 1.24 (AUC_last_) and 1.22 (C_max_). A total of 67/68 of the predicted AUC_last_ values (some of the 45 studies report more than one analyte, for example total verapamil and total norverapamil) and 51/51 of the predicted C_max_ values are within the 2-fold acceptance limits.

Sensitivity analysis of a simulation of 120 mg orally administered racemic verapamil with a sensitivity threshold of 0.5 revealed that the only optimized parameter value that the predicted total verapamil or total norverapamil plasma concentrations are sensitive to, is the R-norverapamil → D620 CYP3A4 catalytic rate constant. The predicted total verapamil plasma concentrations are sensitive to the values of fraction unbound of R-verapamil and S-verapamil (both fixed to literature values), and the predicted total norverapamil plasma concentrations are sensitive to the values of fraction unbound of R-norverapamil and S-norverapamil (both fixed to the literature values of verapamil) as well as to the CYP3A4 catalytic rate constant for the metabolism of R-norverapamil (optimized). The full quantitative results of the sensitivity analysis are provided in the Supplementary Materials.

### 3.2. Verapamil DDI Modeling and Evaluation

**Figure 4:**
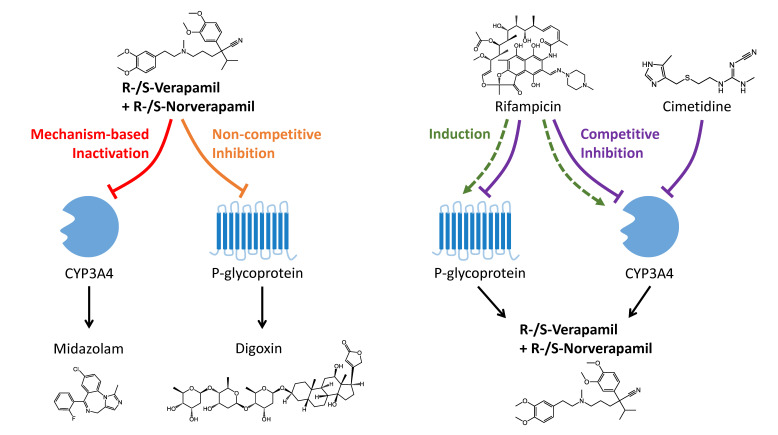
Schematic illustration of the modeled drug–drug interactions. Verapamil acts as the perpetrator in the drug–drug interactions (DDIs) with midazolam and digoxin, whereas it is the victim drug in the DDIs with rifampicin and cimetidine. Metabolism and transport of the victim drugs are shown as black arrows. Mechanism-based inactivation is shown as a red line, non-competitive inhibition as an orange line, competitive inhibition as purple lines, and induction as green dashed arrows.

Verapamil DDI model qualification was accomplished using a total of 22 clinical DDI studies with two different victim drugs (midazolam and digoxin) and two different perpetrators (rifampicin and cimetidine). An overview of the modeled DDI combinations is shown in Figure 4. The parameters of the previously developed PBPK models of midazolam, digoxin, rifampicin [49], and cimetidine [50] are reproduced in the Supplementary Materials.

The verapamil-midazolam DDI was predicted as mechanism-based inactivation of CYP3A4 midazolam metabolism using the intrinsic mechanism-based auto-inactivation processes that are part of the verapamil model to describe the inactivation of CYP3A4 by R-verapamil, S-verapamil, R-norverapamil, and S-norverapamil. KI (corrected for binding in the microsomal assay) and kinact values of these inactivation processes were obtained from in vitro literature [9] and are listed in the verapamil and norverapamil drug-dependent parameter tables (Table 1 and Table 2).

The verapamil-digoxin DDI was modeled as non-competitive inhibition of Pgp digoxin transport by R-verapamil, S-verapamil, R-norverapamil, and S-norverapamil. Non-stereospecific, equipotent inhibition by all four compounds was assumed, as described in the literature [10,12]; the Ki = 0.038 µmol/L (also listed in Table 1 and Table 2) was optimized using one of the 10 clinical verapamil-digoxin DDI studies [51] and then applied to predict the remaining nine studies.

The rifampicin-verapamil DDI was predicted as induction of CYP3A4 verapamil metabolism and Pgp verapamil transport by rifampicin, with simultaneous competitive inhibition of CYP3A4 and Pgp [49]. The parameter values to model these interactions were obtained from literature (values and references are listed in the rifampicin drug-dependent parameter table in the Supplementary Materials) and have been qualified in previous DDI analyses [49,52].

The cimetidine-verapamil DDI was predicted as competitive inhibition of CYP3A4 verapamil metabolism by cimetidine. The Ki = 268.0 µmol/L (listed in the cimetidine drug-dependent parameter table in the Supplementary Materials) for this weak inhibition was obtained from literature [53] and has been qualified previously by prediction of the cimetidine-midazolam DDI [50].

**Figure 5:**
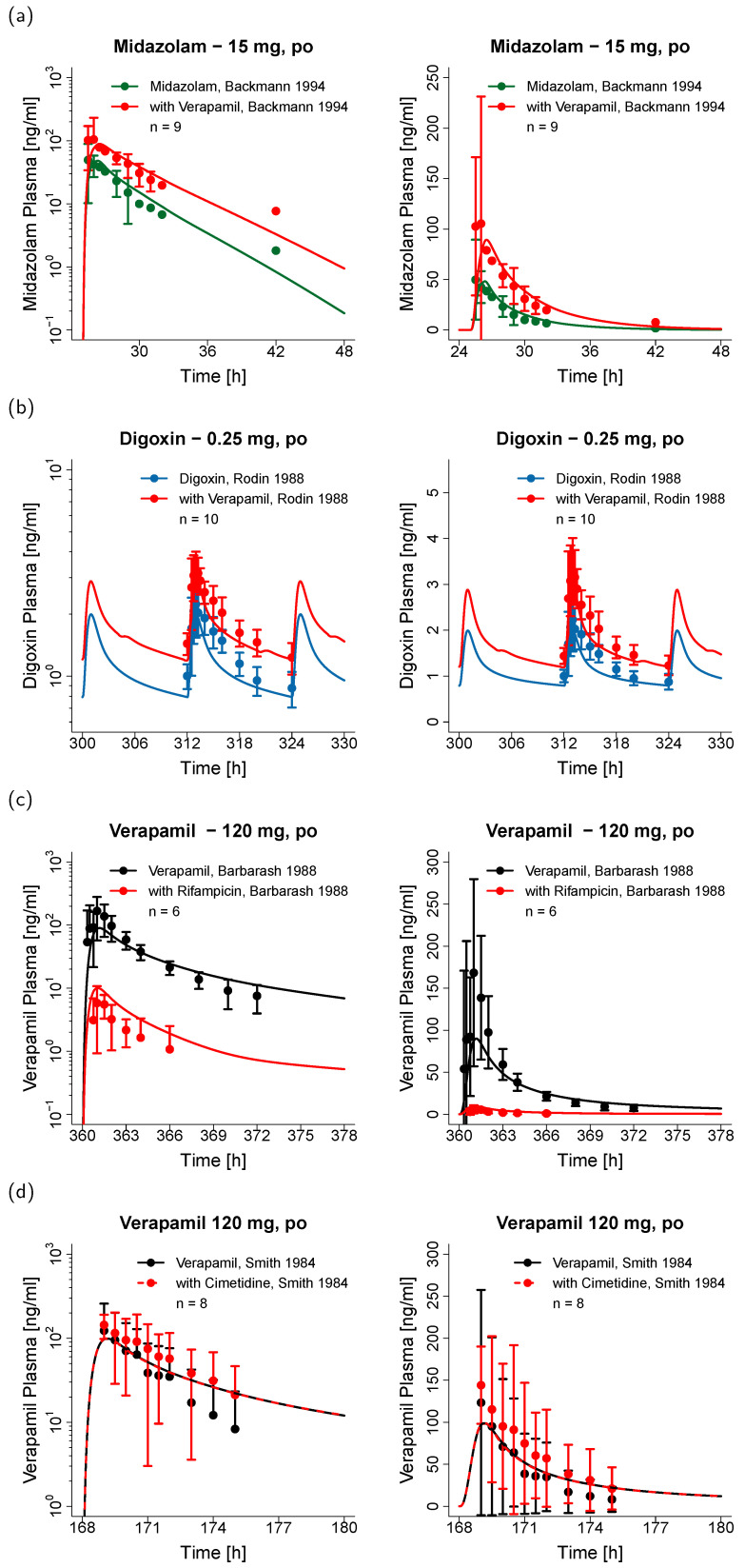
Victim drug plasma concentrations of the modeled drug-drug interactions. (**a**) Verapamil-midazolam DDI model performance; (**b**) verapamil-digoxin DDI model performance; (**c**) rifampicin-verapamil DDI model performance, and (**d**) cimetidine-verapamil DDI model performance for representative studies, shown in semilogarithmic (left) and linear plots (right) and compared to the corresponding observed data [14,15,43,54]. Predictions are shown as lines, observed data are shown as dots ± SD. Green, blue, and black = victim drug plasma concentrations without perpetrator co-administration, red = victim drug plasma concentrations during perpetrator treatment. Details on the study protocols and model predictions of the remaining DDI studies are provided in the Supplementary Materials. po: oral.

The DDI model performance for the four different DDIs is illustrated in Figure 5, showing predicted victim drug plasma concentration-time profiles (before and during DDI) compared to the corresponding clinical data of representative studies (one for each drug combination). Predicted compared to observed plasma profiles of all 22 modeled DDI studies are shown in the Supplementary Materials (semi-logarithmic as well as linear plots). The successfully predicted DDI regimens include three studies using multiple doses of verapamil (240 mg daily) with single doses of midazolam (intravenous or oral), 10 studies using single (120 mg) or multiple (240–360 mg daily) doses of verapamil with single (intravenous or oral) or multiple (oral) doses of digoxin, two studies using multiple doses of rifampicin (600 mg daily) with single doses of verapamil (intravenous or oral), and seven studies using multiple doses of cimetidine (800–1200 mg) with single doses of verapamil (intravenous or oral). Further details on the dosing schedules are provided in the DDI study tables in the Supplementary Materials.

**Figure 6:**
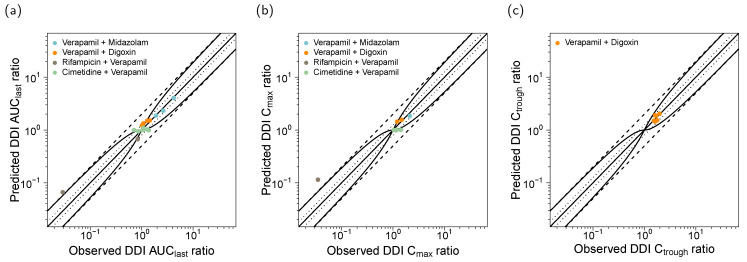
Correlation of predicted and observed DDI ratios. Model predicted (**a**) DDI AUC_last_ ratios, (**b**) DDI C_max_ ratios, and (**c**) DDI C_trough_ ratios, compared to the corresponding clinically observed ratios of all 22 analyzed DDI studies. The different colors indicate the verapamil-midazolam DDI (blue), the verapamil-digoxin DDI (orange), the rifampicin-verapamil DDI (grey), and the cimetidine-verapamil DDI (green). The straight solid line marks the line of identity, the curved solid lines show the prediction acceptance limits proposed by Guest et al. [55]. Dotted lines indicate 1.25-fold, dashed lines indicate 2-fold deviation. Details on the study protocols and the values of the plotted DDI ratios are provided in the Supplementary Materials. iv: intravenous, po: oral.

The predicted DDI AUC_last_ ratios are close to the observed values (see Figure 6), with overall GMFEs of 1.06, 1.17, 1.68, and 1.17 for the four modeled DDIs (verapamil with midazolam, digoxin, rifampicin, and cimetidine, respectively). The predicted DDI C_max_ ratios show GMFE values of 1.14, 1.13, 3.32, and 1.17, respectively. A total of 21/22 of the predicted DDI AUC_last_ ratios or DDI trough plasma concentration (C_trough_) ratios are within 1.5-fold of the observed values; 7/8 of the predicted DDI C_max_ ratios are within 1.5-fold of the observed values. The full quantitative evaluation with all predicted and observed ratios, DDI GMFE values and ranges is presented in the Supplementary Materials.

Based on this DDI evaluation, the verapamil model is considered applicable to predict the impact of verapamil on CYP3A4 and Pgp victim drugs. Parameters to model the inhibition of further metabolic enzymes or transporters are not yet implemented, as this requires evaluation of the resulting DDI predictions with clinical data, which is beyond the scope of this study. However, the implementation of additional interaction parameters is technically a simple and straight-forward extension of the current model.

## 4. Discussion

Verapamil is mostly administered in its racemic form, but the two enantiomers show different pharmacokinetic properties. Following intravenous administration of the racemate, 2-fold higher concentrations of R-verapamil are found in plasma and following oral administration, the plasma concentrations of R-verapamil are even 5-fold higher than those of S-verapamil [17]. The metabolism of R- and S-verapamil, and also that of their main metabolites R- and S-norverapamil, is catalyzed primarily by CYP3A4, with all four compounds being mechanism-based inactivators of CYP3A4. For a mechanistic description of the pharmacokinetics of verapamil and its drug–drug interactions, these four compounds were incorporated into the model, using in vitro values as input for the stereospecific plasma protein binding, CYP3A4 metabolism and mechanism-based CYP3A4 auto-inactivation, and clinical studies that quantified the R- and S-enantiomers of verapamil and norverapamil to build and evaluate the model [17,22,23,44,48,56].

Although metabolism of verapamil by CYP2C8 could be observed in vitro [41,57], the CYP2C8 affinity and catalytic rate were much lower than those of CYP3A4 in the same experimental setting (measured with recombinant CYP enzymes) [57]. The addition of verapamil metabolism by CYP2C8 to the model resulted in an underestimation of the rifampicin-verapamil DDI and was therefore not retained. Norverapamil is reported to be predominantly metabolized by CYP3A4 [9].

The role of Pgp in the pharmacokinetics of verapamil is difficult to assess. Some in vitro studies report that transport of verapamil and norverapamil could only be observed in Pgp overexpressing cells [11], whereas other studies demonstrated verapamil transport in normal Caco-2 cells as well as in overexpressing cells [34]. The weak impact of Pgp on verapamil in vitro and in vivo is explained by the high passive permeability of verapamil and the early saturation of Pgp [11,25] that together prevent a significant effect of Pgp on verapamil absorption and bioavailability in vivo [25].

The inhibitory potential of verapamil on Pgp is even more challenging to assess, because Pgp has at least two different binding sites for verapamil which accommodate simultaneous binding [13,35,58], and because the inhibition of Pgp by verapamil has been investigated in vitro with many different techniques and calculation methods [59]. This may explain the multitude of different reported Ki and IC50 values in the literature, with 32 entries in the University of Washington Drug Interaction Database for the verapamil inhibition of Pgp digoxin transport alone, which range from 0.06 to 224 µmol/L. Underprediction of the verapamil-digoxin DDI, applying a low verapamil in vitro Ki value of 0.1 µmol/L, has been reported previously [60], and was confirmed in the presented study. Optimization of this Ki value (to 0.038 µmol/L), using one of the clinical verapamil-digoxin DDI studies, resulted in an accurate description of all 10 clinical studies, with predicted DDI AUC_last_, C_max_, and C_trough_ ratios within 1.25-fold of the observed data (values for all studies are listed in the Supplementary Materials). One hypothesis to explain this underprediction of the in vivo DDI using in vitro values is that digoxin is not only a substrate of Pgp, but additionally requires active uptake by an as yet unidentified transporter, as has been observed in human hepatocytes, Caco-2, MDCK, and HEK293 cells [61,62,63]. This unidentified transporter might be inhibited by verapamil [62], but is not yet incorporated into the applied digoxin model and therefore was not inhibited in the presented verapamil-digoxin DDI simulations.

The other three modeled DDIs are entirely predicted, using interaction parameters from literature. The verapamil-midazolam DDI is very well described, applying the intrinsic CYP3A4 mechanism-based auto-inactivation parameters that are part of the verapamil model to inhibit the metabolism of midazolam (Figure 6 and Supplementary Materials). The rifampicin-verapamil DDI with orally administered verapamil is underpredicted, with a predicted DDI AUC_last_ ratio of 0.07 compared to an observed ratio of 0.03, and a predicted DDI C_max_ ratio of 0.11 against an observed ratio of 0.03. This DDI was modeled applying parameters for the rifampicin induction of CYP3A4 and Pgp that have been qualified previously [49]. The modeled clinical study [54] was designed with a dose gap of 12 h between the administration of rifampicin and verapamil, to avoid the competitive inhibition of metabolic enzymes and transporters by rifampicin. Furthermore, verapamil was given as a single dose, preventing effects of verapamil on rifampicin exposure. This leaves induction of an additional metabolic enzyme or transporter as a possible explanation. The cimetidine-verapamil DDI is well described, applying the competitive inhibition of CYP3A4 by cimetidine (Ki = 268.0 µmol/L [53]) that has been qualified previously by prediction of the cimetidine-midazolam DDI [50]. The model predicts no effect of cimetidine on verapamil except for the study with the highest dose of cimetidine and oral administration of verapamil [64], where a DDI AUC_last_ ratio of 1.10 was estimated. 6/7 of the corresponding clinical studies also report no significant effect of cimetidine on the AUC of verapamil [43,48,64,65]. Given a maximum cimetidine plasma concentration of 10 µmol/L, this weak CYP3A4 DDI most probably occurs at the intestinal level.

Previously published, well-established PBPK models of verapamil do not consider the inactivation of CYP3A4 by norverapamil [60], the stereospecific characteristics of the R- and S-enantiomers [60,66], or the prediction of Pgp-mediated DDIs [9,66]. The individual representation of the verapamil and norverapamil R- and S-enantiomers in the presented whole-body PBPK model allows its application for the prediction of verapamil and norverapamil exposure following administration of racemic or enantiopure verapamil or norverapamil, as well as the mechanistic implementation of the CYP3A4 MBI by all four compounds, using in vitro values to characterize their different inactivation potencies (S-norverapamil > S-verapamil > R-norverapamil > R-verapamil) [8,9]. The implementation of R- and S-norverapamil was enabled by the clinical data of two studies that quantified not only R- and S-verapamil but also R- and S-norverapamil following single and multiple dose verapamil administration [22,23].

The presented model can be applied to develop dose recommendations for cardiovascular patients, to help manage the DDI potential of verapamil. As a tool for drug development, it can be used to explore different study protocols during clinical DDI study design and to predict the outcome of untested clinical scenarios. In some cases, it might even be applied to predict the DDI of verapamil with an investigational drug to waive part of a clinical study. Furthermore, the model could be used to predict the exposure of cancer patients following administration of racemic verapamil (first generation chemosensitizer), R-verapamil, or norverapamil (second generation chemosensitizers). Future possible applications include the implementation of a tumor compartment using individual tumor biopsy Pgp expression information, to predict the delivery of anti-cancer drugs that are Pgp substrates during co-administration of verapamil, R-verapamil, or norverapamil.

## 5. Conclusions

A comprehensive and mechanistic, enantioselective parent-metabolite PBPK model of verapamil was established, which includes whole-body PBPK modeling of R-verapamil, S-verapamil, R-norverapamil, and S-norverapamil. The model reliably describes the nonlinear pharmacokinetics of verapamil and was thoroughly qualified to predict the verapamil-midazolam, verapamil-digoxin, rifampicin-verapamil, and cimetidine-verapamil DDIs. Model evaluation was transparently documented, showing the model performance for all 45 clinical verapamil and all 22 clinical DDI studies included in this analysis. The model will be shared in the Open Systems Pharmacology repository (www.open-systems-pharmacology.org) [67] to support verapamil therapy of cardiovascular disease, its application as multidrug resistance reversal agent, and to support DDI studies during drug development.

## Figures and Tables

**Table 1:** R- and S-verapamil drug-dependent parameters.

Parameter	Value	Unit	Source	Literature	Reference	Value	Unit	Source	Literature	Reference	Description
	R-Verapamil					S-Verapamil					
MW	454.611	g/mol	Lit.	454.611	[29]	454.611	g/mol	Lit.	454.611	[29]	Molecular weight
pKa (base)	8.75	-	Lit.	8.75	[30]	8.75	-	Lit.	8.75	[30]	Acid dissociation constant
Solubility (pH 6.54)	46.0	g/L	Lit.	46.0	[31]	46.0	g/L	Lit.	46.0	[31]	Solubility
logP	2.84 *	-	Optim.	3.79	[32]	2.84 *	-	Optim.	3.79	[32]	Lipophilicity
fu	5.1	%	Lit.	5.1	[33]	11.0	%	Lit.	11.0	[33]	Fraction unbound
CYP3A4 Km → Norv	19.59	µmol/L	Lit.	19.59 ^‡^	[9]	9.72	µmol/L	Lit.	9.72 ^‡^	[9]	Michaelis–Menten constant
CYP3A4 kcat → Norv	34.94	1/min	Optim.	-	-	26.17	1/min	Optim.	-	-	Catalytic rate constant
CYP3A4 Km → D617	35.34	µmol/L	Lit.	35.34 ^‡^	[9]	23.64	µmol/L	Lit.	23.64 ^‡^	[9]	Michaelis–Menten constant
CYP3A4 kcat → D617	43.98	1/min	Optim.	-	-	56.42	1/min	Optim.	-	-	Catalytic rate constant
Pgp Km	1.01	µmol/L	Lit.	1.01	[34]	1.01	µmol/L	Lit.	1.01	[34]	Michaelis–Menten constant
Pgp kcat	12.60 °	1/min	Optim.	-	-	12.60 °	1/min	Optim.	-	-	Transport rate constant
GFR fraction	1.00	-	Ass.	-	-	1.00	-	Ass.	-	-	Filtered drug in the urine
EHC cont. fraction	1.00	-	Ass.	-	-	1.00	-	Ass.	-	-	Bile fraction cont. released
CYP3A4 MBI KI	27.63	µmol/L	Lit.	27.63 ^‡^	[9]	3.85	µmol/L	Lit.	3.85 ^‡^	[9]	Conc. for 50% inactivation
CYP3A4 MBI kinact	0.038	1/min	Lit.	0.038	[9]	0.034	1/min	Lit.	0.034	[9]	Maximum inactivation rate
Pgp non-competitive Ki	0.038 *	µmol/L	Optim.	0.31	[35]	0.038 *	µmol/L	Optim.	0.31	[35]	Conc. for 50% inhibition
Partition coefficients	Diverse	-	Calc.	R&R	[36,37]	Diverse	-	Calc.	R&R	[36,37]	Cell to plasma partitioning
Cellular permeability	9.94 × 10^−2^ *	cm/min	Optim.	PK-Sim	[38]	9.94 × 10^−2^ *	cm/min	Optim.	PK-Sim	[38]	Perm. into the cellular space
Intestinal permeability	3.54 × 10^−6^ *	cm/min	Optim.	1.21 × 10^−5^	Calc.	3.54 × 10^−6^ *	cm/min	Optim.	1.21 × 10^−5^	Calc.	Transcellular intestinal perm.
SR tablet Weibull time	155.24	min	Optim.	-	[39]	155.24	min	Optim.	-	[39]	Dissolution time (50%)
SR tablet Weibull shape	2.37	-	Optim.	-	[39]	2.37	-	Optim.	-	[39]	Dissolution profile shape

* Assumed to be the same for all four compounds, ° assumed to be the same for R-/S-verapamil, ^‡^ in vitro values corrected for binding in the assay using fraction unbound to microsomal protein measurements from the same study, ass.: assumed, calc.: calculated, conc.: concentration, cont.: continuously, CYP3A4: cytochrome P450 3A4, D617: verapamil metabolite, EHC: enterohepatic circulation, GFR: glomerular filtration rate, lit.: literature, MBI: mechanism-based inactivation, Norv: norverapamil, optim.: optimized, perm.: permeability, Pgp: P-glycoprotein, PK-Sim: PK-Sim standard calculation method, R&R: Rodgers and Rowland calculation method, SR: sustained release formulation.

**Table 2:** R- and S-norverapamil drug-dependent parameters.

Parameter	Value	Unit	Source	Literature	Reference	Value	Unit	Source	Literature	Reference	Description
	R-Norverapamil					S-Norverapamil					
MW	440.584	g/mol	Lit.	440.584	[29]	440.584	g/mol	Lit.	440.584	[29]	Molecular weight
pKa (base)	8.75	-	Lit.	8.6–8.9	[40]	8.75	-	Lit.	8.6–8.9	[40]	Acid dissociation constant
logP	2.84 *	-	Optim.	-	-	2.84 *	-	Optim.	-	-	Lipophilicity
fu	5.1 ^a^	%	Ass.	-	-	11.0 ^b^	%	Ass.	-	-	Fraction unbound
CYP3A4 Km → D620	144.0	µmol/L	Lit.	144.0	[41]	36.0	µmol/L	Lit.	36.0	[41]	Michaelis–Menten constant
CYP3A4 kcat → D620	145.64	1/min	Optim.	-	-	41.10	1/min	Optim.	-	-	Catalytic rate constant
Pgp Km	1.01 *	µmol/L	Ass.	-	-	1.01 *	µmol/L	Ass.	-	-	Michaelis–Menten constant
Pgp kcat	3.39 °	1/min	Optim.	-	-	3.39 °	1/min	Optim.	-	-	Transport rate constant
GFR fraction	1.00	-	Ass.	-	-	1.00	-	Ass.	-	-	Filtered drug in the urine
EHC cont. fraction	1.00	-	Ass.	-	-	1.00	-	Ass.	-	-	Bile fraction cont. released
CYP3A4 MBI KI	6.10	µmol/L	Lit.	6.10 ^‡^	[9]	2.90	µmol/L	Lit.	2.90 ^‡^	[9]	Conc. for 50% inactivation
CYP3A4 MBI kinact	0.048	1/min	Lit.	0.048	[9]	0.080	1/min	Lit.	0.080	[9]	Maximum inactivation rate
Pgp non-competitive Ki	0.038 *	µmol/L	Optim.	0.30 ^c^	[11]	0.038 *	µmol/L	Optim.	0.30 ^c^	[11]	Conc. for 50% inhibition
Partition coefficients	Diverse	-	Calc.	R&R	[36,37]	Diverse	-	Calc.	R&R	[36,37]	Cell to plasma partitioning
Cellular permeability	9.94 × 10^−2^ *	cm/min	Optim.	PK-Sim	[38]	9.94 × 10^−2^ *	cm/min	Optim.	PK-Sim	[38]	Perm. into the cellular space
Intestinal permeability	3.54 × 10^−6^ *	cm/min	Optim.	1.40 × 10^−5^	Calc.	3.54 × 10^−6^ *	cm/min	Optim.	1.40 × 10^−5^	Calc.	Transcellular intestinal perm.

* Assumed to be the same for all four compounds, ° assumed to be the same for R-/S-norverapamil, ^‡^ in vitro values corrected for binding in the assay using fraction unbound to microsomal protein measurements from the same study, ^a^ assumed to be the same for R-verapamil/R-norverapamil, ^b^ assumed to be the same for S-verapamil/S-norverapamil, ^c^ IC50 with substrate conc. in the assay much smaller than the Pgp substrate Km, ass.: assumed, calc.: calculated, conc.: concentration, cont.: continuously, CYP3A4: cytochrome P450 3A4, D620: norverapamil metabolite, EHC: enterohepatic circulation, GFR: glomerular filtration rate, lit.: literature, MBI: mechanism-based inactivation, optim.: optimized, perm.: permeability, Pgp: P-glycoprotein, PK-Sim: PK-Sim standard calculation method, R&R: Rodgers and Rowland calculation method.

## Supplementary Materials

The following are available online at https://www.mdpi.com/1999-4923/12/6/556/s1, Electronic Supplementary Materials: A comprehensive reference manual, providing documentation of the complete model performance assessment.
