# Supplementary material for: A Mechanistic, Enantioselective, Physiologically Based Pharmacokinetic Model of Verapamil and Norverapamil, Built and Evaluated for Drug–Drug Interaction Studies
Source: Pharmaceutics. 2020 Jun 16;12(6):556. doi: 10.3390/pharmaceutics12060556 (PMC7355632; doi:10.3390/pharmaceutics12060556)
Supplement: Supplementary file 1 [file pharmaceutics-12-00556-s001.pdf]

pharmaceutics

A mechanistic, enantioselective, physiologically based pharmacokinetic model of verapamil and norverapamil, built and evaluated for drug-drug interaction studies

Electronic Supplementary Document (ESD)

Nina Hanke [1], Denise Türk [1], Dominik Selzer [1], Sabrina Wiebe [2,3], Éric Fernandez [2], Peter Stopfer [2], Valerie Nock [2] and Thorsten Lehr [1]

[1] Clinical Pharmacy, Saarland University, Saarbrücken, Germany
[2] Translational Medicine and Clinical Pharmacology, Boehringer Ingelheim Pharma GmbH & Co. KG, Biberach, Germany
[3] Clinical Pharmacology and Pharmacoepidemiology, Heidelberg University Hospital, Heidelberg, Germany

Funding
This project has received funding from Boehringer Ingelheim Pharma GmbH & Co. KG and from the German Federal Ministry of Education and Research (BMBF), grant numbers 03XP0196 ("NanoCare4.0 – Anwendungssichere Materialinnovationen") and 031L0161C ("OSMOSES"). The APC was funded by the Deutsche Forschungsgemeinschaft (DFG, German Research Foundation) and Saarland University within the funding programme "Open Access Publishing".

Conflict of Interest
Sabrina Wiebe, Éric Fernandez, Peter Stopfer and Valerie Nock are employees of Boehringer Ingelheim Pharma GmbH & Co. KG. Thorsten Lehr has received research grants from Boehringer Ingelheim Pharma GmbH & Co. KG. and from the German Federal Ministry of Education and Research. Nina Hanke, Denise Türk and Dominik Selzer declare no conflict of interest.

Corresponding Author
Prof. Dr. Thorsten Lehr
Clinical Pharmacy, Saarland University
Campus C2 2, 66123 Saarbrücken, Germany
ORCID: 0000 0002 8372 1465
Phone: +49 681 302 70255
Email: thorsten.lehr@mx.uni-saarland.de

Contents

1 Physiologically based pharmacokinetic (PBPK) modeling **4**
1.1 PBPK model building 4
1.2 Virtual individuals 4
1.3 PBPK model evaluation 4
1.4 PBPK model sensitivity analysis 5
1.5 Mathematical implementation of drug-drug interactions 5
1.5.1 Competitive inhibition 5
1.5.2 Non-competitive inhibition 5
1.5.3 Mechanism-based inactivation 6
1.5.4 Induction 6

2 Verapamil **8**
2.1 PBPK model development 8
2.2 Verapamil clinical studies 9
2.3 Verapamil and norverapamil drug-dependent parameters 11
2.4 Profiles 13
2.5 Model evaluation 24
2.5.1 Predicted concentrations versus observed concentrations goodness-of-fit plots . 24
2.5.2 Mean relative deviation of plasma concentration predictions 25
2.5.3 AUC and C_{max} goodness-of-fit plots 27
2.5.4 Geometric mean fold error of predicted AUC and C_{max} values 28
2.5.5 Sensitivity analysis 30

3 Verapamil-midazolam drug-drug interaction (DDI) **32**
3.1 DDI modeling 32
3.2 Midazolam drug-dependent parameters 32
3.3 Verapamil-midazolam clinical DDI studies 33
3.4 Profiles 34
3.5 Model evaluation 35
3.5.1 DDI AUC and C_{max} ratio goodness-of-fit plots 35
3.5.2 Geometric mean fold error of predicted DDI AUC and C_{max} ratios 36

4 Verapamil-digoxin drug-drug interaction (DDI) **37**
4.1 DDI modeling 37
4.2 Digoxin drug-dependent parameters 37
4.3 Verapamil-digoxin clinical DDI studies 38
4.4 Profiles 39
4.5 Model evaluation 44
4.5.1 DDI AUC, C_{max} and C_{trough} ratio goodness-of-fit plots 44
4.5.2 Geometric mean fold error of predicted DDI AUC, C_{max} and C_{trough} ratios . . 45

5 Rifampicin-verapamil drug-drug interaction (DDI) **46**
5.1 DDI modeling 46
5.2 Rifampicin drug-dependent parameters 46
5.3 Rifampicin-verapamil clinical DDI studies 47
5.4 Profiles 48
5.5 Model evaluation 49
5.5.1 DDI AUC and C_{max} ratio goodness-of-fit plots 49

5.5.2 Geometric mean fold error of predicted DDI AUC and C_{max} ratios 50

6 Cimetidine-verapamil drug-drug interaction (DDI) 51
6.1 DDI modeling 51
6.2 Cimetidine drug-dependent parameters 51
6.3 Cimetidine-verapamil clinical DDI studies 52
6.4 Profiles 53
6.5 Model evaluation 56
6.5.1 DDI AUC and C_{max} ratio goodness-of-fit plots 56
6.5.2 Geometric mean fold error of predicted DDI AUC and C_{max} ratios 57

7 System-dependent parameters 58

References 59

1 Physiologically based pharmacokinetic (PBPK) modeling

1.1 PBPK model building

PBPK model building was started with an extensive literature search to collect physicochemical parameters, information on absorption, distribution, metabolism and excretion (ADME) processes and clinical studies of intravenous and oral administration in single- and multiple-dose regimens. In addition to drug plasma concentration-time profiles, observed data on fraction excreted in urine or feces and tissue concentrations should be integrated whenever available. The data of the clinical studies was digitized and divided into a training dataset for model building and a test dataset for model evaluation. The studies for the training dataset were selected to include intravenous and oral studies covering the whole published dosing range. If multiple studies of the same dose were available, studies with many participants, modern bioanalytical methods and frequent as well as late sampling were chosen for the training dataset. Model input parameters that could not be informed from literature were optimized by fitting the model simulations of all studies assigned to the training dataset simultaneously to their respective observed data.

1.2 Virtual individuals

Virtual mean individuals were generated for each study according to the published demographic information with corresponding age, weight, height, sex and ethnicity. If no information was provided, a default value was substituted (30 years of age, male, European, mean weight and height characteristics from the PK-Sim® population database). Enzymes, transporters and binding partners relevant to the pharmacokinetics of the modeled drugs were incorporated in agreement with current literature, utilizing the PK-Sim® expression database [1] to define their relative expression in the different organs of the body. Details and references on the distribution and localization of the implemented metabolizing enzymes, transport proteins and protein binding partners are provided in Section 7.

1.3 PBPK model evaluation

Model performance was evaluated with multiple methods. First, predicted plasma concentration-time profiles were compared with the data observed in the respective clinical studies. Second, the predicted plasma concentration values of all studies were plotted against their corresponding observed values in goodness-of-fit plots. In addition, model performance was evaluated by comparison of predicted to observed AUC and C_{max} values. All AUC values (predicted and observed) were calculated from the time of drug administration to the time of the last plasma concentration measurement (AUC_{last}).

As quantitative measures of the model performance, the mean relative deviation (MRD) of all predicted plasma concentrations (Equation S1) and the geometric mean fold error (GMFE) of all predicted AUC_{last} and C_{max} values (Equation S2) were calculated. MRD and GMFE values ≤ 2 characterize an adequate model performance.

$$MRD = 10^x \text{ with } x = \sqrt{\frac{1}{k}\sum_{i=1}^{k}(\log_{10} c_{\text{predicted},i} - \log_{10} c_{\text{observed},i})^2} \tag{S1}$$

with $c_{\text{predicted},i}$ = predicted plasma concentration, $c_{\text{observed},i}$ = corresponding observed plasma concentration, k = number of observed values.

$$GMFE = 10^x \text{ with } x = \frac{1}{m}\sum_{i=1}^{m}\left|\log_{10}\left(\frac{\text{predicted PK parameter}_i}{\text{observed PK parameter}_i}\right)\right| \tag{S2}$$

with predicted PK parameter$_i$ = predicted AUC_{last} or C_{max} value, observed PK parameter$_i$ = corresponding observed AUC_{last} or C_{max} value, m = number of studies.

Furthermore, physiological plausibility of the parameter estimates and the results of sensitivity analyses were assessed.

1.4 PBPK model sensitivity analysis

Sensitivity of the final model to single parameters (local sensitivity analysis) was calculated, measured as relative change of AUC_{0-24}. Sensitivity analysis was carried out using a relative perturbation of 1000 % (variation range 10.0, maximum number of 9 steps). Parameters were included into the analysis if they have been optimized, if they are associated with optimized parameters or if they might have a strong impact due to calculation methods used in the model.

Sensitivity to a parameter was calculated as the ratio of the relative change of the simulated AUC to the relative variation of the parameter around its value used in the final model according to Equation S3.

$$S = \frac{\Delta AUC}{AUC} \cdot \frac{p}{\Delta p} \tag{S3}$$

where S = sensitivity of the AUC to the examined model parameter, ΔAUC = change of the AUC, AUC = simulated AUC with the original parameter value, Δp = change of the examined parameter value, p = original parameter value. A sensitivity of +1.0 signifies that a 10 % increase of the examined parameter value causes a 10 % increase of the simulated AUC.

1.5 Mathematical implementation of drug-drug interactions

1.5.1 Competitive inhibition

Competitive inhibitors reversibly bind to the active site of an enzyme or transporter and compete with the substrate for binding. Competitive inhibition can be overcome by high substrate concentrations (concentration-dependency); therefore, the maximum reaction velocity (v_{max}) remains unaffected, while the Michaelis-Menten constant (K_m) is increased ($K_{m,app}$, Equation S4). The reaction velocity (v) during co-administration of substrate and competitive inhibitor is described by Equation S5 [2]:

$$K_{m,app} = K_m \cdot \left(1 + \frac{[I]}{K_i}\right) \tag{S4}$$

$$v = \frac{v_{max} \cdot [S]}{K_{m,app} + [S]} \tag{S5}$$

where $K_{m,app}$ = Michaelis-Menten constant in the presence of inhibitor, K_m = Michaelis-Menten constant, $[I]$ = free inhibitor concentration, K_i = dissociation constant of the inhibitor-enzyme/transporter complex, v = reaction velocity, v_{max} = maximum reaction velocity, $[S]$ = free substrate concentration.

1.5.2 Non-competitive inhibition

Non-competitive inhibitors reversibly bind to a site different from the active site. This reduces the activity of the enzyme or transporter, but does not affect the substrate binding. The inhibitor binds to

the free enzyme or to the enzyme-substrate complex with the same dissociation constant (K_i) and the substrate can still bind to the enzyme-inhibitor complex. In the case of non-competitive inhibition, the maximum reaction velocity (v_{max}) is reduced ($v_{max,app}$, Equation S6), while the Michaelis-Menten constant (K_m) remains unaffected. The reaction velocity (v) during co-administration of substrate and non-competitive inhibitor is described by Equation S7 [2]:

$$v_{max,app} = \frac{v_{max}}{1 + \frac{[I]}{K_i}} \tag{S6}$$

$$v = \frac{v_{max,app} \cdot [S]}{K_m + [S]} \tag{S7}$$

where $v_{max,app}$ = maximum reaction velocity in the presence of inhibitor, v_{max} = maximum reaction velocity, $[I]$ = free inhibitor concentration, K_i = dissociation constant of the inhibitor-enzyme/transporter complex and of the inhibitor-enzyme/transporter-substrate complex, v = reaction velocity, $[S]$ = free substrate concentration, K_m = Michaelis-Menten constant.

1.5.3 Mechanism-based inactivation

Mechanism-based inactivation is an irreversible type of inhibition. The return to baseline activity requires the clearance of the mechanism-based inactivator and de novo synthesis of the inactivated protein (time-dependency). In the case of mechanism-based inactivation, the enzyme or transporter degradation rate constant (k_{deg}) is increased ($k_{deg,app}$, Equation S8), while its synthesis rate (R_{syn}) remains unaffected. The enzyme or transporter turnover during administration of mechanism-based inactivator is described by Equation S9. As mechanism-based inactivators are also competitive inhibitors, the K_m in the Michaelis-Menten reaction velocity equation is substituted by $K_{m,app}$ as shown in Equation S10 [2]:

$$k_{deg,app} = k_{deg} + \left(\frac{k_{inact} \cdot [I]}{K_I + [I]} \right) \tag{S8}$$

$$\frac{dE(t)}{dt} = R_{syn} - k_{deg,app} \cdot E(t) \tag{S9}$$

$$v = \frac{v_{max} \cdot [S]}{K_{m,app} + [S]} = \frac{k_{cat} \cdot E(t) \cdot [S]}{K_{m,app} + [S]} \tag{S10}$$

where $k_{deg,app}$ = enzyme or transporter degradation rate constant in the presence of mechanism-based inactivator, k_{deg} = enzyme or transporter degradation rate constant, k_{inact} = maximum inactivation rate constant, $[I]$ = free inactivator concentration, K_I = concentration for half-maximal inactivation, $E(t)$ = enzyme or transporter concentration, R_{syn} = enzyme or transporter synthesis rate, v = reaction velocity, v_{max} = maximum reaction velocity, $[S]$ = free substrate concentration, $K_{m,app}$ = Michaelis-Menten constant in the presence of inactivator, k_{cat} = catalytic rate constant.

1.5.4 Induction

Induction of an enzyme or transporter by rifampicin is mediated by activation of the transcription factor pregnane X receptor (PXR), leading to increased gene expression. The return to baseline activity requires the clearance of the inducer and degradation of the induced protein (time-dependency).

In the case of induction, the enzyme or transporter synthesis rate (R_{syn}) is increased ($R_{syn,app}$, Equation S11), while its degradation rate constant (k_{deg}) remains unaffected. The enzyme or transporter turnover during administration of inducer is described by Equation S12 [2], the reaction velocity is described by Equation S13:

$$R_{syn,app} = R_{syn} \cdot \left(1 + \frac{E_{max} \cdot [Ind]}{EC_{50} + [Ind]}\right) \quad \text{(S11)}$$

$$\frac{dE(t)}{dt} = R_{syn,app} - k_{deg} \cdot E(t) \quad \text{(S12)}$$

$$v = \frac{v_{max} \cdot [S]}{K_m + [S]} = \frac{k_{cat} \cdot E(t) \cdot [S]}{K_m + [S]} \quad \text{(S13)}$$

where $R_{syn,app}$ = enzyme or transporter synthesis rate in the presence of inducer, R_{syn} = enzyme or transporter synthesis rate, E_{max} = maximal induction effect in vivo, $[Ind]$ = free inducer concentration, EC_{50} = concentration for half-maximal induction in vivo, $E(t)$ = enzyme or transporter concentration, k_{deg} = enzyme or transporter degradation rate constant, v = reaction velocity, v_{max} = maximum reaction velocity, $[S]$ = free substrate concentration, K_m = Michaelis-Menten constant, k_{cat} = catalytic rate constant.

2 Verapamil

2.1 PBPK model development

Verapamil is a voltage-dependent calcium channel blocker (class-IV antiarrhythmic agent), used to treat hypertension, angina pectoris and supraventricular tachycardia. It is a BCS Class I drug of high solubility and high permeability, but although $> 90\%$ of an oral dose of verapamil is absorbed, bioavailability is only 10-22% due to high first-pass metabolism [3], with $< 4\%$ excreted unchanged in the urine [4]. Verapamil is administered as a racemic mixture (1:1) of R- and S-verapamil and the enantiomers exhibit different pharmacokinetic and pharmacodynamic properties. The main metabolic pathway for both enantiomers is N-demethylation by CYP3A4, producing R- and S-norverapamil. Verapamil inhibits CYP3A4 and Pgp and it is recommended by the FDA as a moderate clinical CYP3A4 index inhibitor and as a clinical Pgp inhibitor for the use in clinical DDI studies and drug labeling [5].

The verapamil model was established using 45 clinical studies, covering a broad dosing range of 0.1 to 250 mg verapamil, including 7 studies with only one of the verapamil enantiomers (R- or S-verapamil) administered (Table S2.2.1). The final model applies enantioselective plasma protein binding, enantioselective metabolism by CYP3A4 to different metabolites, non-stereospecific transport by Pgp (according to literatur [6–8]) and passive glomerular filtration. To describe the verapamil auto-inhibition and DDI potential, mechanism-based inactivation of CYP3A4 and non-competitive inhibition of Pgp by the verapamil and norverapamil enantiomers were incorporated, based on in vitro reports (Tables S2.3.1 and S2.3.2). Details on the implementation of CYP3A4 and Pgp in the different organs are provided in the system-dependent parameter table (Table S7.0.1)).

The good descriptive and predictive performance of the verapamil model is demonstrated in semilogarithmic (Figure S2.4.1) as well as linear plots (Figure S2.4.2) of predicted compared to observed plasma concentration-time profiles of all clinical studies. Predicted compared to observed fraction excreted in urine data are shown in Figure S2.4.3. Goodness-of-fit plots comparing all predicted to their corresponding observed plasma concentrations are presented (Figure S2.5.1) and MRD values for each study are given (Table S2.5.1). Furthermore, the correlation of predicted to observed AUC and C_{max} values is shown in Figure S2.5.2 and Table S2.5.2 lists the corresponding predicted and observed AUC and C_{max} values of all clinical studies including calculated GMFE values.

Sensitivity analysis of a simulation of 120 mg orally administered racemic verapamil revealed that the predicted total verapamil plasma concentrations are sensitive to the values of fraction unbound of R-verapamil and S-verapamil (both fixed to literature values), and that the predicted total norverapamil plasma concentrations are sensitive to the values of fraction unbound of R-norverapamil and S-norverapamil (both fixed to literature values) as well as to the CYP3A4 catalytic rate constant for the metabolism of R-norverapamil (optimized) (see Figures S2.5.3 and S2.5.4).

2.2 Verapamil clinical studies

The clinical studies used for verapamil model development and evaluation are summarized in Table S2.2.1.

Table S2.2.1: Verapamil study table

Dose [mg]	Route	n	Men [%]	Age [years]	Weight [kg]	Height [cm]	BMI [kg/m^2]	Dataset	Reference
5.0 [a]	iv, 5 min	1	100	(23-27)	92	-	-	training	Eichelbaum et al. 1984 [9]
25.0 [a]	iv, 5 min	1	100	(23-27)	92	-	-	training	Eichelbaum et al. 1984 [9]
50.0 [a]	iv, 5 min	1	100	(23-27)	92	-	-	training	Eichelbaum et al. 1984 [9]
5.0 [b]	iv, 5 min	1	100	(23-27)	92	-	-	training	Eichelbaum et al. 1984 [9]
7.5 [b]	iv, 5 min	1	100	(23-27)	92	-	-	training	Eichelbaum et al. 1984 [9]
10.0 [b]	iv, 5 min	1	100	(23-27)	92	-	-	training	Eichelbaum et al. 1984 [9]
3.0	iv, 5 min	5	64	(25-50)	-	-	-	test	Mooy et al. 1985 [10]
5.0	iv, 10 min	10	100	26 ± 4 (20-34)	74 ± 9 (61-98)	-	23 ± 2 (21-28)	training	Streit et al. 2005 [11]
0.1 /kg	iv, 5 min	6	67	(21-31)	65 ± 6 (60-73)	-	-	test	Johnston et al. 1981 [12]
10.0	iv, 10 min	1	100	24	67	-	-	test	Abernethy et al. 1985 [13]
10.0	iv, 10 min	6	67	(24-37)	-	-	-	training	Barbarash et al. 1988 [14]
10.0	iv, 10 min	1	100	21	70	-	-	test	Wing et al. 1985 [15]
10.0	iv, 5 min	20	100	25 ± 4 (21-34)	-	-	-	test	McAllister, Kirsten 1982 [16]
10.0	iv, bolus	8	100	27 ± 5 (24-38)	-	-	-	test	Smith et al. 1984 [17]
13.1	iv, 13 min	1	100	40	72	-	-	test	Freedman et al. 1981 [18]
20.0	iv, 30 min	1	100	20	79 ± 3	-	-	training	Abernethy et al. 1993 [19]
250 [a]	po, sol	1	100	(23-27)	(68-91)	-	-	test	Vogelgesang et al. 1984 [20]
0.1	po, sol	8	100	26 ± 3	64	-	21 ± 1	training	Maeda et al. 2011 [21]
3.0	po, sol	8	100	26 ± 3	64	-	21 ± 1	training	Maeda et al. 2011 [21]
16.0	po, sol	8	100	26 ± 3	64	-	21 ± 1	training	Maeda et al. 2011 [21]
40.0	po, drag	12	8	27 ± 12 (19-62)	65 ± 10 (50-85)	168 ± 7 (153-178)	23 ± 2 (20-27)	test	Blume, Mutschler 1983 [22]
40.0	po, tab	24	100	25 ± 3 (19-33)	74 ± 8 (62-93)	180 ± 6 (170-193)	23 ± 2 (20-26)	test	Blume, Mutschler 1990 [22]
40.0	po, tab	6	100	26 (22-29)	-	-	-	test	John et al. 1992 [23]
40.0	po, tab	12	50	26 ± 6 (20-38)	71 ± 14 (50-94)	172 ± 16 (155-185)	-	test	Sawicki, Janicki 2002 [24]
60.0	po, caps	12	100	24 ± 1 (22-27)	66 ± 3 (61-70)	172 ± 4 (164-178)	22 ± 2 (20-25)	test	Choi et al. 2008 [25]
80.0	po, -	6	64	(25-50)	-	-	-	test	Mooy et al. 1985 [10]
80.0	po, -	1	100	21	70	-	-	test	Wing et al. 1985 [15]
80.0	po, sol	8	100	26 ± 3	64	-	21 ± 1	test	Maeda et al. 2011 [21]

[a] R-verapamil only, [b] S-verapamil only, [c] as two 40 mg tablets, [d] one hour after verapamil, the 0.25 mg digoxin, 1.0 mg furosemide, 10 mg metformin and 10 mg rosuvastatin transporter probe drug cocktail was administered, **-**: not given, **bid**: twice daily, **BMI**: body mass index, **caps**: capsule, **drag**: dragée, **fed**: administration with a meal, **IR**: Isoptin film-coated tablet, **iv**: intravenous, **n**: number of individuals studied, **po**: oral, **qd**: once daily, **SR**: Isoptin RR retard formulation, **sol**: solution, **tab**: tablet, **test**: test dataset (model evaluation), **tid**: three times daily, **training**: training dataset (model development and parameter optimization)

Table S2.2.1: Verapamil study table *(continued)*

Dose [mg]	Route	n	Men [%]	Age [years]	Weight [kg]	Height [cm]	BMI [kg/m^2]	Dataset	Reference
80.0	po, tab	18	56	26 ± 3 (23-33)	70 ± 10 (55-92)	179 ± 7 (164-191)	22 ± 2 (19-26)	training	Blume, Mutschler 1989 [22]
80.0	po, tab	16	100	31 ± 7 (21-49)	77 ± 10 (60-100)	177 ± 8 (165-197)	25 ± 2 (20-29)	training	Ratiopharm 1988 [26]
80.0 [c]	po, tab	16	100	26 ± 5 (18-32)	74 ± 8 (63-92)	179 ± 7 (170-193)	23 ± 2 (19-27)	test	Ratiopharm 1989 [26]
80.0, tid	po, caps	12	100	(19-38)	-	-	-	training	Johnson et al. 2001 [27]
120.0	po, -	6	67	(24-37)	-	-	-	test	Barbarash et al. 1988 [14]
120.0	po, -	8	100	27 ± 5 (24-38)	-	-	-	test	Smith et al. 1984 [17]
120.0 [d]	po, IR	12	100	39 ± 12 (25-53)	86 ± 10 (72-104)	181 ± 6 (170-191)	26 ± 2 (22-29)	test	Boehringer 2018 [28]
120.0	po, IR	19	58	41 ± 10 (19-55)	73 ± 13 (55-99)	174 ± 10 (154-190)	24 ± 3 (20-29)	training	Härtter et al. 2012 [29]
120.0	po, tab	1	100	24	67	-	-	test	Abernethy et al. 1985 [13]
120.0	po, tab	12	75	28 ± 5 (20-36)	66 ± 15 (41-100)	175 ± 12 (150-190)	21 ± 3 (18-28)	test	Blume, Mutschler 1987 [22]
120.0	po, tab	10	50	(20-48)	-	-	-	training	Hla et al. 1987 [30]
120.0	po, tab	6	67	(21-31)	65 ± 6 (60-73)	-	-	test	Johnston et al.1981 [12]
120.0, bid	po, IR	20	60	38 ± 11 (20-55)	73 ± 10 (58-92)	175 ± 9 (160-200)	24 ± 3 (20-29)	training	Härtter et al. 2012 [29]
120.0, bid	po, tab	10	50	(20-48)	-	-	-	training	Hla et al. 1987 [30]
160.0	po, sol	1	100	(25-43)	(66-87)	-	-	test	Mikus et al. 1990 [31]
180.0, bid	po, - , fed	10	60	(19-32)	(62-85)	(165-193)	-	test	van Haarst et al. 2009 [32]
240.0, qd	po, SR	24	100	31 ± 9 (20-47)	76 ± 8 (58-88)	179 ± 6 (170-193)	24 ± 2 (19-27)	test	Blume, Mutschler 1994 [22]

[a] R-verapamil only, [b] S-verapamil only, [c] as two 40 mg tablets, [d] one hour after verapamil, the 0.25 mg digoxin, 1.0 mg furosemide, 10 mg metformin and 10 mg rosuvastatin transporter probe drug cocktail was administered, **-**: not given, **bid**: twice daily, **BMI**: body mass index, **caps**: capsule, **drag**: dragée, **fed**: administration with a meal, **IR**: Isoptin film-coated tablet, **iv**: intravenous, **n**: number of individuals studied, **po**: oral, **qd**: once daily, **SR**: Isoptin RR retard formulation, **sol**: solution, **tab**: tablet, **test**: test dataset (model evaluation), **tid**: three times daily, **training**: training dataset (model development and parameter optimization)

2.3 Verapamil and norverapamil drug-dependent parameters

The drug-dependent parameters of the final verapamil parent-metabolite model are summarized in Table S2.3.1 (R-verapamil and S-verapamil parameter values) and Table S2.3.2 (R-norverapamil and S-norverapamil parameter values) below. The associated system-dependent parameters are listed in Table S7.0.1.

Table S2.3.1: R- and S-verapamil drug-dependent parameters

Parameter	Value	Unit	Source	Literature	Reference	Value	Unit	Source	Literature	Reference	Description
	**R-Verapamil**					**S-Verapamil**					
MW	454.611	g/mol	Literature	454.611	[33]	454.611	g/mol	Literature	454.611	[33]	Molecular weight
pKa (base)	8.75	-	Literature	8.75	[34]	8.75	-	Literature	8.75	[34]	Acid dissociation constant
Solubility (pH 6.54)	46.0	g/l	Literature	46.0	[35]	46.0	g/l	Literature	46.0	[35]	Solubility
logP	2.84 *	-	Optimized	3.79	[36]	2.84 *	-	Optimized	3.79	[36]	Lipophilicity
fu	5.1	%	Literature	5.1	[37]	11.0	%	Literature	11.0	[37]	Fraction unbound
CYP3A4 K_m -> Norvera	19.59	µmol/l	Literature	19.59 ‡	[38]	9.72	µmol/l	Literature	9.72 ‡	[38]	CYP3A4 Michaelis-Menten constant
CYP3A4 k_{cat} -> Norvera	34.94	1/min	Optimized	-	-	26.17	1/min	Optimized	-	-	CYP3A4 catalytic rate constant
CYP3A4 K_m -> D617	35.34	µmol/l	Literature	35.34 ‡	[38]	23.64	µmol/l	Literature	23.64 ‡	[38]	CYP3A4 Michaelis-Menten constant
CYP3A4 k_{cat} -> D617	43.98	1/min	Optimized	-	-	56.42	1/min	Optimized	-	-	CYP3A4 catalytic rate constant
Pgp K_m	1.01	µmol/l	Literature	1.01	[39]	1.01	µmol/l	Literature	1.01	[39]	Pgp Michaelis-Menten constant
Pgp k_{cat}	12.60 °	1/min	Optimized	-	-	12.60 °	1/min	Optimized	-	-	Pgp transport rate constant
GFR fraction	1.00	-	Assumed	-	-	1.00	-	Assumed	-	-	Fraction of filtered drug in the urine
EHC continuous fraction	1.00	-	Assumed	-	-	1.00	-	Assumed	-	-	Fraction of bile continually released
CYP3A4 MBI K_I	27.63	µmol/l	Literature	27.63 ‡	[38]	3.85	µmol/l	Literature	3.85 ‡	[38]	Conc. for half-maximal inactivation
CYP3A4 MBI k_{inact}	0.038	1/min	Literature	0.038	[38]	0.034	1/min	Literature	0.034	[38]	Maximum inactivation rate
Pgp non-competitive K_i	0.038 *	µmol/l	Optimized	0.31	[40]	0.038 *	µmol/l	Optimized	0.31	[40]	Conc. for half-maximal inhibition
Partition coefficients	Diverse	-	Calculated	R&R	[41, 42]	Diverse	-	Calculated	R&R	[41, 42]	Cell to plasma partition coefficients
Cellular permeability	9.94E-02 *	cm/min	Optimized	PK-Sim	[2]	9.94E-02 *	cm/min	Optimized	PK-Sim	[2]	Permeability into the cellular space
Intestinal permeability	3.54E-06 *	cm/min	Optimized	1.21E-05	Calculated	3.54E-06 *	cm/min	Optimized	1.21E-05	Calculated	Transcellular intestinal permeability
SR tablet Weibull time	155.24	min	Optimized	-	[22]	155.24	min	Optimized	-	[22]	Dissolution time (50% dissolved)
SR tablet Weibull shape	2.37	-	Optimized	-	[22]	2.37	-	Optimized	-	[22]	Dissolution profile shape

* assumed to be the same for all four compounds, ° assumed to be the same for R-/S-verapamil, ‡ in vitro values corrected for binding in the assay using fraction unbound to microsomal protein measurements from the same study, **conc**: concentration, **CYP3A4**: cytochrome P450 3A4, **D617**: verapamil metabolite, **EHC**: enterohepatic circulation, **GFR**: glomerular filtration rate, **MBI**: mechanism-based inactivation, **Norvera**: norverapamil, **Pgp**: P-glycoprotein, **PK-Sim**: PK-Sim standard calculation method, **R&R**: Rodgers and Rowland calculation method, **SR**: sustained release formulation

Table S2.3.2: R- and S-norverapamil drug-dependent parameters

Parameter	Value	Unit	Source	Literature	Reference	Value	Unit	Source	Literature	Reference	Description
	**R-Norverapamil**					**S-Norverapamil**					
MW	440.584	g/mol	Literature	440.584	[33]	440.584	g/mol	Literature	440.584	[33]	Molecular weight
pKa (base)	8.75	-	Literature	8.6 - 8.9	[43]	8.75	-	Literature	8.6 - 8.9	[43]	Acid dissociation constant
logP	2.84 *	-	Optimized	-	-	2.84 *	-	Optimized	-	-	Lipophilicity
fu	5.1 [a]	%	Assumed	-	-	11.0 [b]	%	Assumed	-	-	Fraction unbound
CYP3A4 K_m -> D620	144.0	µmol/l	Literature	144.0	[44]	36.0	µmol/l	Literature	36.0	[44]	CYP3A4 Michaelis-Menten constant
CYP3A4 k_{cat} -> D620	145.64	1/min	Optimized	-	-	41.10	1/min	Optimized	-	-	CYP3A4 catalytic rate constant
Pgp K_m	1.01 *	µmol/l	Assumed	-	-	1.01 *	µmol/l	Assumed	-	-	Pgp Michaelis-Menten constant
Pgp k_{cat}	3.39 °	1/min	Optimized	-	-	3.39 °	1/min	Optimized	-	-	Pgp transport rate constant
GFR fraction	1.00	-	Assumed	-	-	1.00	-	Assumed	-	-	Fraction of filtered drug in the urine
EHC continuous fraction	1.00	-	Assumed	-	-	1.00	-	Assumed	-	-	Fraction of bile continually released
CYP3A4 MBI K_I	6.10	µmol/l	Literature	6.10 ‡	[38]	2.90	µmol/l	Literature	2.90 ‡	[38]	Conc. for half-maximal inactivation
CYP3A4 MBI k_{inact}	0.048	1/min	Literature	0.048	[38]	0.080	1/min	Literature	0.080	[38]	Maximum inactivation rate
Pgp non-competitive K_i	0.038 *	µmol/l	Optimized	0.30 [c]	[45]	0.038 *	µmol/l	Optimized	0.30 [c]	[45]	Conc. for half-maximal inhibition
Partition coefficients	Diverse	-	Calculated	R&R	[41, 42]	Diverse	-	Calculated	R&R	[41, 42]	Cell to plasma partition coefficients
Cellular permeability	9.94E-02 *	cm/min	Optimized	PK-Sim	[2]	9.94E-02 *	cm/min	Optimized	PK-Sim	[2]	Permeability into the cellular space
Intestinal permeability	3.54E-06 *	cm/min	Optimized	1.40E-05	Calculated	3.54E-06 *	cm/min	Optimized	1.40E-05	Calculated	Transcellular intestinal permeability

* assumed to be the same for all four compounds, ° assumed to be the same for R-/S-norverapamil, ‡ in vitro values corrected for binding in the assay using fraction unbound to microsomal protein measurements from the same study, [a] assumed to be the same for R-verapamil/R-norverapamil, [b] assumed to be the same for S-verapamil/S-norverapamil, [c] IC_{50} at very low substrate concentration, **conc**: concentration, **CYP3A4**: cytochrome P450 3A4, **D620**: norverapamil metabolite, **EHC**: enterohepatic circulation, **GFR**: glomerular filtration rate, **MBI**: mechanism-based inactivation, **Pgp**: P-glycoprotein, **PK-Sim**: PK-Sim standard calculation method, **R&R**: Rodgers and Rowland calculation method

2.4 Profiles

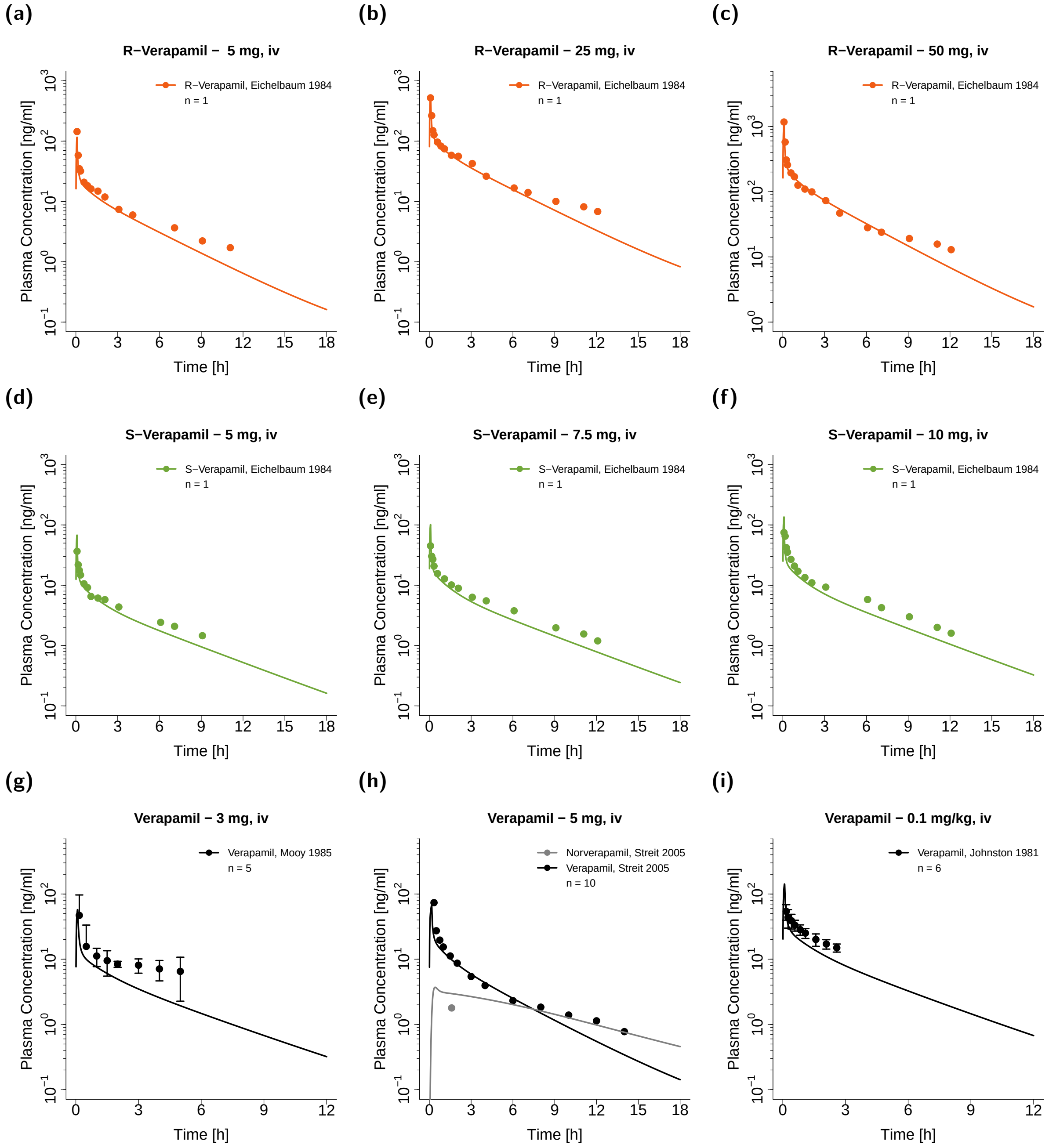

Figure S2.4.1: Verapamil and norverapamil plasma concentration-time profiles (semilogarithmic) following intravenous or oral administration of verapamil. Observed data are shown as dots, if available ± standard deviation (SD). Simulations are shown as lines.

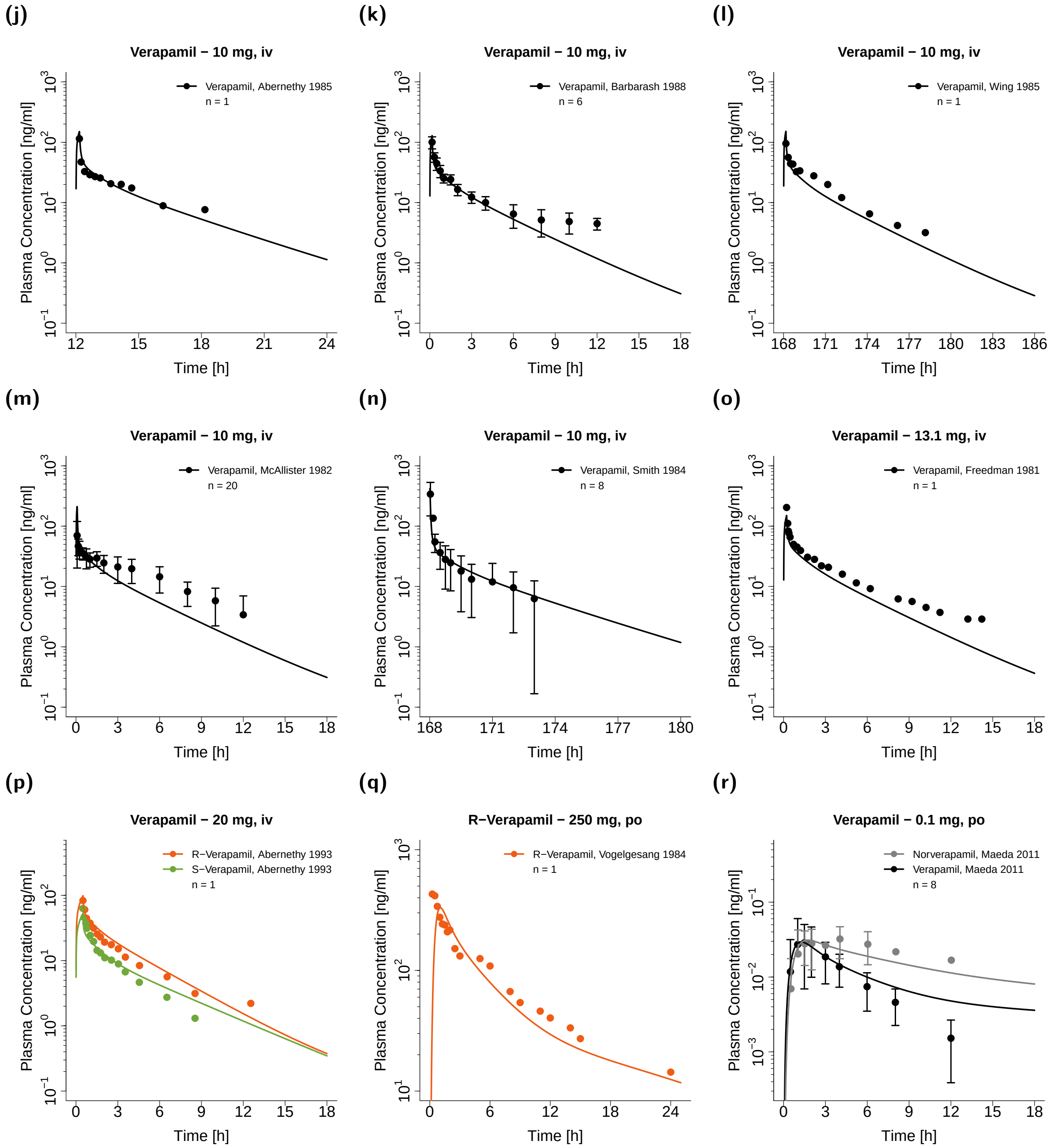

Figure S2.4.1: Verapamil and norverapamil plasma concentration-time profiles (semilogarithmic) following intravenous or oral administration of verapamil. Observed data are shown as dots, if available ± standard deviation (SD). Simulations are shown as lines. (continued)

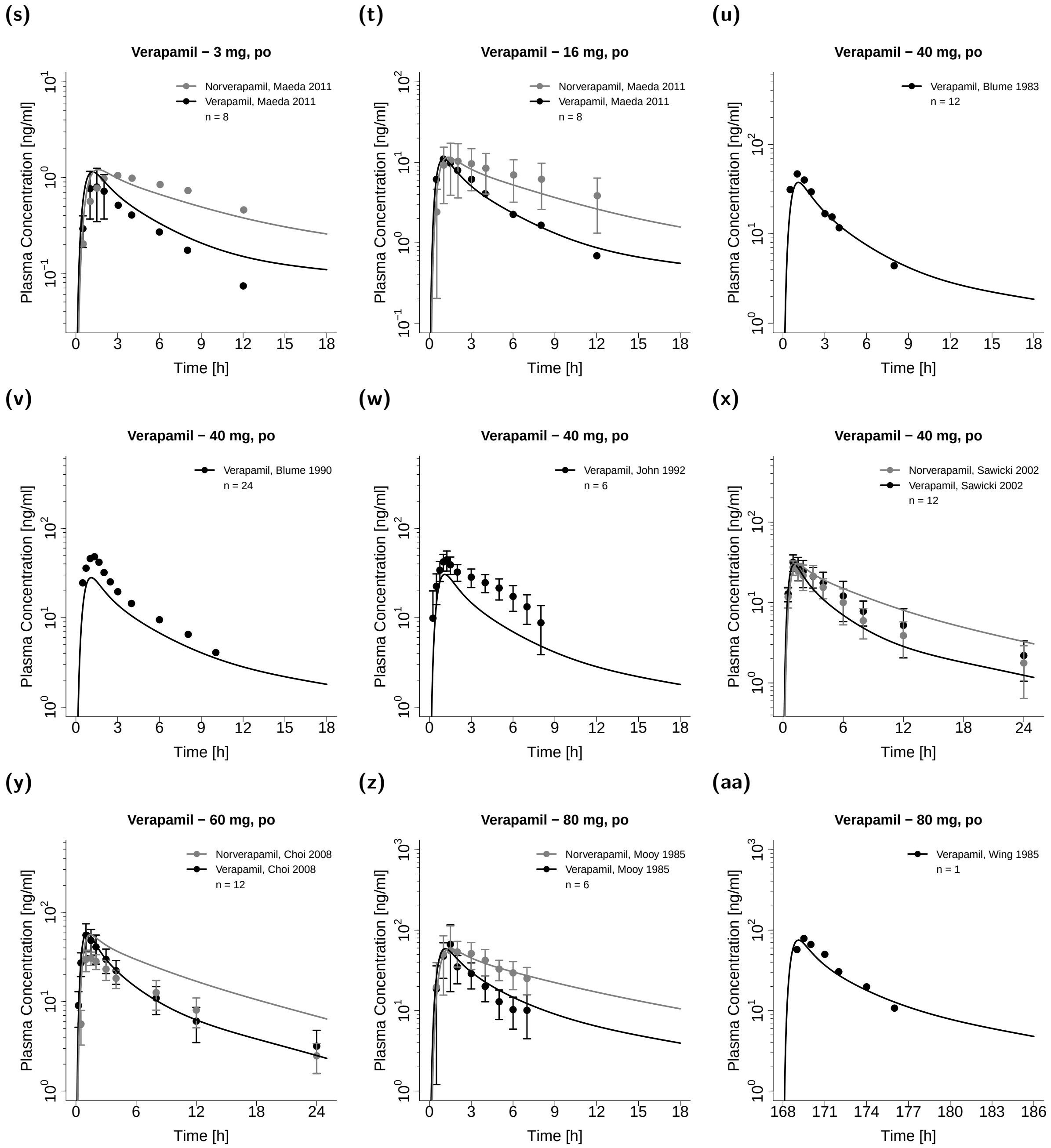

Figure S2.4.1: Verapamil and norverapamil plasma concentration-time profiles (semilogarithmic) following intravenous or oral administration of verapamil. Observed data are shown as dots, if available ± standard deviation (SD). Simulations are shown as lines. (continued)

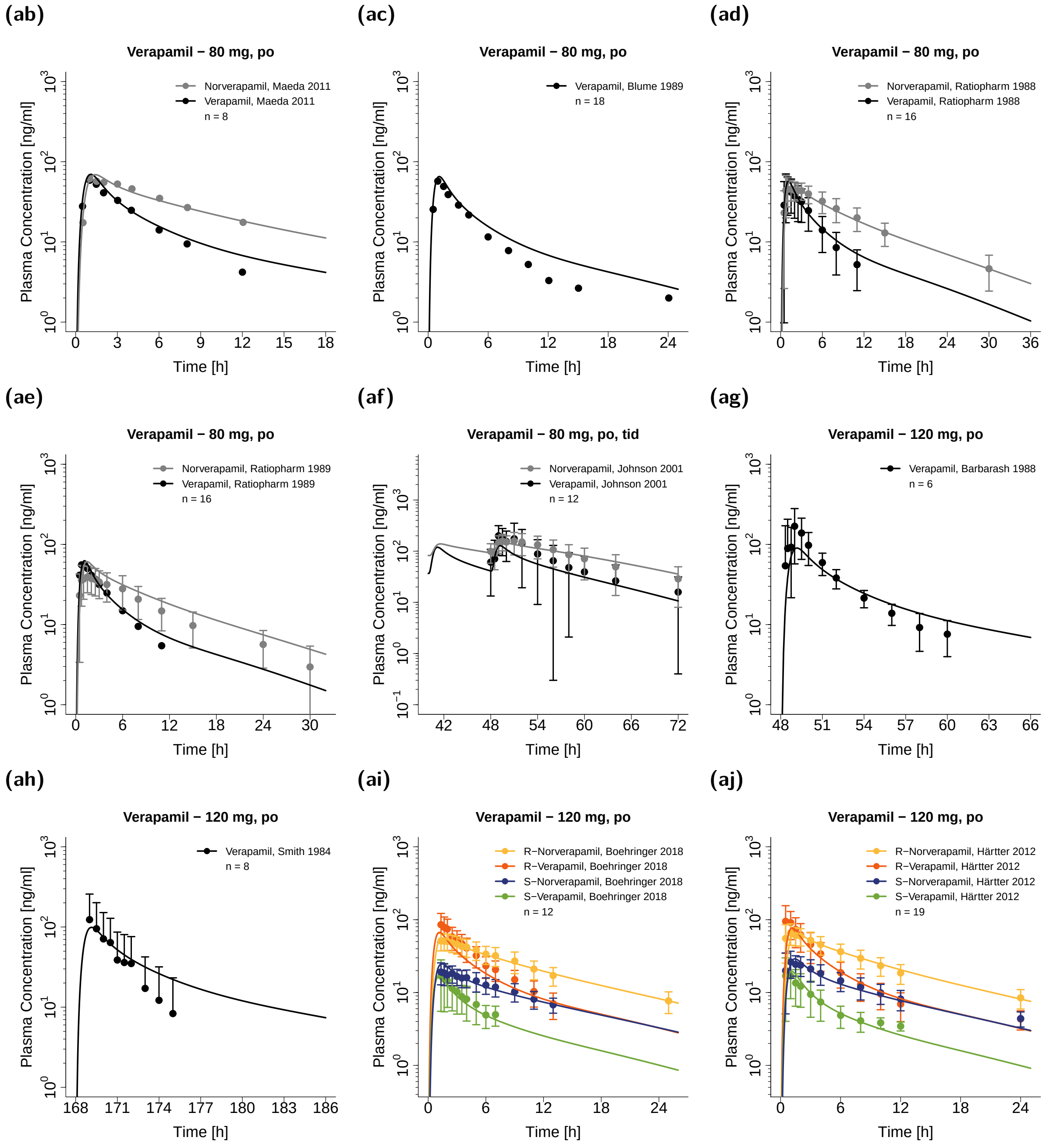

Figure S2.4.1: Verapamil and norverapamil plasma concentration-time profiles (semilogarithmic) following intravenous or oral administration of verapamil. Observed data are shown as dots, if available ± standard deviation (SD). Simulations are shown as lines. (continued)

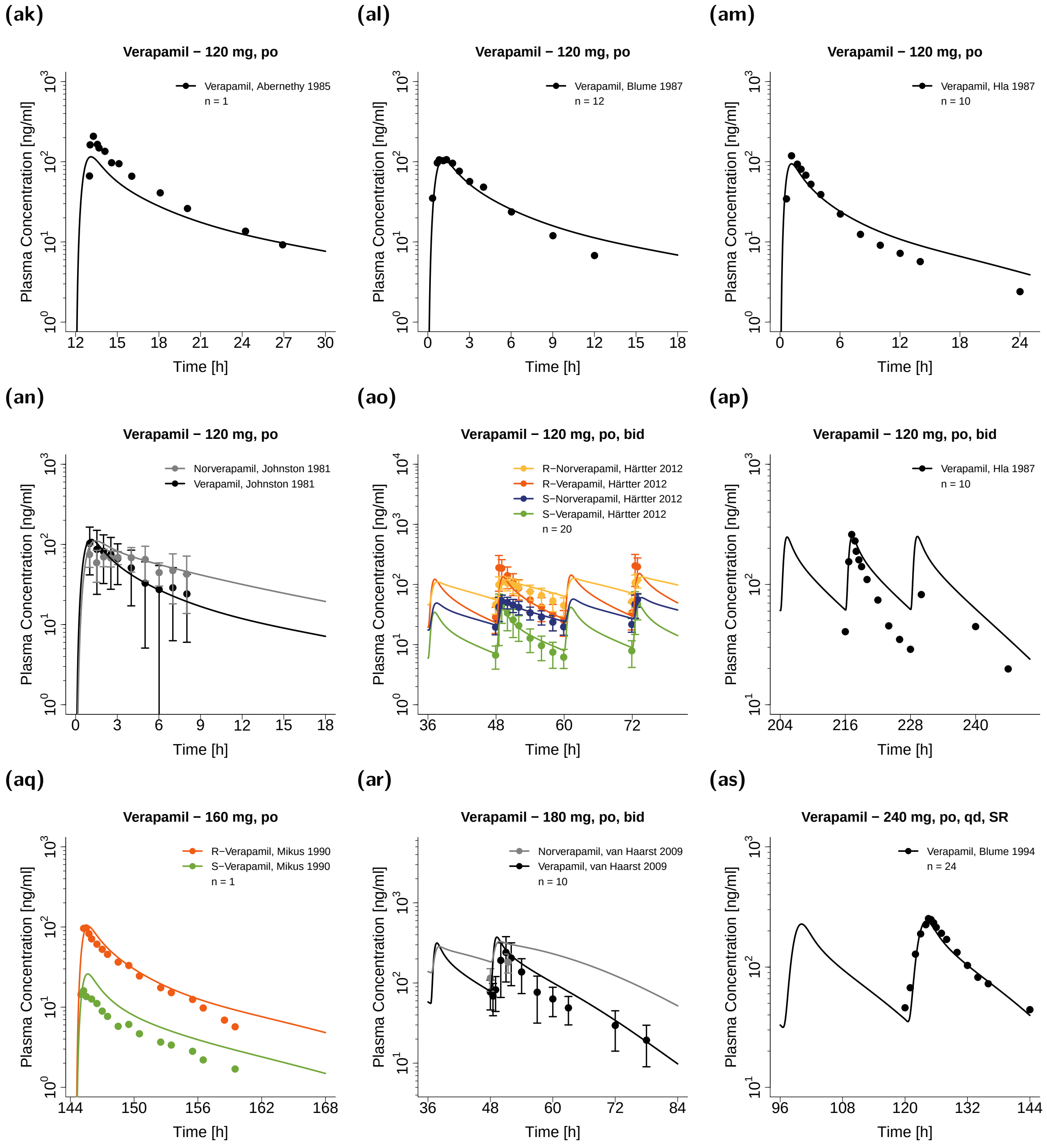

Figure S2.4.1: Verapamil and norverapamil plasma concentration-time profiles (semilogarithmic) following intravenous or oral administration of verapamil. Observed data are shown as dots, if available ± standard deviation (SD). Simulations are shown as lines. (continued)

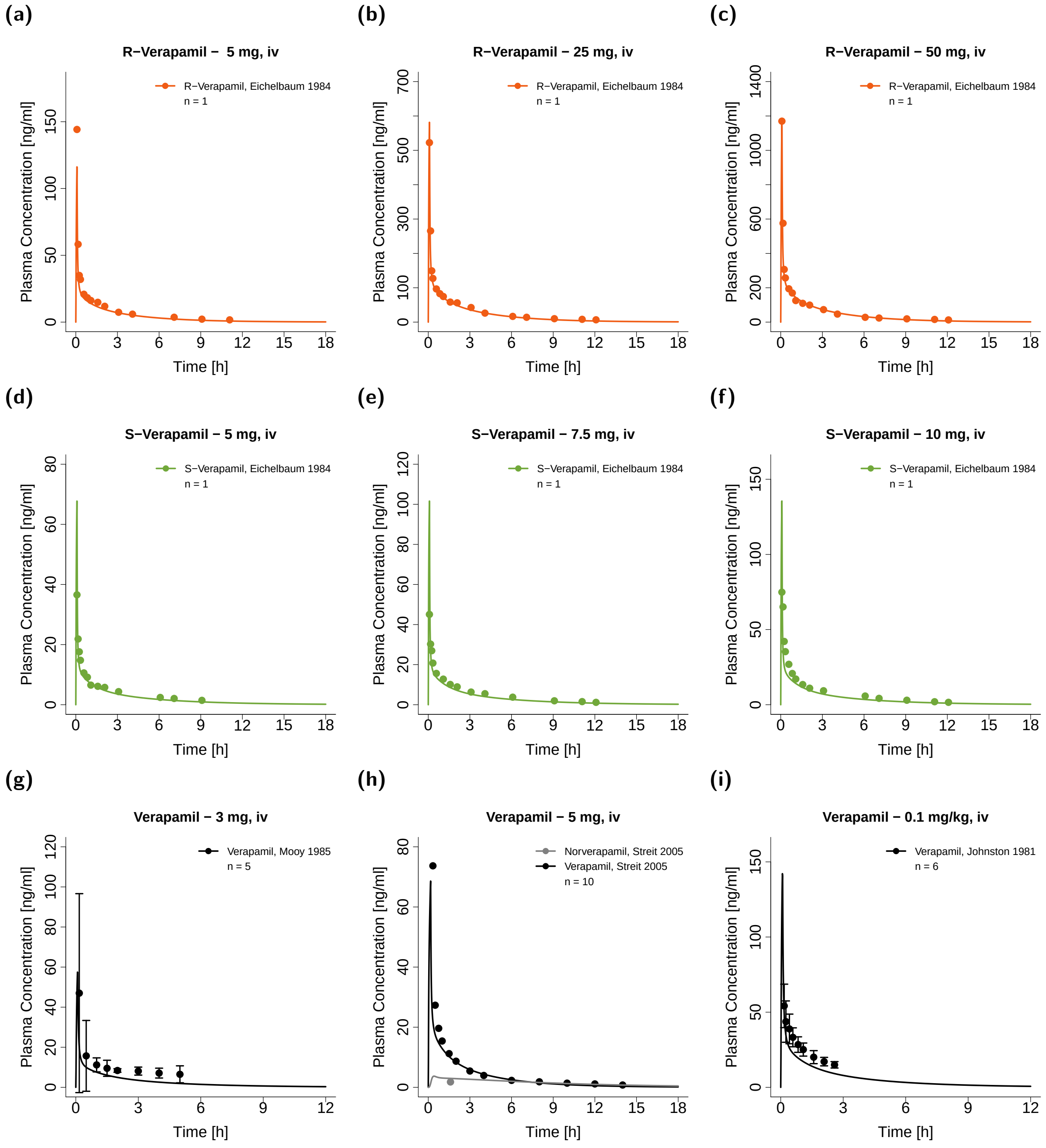

Figure S2.4.2: Verapamil and norverapamil plasma concentration-time profiles (linear) following intravenous or oral administration of verapamil. Observed data are shown as dots, if available ± standard deviation (SD). Simulations are shown as lines.

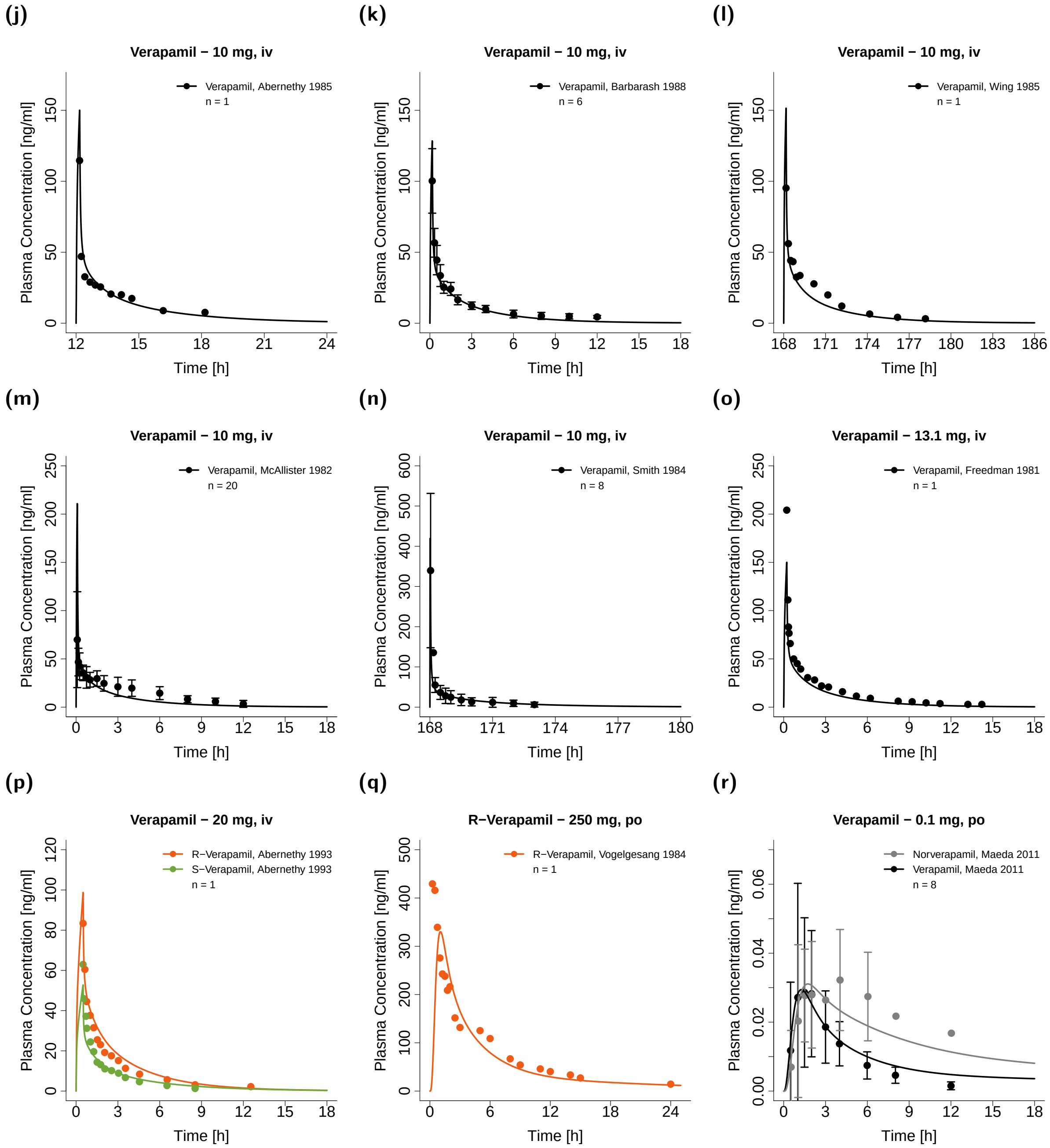

Figure S2.4.2: Verapamil and norverapamil plasma concentration-time profiles (linear) following intravenous or oral administration of verapamil. Observed data are shown as dots, if available ± standard deviation (SD). Simulations are shown as lines. (continued)

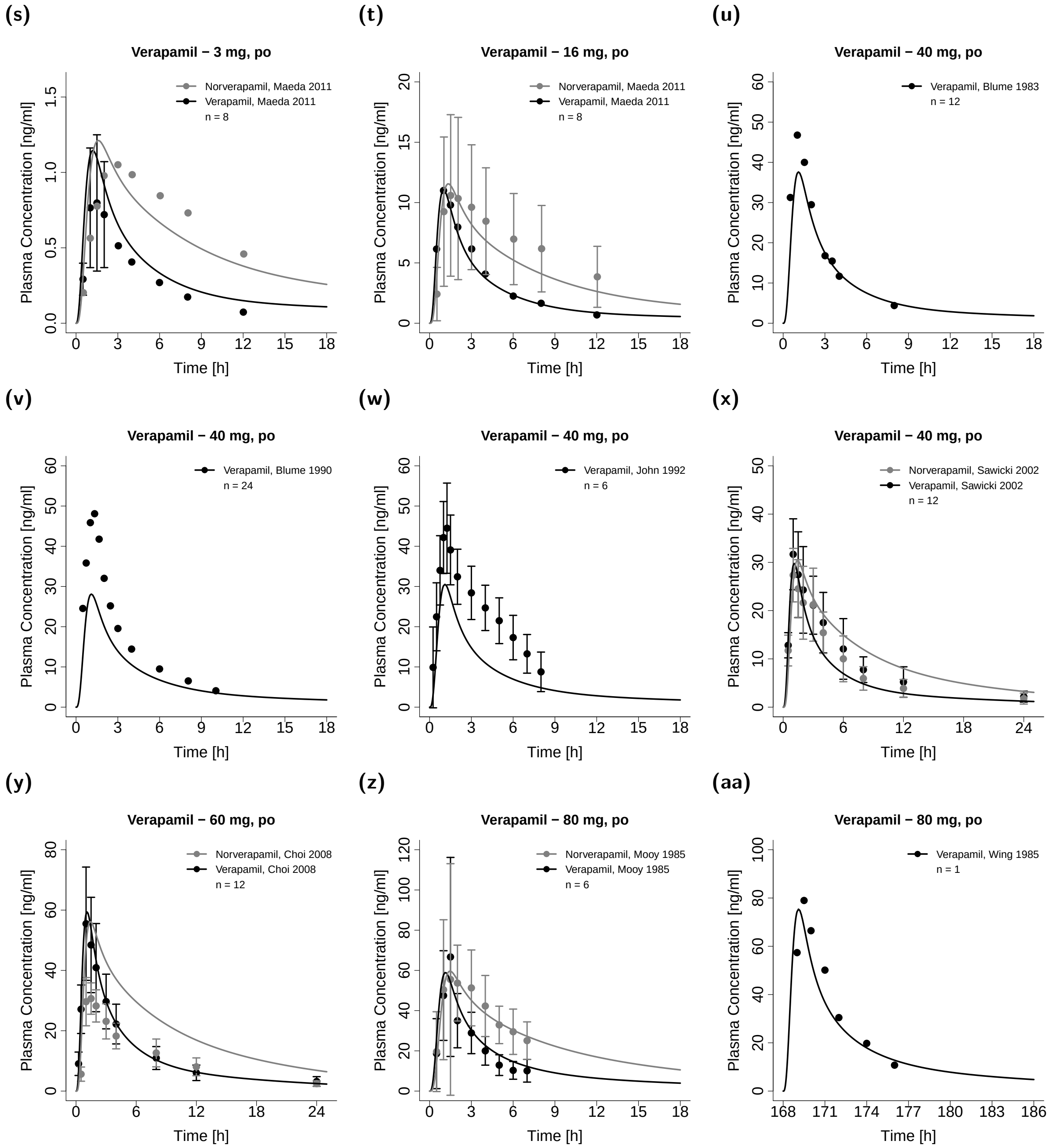

Figure S2.4.2: Verapamil and norverapamil plasma concentration-time profiles (linear) following intravenous or oral administration of verapamil. Observed data are shown as dots, if available ± standard deviation (SD). Simulations are shown as lines. (continued)

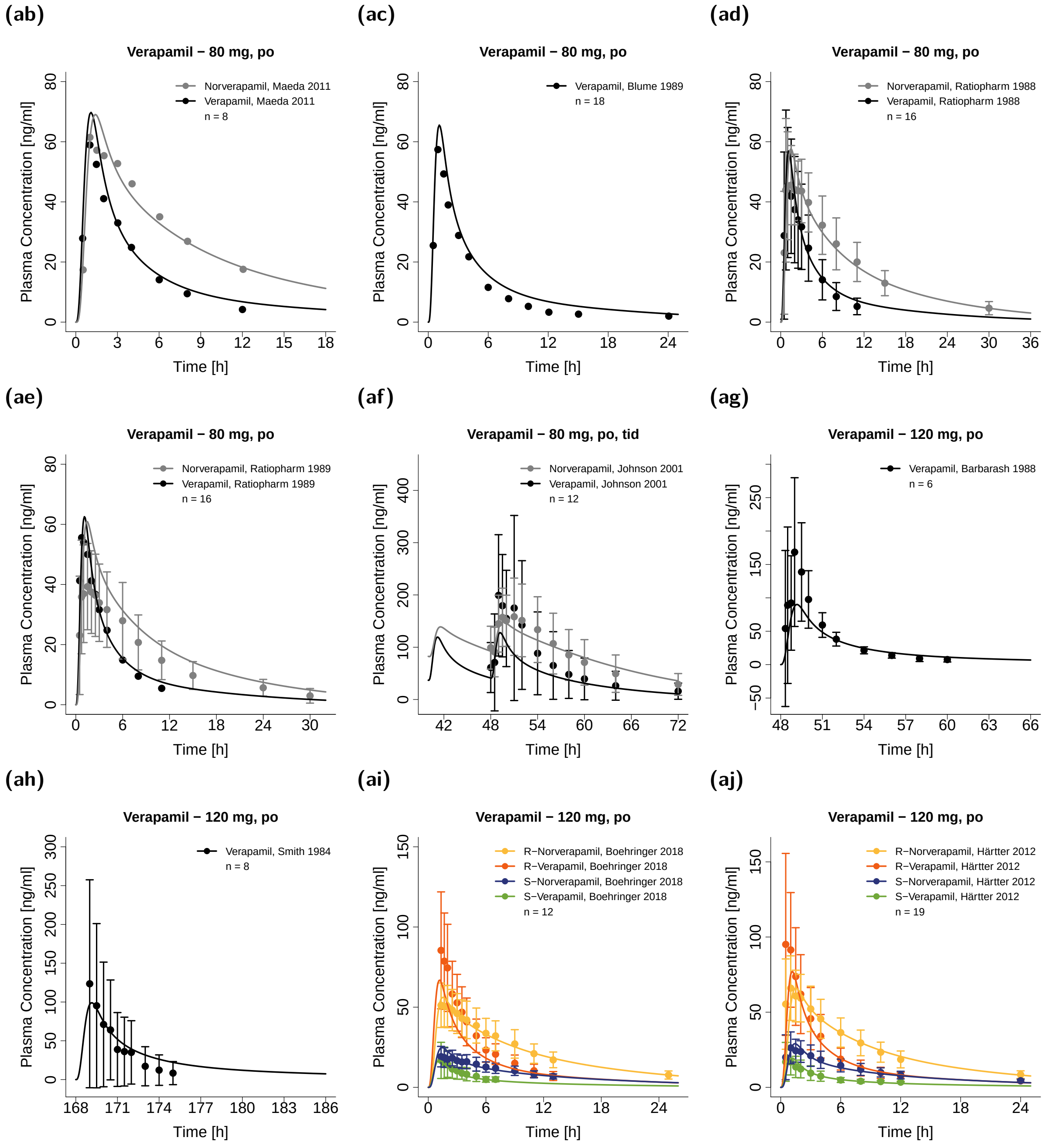

Figure S2.4.2: Verapamil and norverapamil plasma concentration-time profiles (linear) following intravenous or oral administration of verapamil. Observed data are shown as dots, if available ± standard deviation (SD). Simulations are shown as lines. (continued)

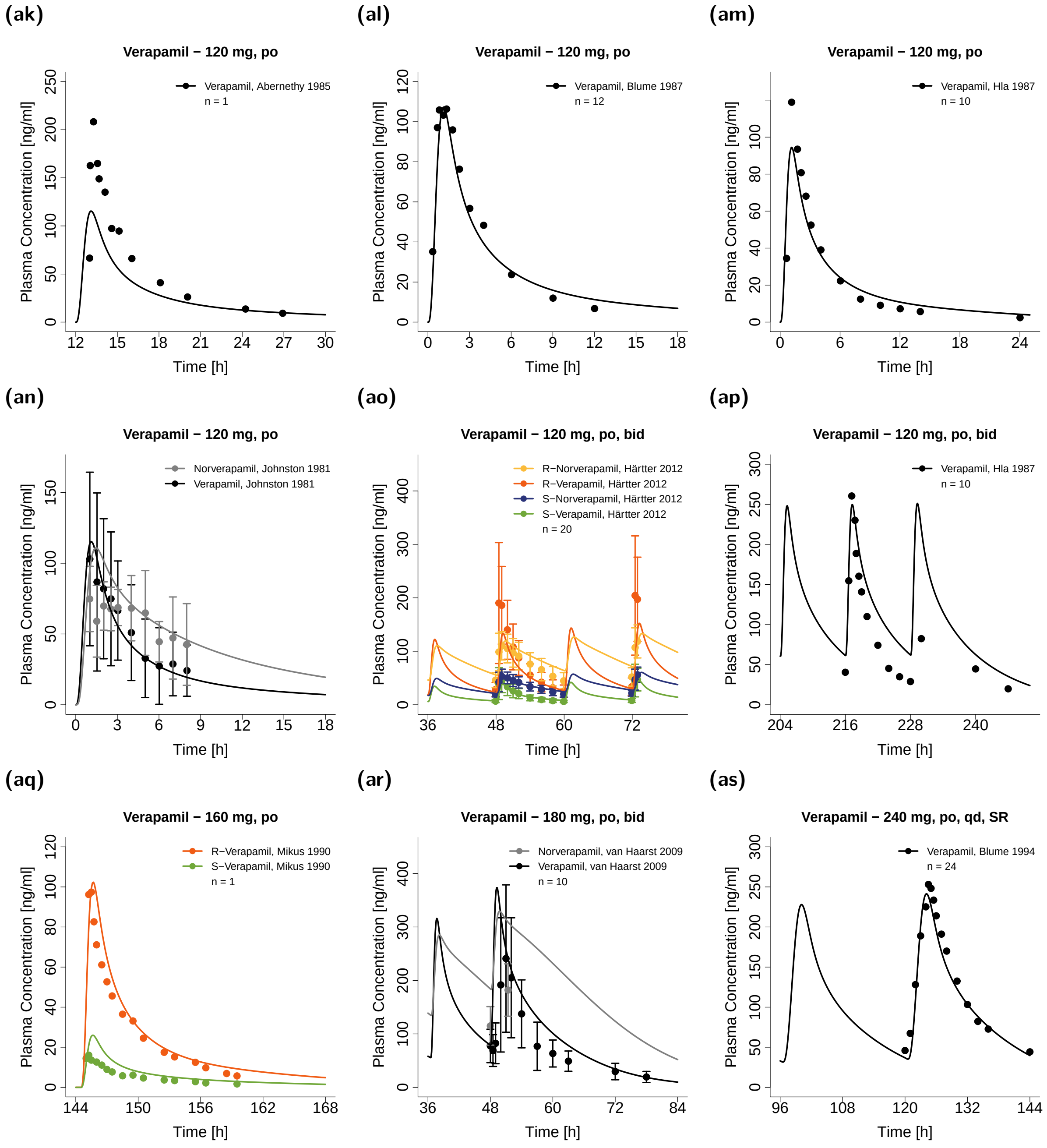

Figure S2.4.2: Verapamil and norverapamil plasma concentration-time profiles (linear) following intravenous or oral administration of verapamil. Observed data are shown as dots, if available ± standard deviation (SD). Simulations are shown as lines. (continued)

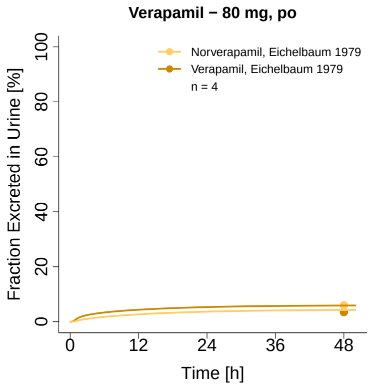

Figure S2.4.3: Verapamil and norverapamil fraction excreted in urine following oral administration of verapamil. Observed data are shown as dots. Simulations are shown as lines.

2.5 Model evaluation

2.5.1 Predicted concentrations versus observed concentrations goodness-of-fit plots

(a) Training

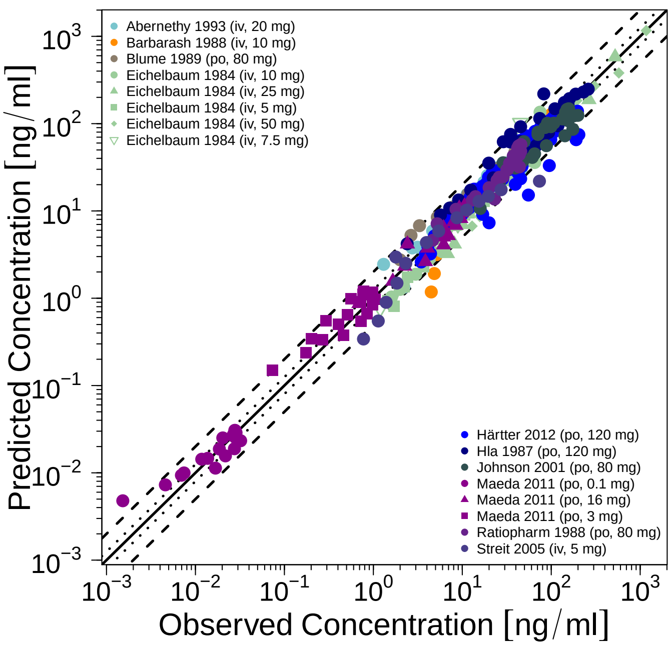

(b) Test

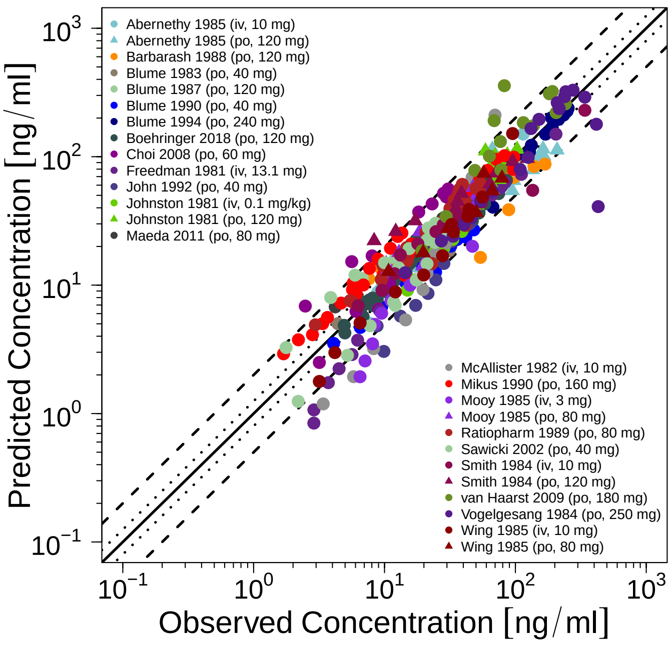

Figure S2.5.1: Predicted versus observed verapamil and norverapamil plasma concentrations of (a) the training and (b) the test dataset. The solid line (——) marks the line of identity. Dotted lines (·····) indicate 1.25-fold, dashed lines (- -) indicate 2-fold deviation.

2.5.2 Mean relative deviation of plasma concentration predictions

Table S2.5.1: Mean relative deviation (MRD) values of verapamil and norverapamil plasma concentration predictions

Route	Compound	Dose	MRD	Reference
**Intravenous**				
iv, 5 min	R-Verapamil	5.00 mg	1.41	Eichelbaum et al. 1984 [9]
iv, 5 min	R-Verapamil	25.00 mg	1.34	Eichelbaum et al. 1984 [9]
iv, 5 min	R-Verapamil	50.00 mg	1.30	Eichelbaum et al. 1984 [9]
iv, 5 min	S-Verapamil	5.00 mg	1.36	Eichelbaum et al. 1984 [9]
iv, 5 min	S-Verapamil	7.50 mg	1.43	Eichelbaum et al. 1984 [9]
iv, 5 min	S-Verapamil	10.00 mg	1.51	Eichelbaum et al. 1984 [9]
iv, 5 min	Verapamil	3.00 mg	2.17	Mooy et al. 1985 [10]
iv, 10 min	Verapamil	5.00 mg	1.65	Streit et al. 2005 [11]
iv, 10 min	Norverapamil	5.00 mg	1.66	Streit et al. 2005 [11]
iv, 5 min	Verapamil	0.10 mg/kg	1.45	Johnston et al. 1981 [12]
iv, 10 min	Verapamil	10.00 mg	1.24	Abernethy et al. 1985 [13]
iv, 10 min	Verapamil	10.00 mg	1.64	Barbarash et al. 1988 [14]
iv, 10 min	Verapamil	10.00 mg	1.42	Wing et al. 1985 [15]
iv, 5 min	Verapamil	10.00 mg	1.99	McAllister, Kirsten 1982 [16]
iv, 5 min	Verapamil	10.00 mg	1.38	Smith et al. 1984 [17]
iv, 13 min	Verapamil	13.10 mg	1.68	Freedman et al. 1981 [18]
iv, 30 min	R-Verapamil	20.00 mg	1.26	Abernethy et al. 1993 [19]
iv, 30 min	S-Verapamil	20.00 mg	1.31	Abernethy et al. 1993 [19]
**MRD**			**1.51 (1.24–2.17)**	
			**17/18 with MRD ≤ 2**	
**Oral**				
po, sol	R-Verapamil	250.00 mg	1.85	Vogelgesang et al. 1984 [20]
po, sol	Verapamil	0.10 mg	1.54	Maeda et al. 2011 [21]
po, sol	Norverapamil	0.10 mg	1.31	Maeda et al. 2011 [21]
po, tab	Verapamil	3.00 mg	1.50	Maeda et al. 2011 [21]
po, tab	Norverapamil	3.00 mg	1.40	Maeda et al. 2011 [21]
po, sol	Verapamil	16.00 mg	1.12	Maeda et al. 2011 [21]
po, sol	Norverapamil	16.00 mg	1.35	Maeda et al. 2011 [21]
po, drag	Verapamil	40.00 mg	1.25	Blume, Mutschler 1983 [22]
po, tab	Verapamil	40.00 mg	1.56	Blume, Mutschler 1990 [22]
po, tab	Verapamil	40.00 mg	1.98	John et al. 1992 [23]
po, tab	Verapamil	40.00 mg	1.49	Sawicki, Janicki 2002 [24]
po, tab	Norverapamil	40.00 mg	1.55	Sawicki, Janicki 2002 [24]
po, caps	Verapamil	60.00 mg	1.15	Choi et al. 2008 [25]
po, caps	Norverapamil	60.00 mg	2.09	Choi et al. 2008 [25]
po, -	Verapamil	80.00 mg	1.28	Mooy et al. 1985 [10]
po, -	Norverapamil	80.00 mg	1.14	Mooy et al. 1985 [10]
po, -	Verapamil	80.00 mg	1.22	Wing et al. 1985 [15]
po, sol	Verapamil	80.00 mg	1.23	Maeda et al. 2011 [21]
po, sol	Norverapamil	80.00 mg	1.11	Maeda et al. 2011 [21]
po, tab	Verapamil	80.00 mg	1.45	Blume, Mutschler 1989 [22]
po, tab	Verapamil	80.00 mg	1.18	Ratiopharm 1988 [26]
po, tab	Norverapamil	80.00 mg	1.22	Ratiopharm 1988 [26]
po, tab	Verapamil	80.00 mg	1.18	Ratiopharm 1989 [26]
po, tab	Norverapamil	80.00 mg	1.39	Ratiopharm 1989 [26]
po, caps	Verapamil	80.00 mg (tid)	1.52	Johnson et al. 2001 [27]
po, caps	Norverapamil	80.00 mg (tid)	1.14	Johnson et al. 2001 [27]
po, -	Verapamil	120.00 mg	1.71	Barbarash et al. 1988 [14]
po, -	Verapamil	120.00 mg	1.59	Smith et al. 1984 [17]
po, IR	R-Verapamil	120.00 mg	1.36	Boehringer 2018 [28]
po, IR	R-Norverapamil	120.00 mg	1.05	Boehringer 2018 [28]
po, IR	S-Verapamil	120.00 mg	1.13	Boehringer 2018 [28]
po, IR	S-Norverapamil	120.00 mg	1.15	Boehringer 2018 [28]
po, IR	R-Verapamil	120.00 mg	1.41	Härtter et al. 2012 [29]
po, IR	R-Norverapamil	120.00 mg	1.49	Härtter et al. 2012 [29]
po, IR	S-Verapamil	120.00 mg	1.28	Härtter et al. 2012 [29]
po, IR	S-Norverapamil	120.00 mg	1.39	Härtter et al. 2012 [29]
po, tab	Verapamil	120.00 mg	1.54	Abernethy et al. 1985 [13]
po, tab	Verapamil	120.00 mg	1.26	Blume, Mutschler 1987 [22]

-: not given, **bid**: twice daily, **caps**: capsule, **drag**: dragée, **fed**: administration with a meal, **IR**: Isoptin film-coated tablet, **iv**: intravenous, **MRD**: mean relative deviation, **po**: oral, **qd**: once daily, **SR**: Isoptin RR retard formulation, **sol**: solution, **tab**: tablet, **tid**: three times daily

Table S2.5.1: Mean relative deviation (MRD) values of verapamil and norverapamil plasma concentration predictions *(continued)*

Route	Compound	Dose	MRD	Reference
po, tab	Verapamil	120.00 mg	1.40	Hla et al. 1987 [30]
po, tab	Verapamil	120.00 mg	1.16	Johnston et al. 1981 [12]
po, tab	Norverapamil	120.00 mg	1.32	Johnston et al. 1981 [12]
po, IR	R-Verapamil	120.00 mg (bid)	1.57	Härtter et al. 2012 [29]
po, IR	R-Norverapamil	120.00 mg (bid)	1.27	Härtter et al. 2012 [29]
po, IR	S-Verapamil	120.00 mg (bid)	1.35	Härtter et al. 2012 [29]
po, IR	S-Norverapamil	120.00 mg (bid)	1.22	Härtter et al. 2012 [29]
po, tab	Verapamil	120.00 mg (bid)	1.64	Hla et al. 1987 [30]
po, sol	R-Verapamil	160.00 mg	1.25	Mikus et al. 1990 [31]
po, sol	S-Verapamil	160.00 mg	1.63	Mikus et al. 1990 [31]
po, -, fed	Verapamil	180.00 mg (bid)	1.82	van Haarst et al. 2009 [32]
po, -, fed	Norverapamil	180.00 mg (bid)	1.65	van Haarst et al. 2009 [32]
po, SR	Verapamil	240.00 mg (qd)	1.18	Blume, Mutschler 1994 [22]
**MRD**			**1.43 (1.05–2.09)**	
			**50/51 with MRD ≤ 2**	
**Overall MRD**			**1.46 (1.05–2.17)**	
			**67/69 with MRD ≤ 2**	

-: not given, **bid**: twice daily, **caps**: capsule, **drag**: dragée, **fed**: administration with a meal, **IR**: Isoptin film-coated tablet, **iv**: intravenous, **MRD**: mean relative deviation, **po**: oral, **qd**: once daily, **SR**: Isoptin RR retard formulation, **sol**: solution, **tab**: tablet, **tid**: three times daily

2.5.3 AUC and C_{max} goodness-of-fit plots

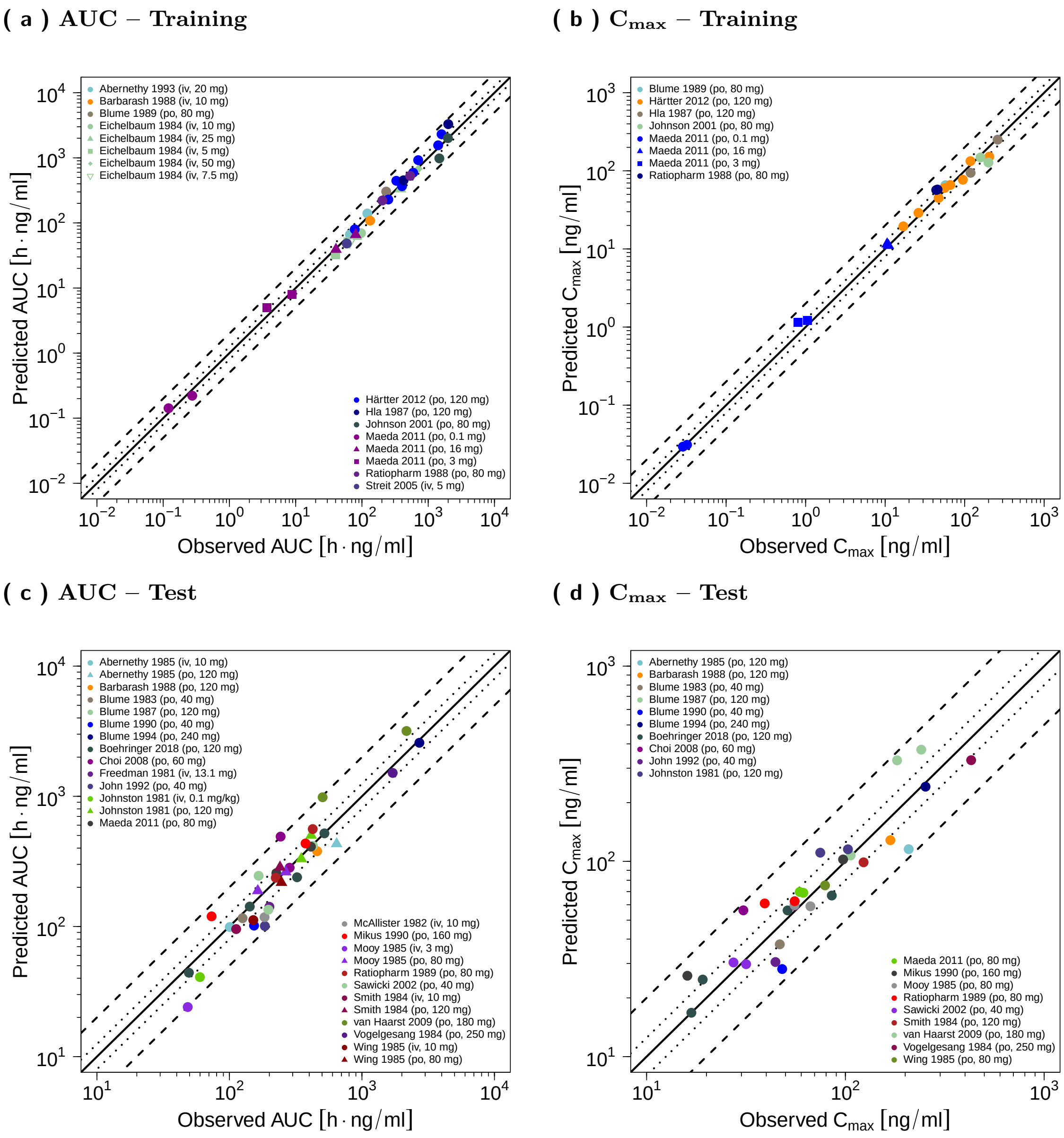

Figure S2.5.2: Predicted versus observed verapamil and norverapamil AUC and C_{max} values for the training and test datasets. Each symbol represents the AUC or C_{max} of a different study profile. The solid line (——) marks the line of identity. Dotted lines (·····) indicate 1.25-fold, dashed lines (- -) indicate 2-fold deviation. **AUC**: area under the plasma concentration–time curve from the time of administration to the last observed data point, **C_{max}**: maximum plasma concentration

2.5.4 Geometric mean fold error of predicted AUC and C_{max} values

Table S2.5.2: Predicted and observed AUC and C_{max} values of verapamil and norverapamil plasma concentrations

			AUC			C_{max}			
Route	Compound	Dose	Pred [h·ng/ml]	Obs [h·ng/ml]	Pred/Obs	Pred [ng/ml]	Obs [ng/ml]	Pred/Obs	Reference
**Intravenous**									
iv, 5 min	R-Verapamil	5.00 mg	64.75	84.53	0.77	-	-	-	Eichelbaum et al. 1984 [9]
iv, 5 min	R-Verapamil	25.00 mg	332.72	381.44	0.87	-	-	-	Eichelbaum et al. 1984 [9]
iv, 5 min	R-Verapamil	50.00 mg	678.00	712.72	0.95	-	-	-	Eichelbaum et al. 1984 [9]
iv, 5 min	S-Verapamil	5.00 mg	32.60	39.74	0.82	-	-	-	Eichelbaum et al. 1984 [9]
iv, 5 min	S-Verapamil	7.50 mg	52.16	65.35	0.80	-	-	-	Eichelbaum et al. 1984 [9]
iv, 5 min	S-Verapamil	10.00 mg	69.66	96.77	0.72	-	-	-	Eichelbaum et al. 1984 [9]
iv, 5 min	Verapamil	3.00 mg	24.06	48.44	0.50	-	-	-	Mooy et al. 1985 [10]
iv, 10 min	Verapamil	5.00 mg	48.31	58.98	0.82	-	-	-	Streit et al. 2005 [11]
iv, 5 min	Verapamil	0.10 mg/kg	40.85	59.86	0.68	-	-	-	Johnston et al. 1981 [12]
iv, 10 min	Verapamil	10.00 mg	99.11	100.61	0.99	-	-	-	Abernethy et al. 1985 [13]
iv, 10 min	Verapamil	10.00 mg	108.57	133.05	0.82	-	-	-	Barbarash et al. 1988 [14]
iv, 10 min	Verapamil	10.00 mg	112.01	151.40	0.74	-	-	-	Wing et al. 1985 [15]
iv, 5 min	Verapamil	10.00 mg	117.91	184.07	0.64	-	-	-	McAllister, Kirsten 1982 [16]
iv, 5 min	Verapamil	10.00 mg	95.52	112.39	0.85	-	-	-	Smith et al. 1984 [17]
iv, 13 min	Verapamil	13.10 mg	141.72	200.20	0.71	-	-	-	Freedman et al. 1981 [18]
iv, 30 min	R-Verapamil	20.00 mg	139.84	120.64	1.16	-	-	-	Abernethy et al. 1993 [19]
iv, 30 min	S-Verapamil	20.00 mg	66.30	65.68	1.01	-	-	-	Abernethy et al. 1993 [19]
**GMFE**					**1.27 (1.01–2.00)**<br>**17/17 with GMFE ≤ 2**				
**Oral**									
po, sol	R-Verapamil	250.00 mg	1512.87	1705.65	0.89	329.92	429.36	0.77	Vogelgesang et al. 1984 [20]
po, sol	Verapamil	0.10 mg	0.14	0.12	1.18	0.03	0.03	1.03	Maeda et al. 2011 [21]
po, sol	Norverapamil	0.10 mg	0.22	0.27	0.81	0.03	0.03	0.97	Maeda et al. 2011 [21]
po, tab	Verapamil	3.00 mg	4.99	3.68	1.36	1.15	0.80	1.44	Maeda et al. 2011 [21]
po, tab	Norverapamil	3.00 mg	8.00	8.76	0.91	1.21	1.05	1.15	Maeda et al. 2011 [21]
po, sol	Verapamil	16.00 mg	39.14	40.53	0.97	11.05	11.00	1.01	Maeda et al. 2011 [21]
po, sol	Norverapamil	16.00 mg	66.08	80.44	0.82	11.55	10.59	1.09	Maeda et al. 2011 [21]
po, drag	Verapamil	40.00 mg	115.79	125.57	0.92	37.61	46.77	0.80	Blume, Mutschler 1983 [22]
po, tab	Verapamil	40.00 mg	101.56	153.37	0.66	28.10	48.09	0.58	Blume, Mutschler 1990 [22]
po, tab	Verapamil	40.00 mg	100.81	185.01	0.55	30.52	44.50	0.69	John et al. 1992 [23]
po, tab	Verapamil	40.00 mg	134.66	196.88	0.68	29.76	31.67	0.94	Sawicki, Janicki 2002 [24]
po, tab	Norverapamil	40.00 mg	245.20	166.13	1.48	30.38	27.35	1.11	Sawicki, Janicki 2002 [24]
po, caps	Verapamil	60.00 mg	282.88	284.47	0.99	59.37	55.49	1.07	Choi et al. 2008 [25]
po, caps	Norverapamil	60.00 mg	490.42	243.34	2.02	56.16	30.67	1.83	Choi et al. 2008 [25]
po, -	Verapamil	80.00 mg	188.05	163.63	1.15	58.88	66.70	0.88	Mooy et al. 1985 [10]
po, -	Norverapamil	80.00 mg	262.33	266.23	0.99	59.64	55.50	1.08	Mooy et al. 1985 [10]
po, -	Verapamil	80.00 mg	217.71	246.81	0.88	75.37	78.98	0.95	Wing et al. 1985 [15]
po, sol	Verapamil	80.00 mg	257.10	226.06	1.14	69.76	58.93	1.18	Maeda et al. 2011 [21]

-: not given/not calculated, **bid**: twice daily, **caps**: capsule, **drag**: dragée, **fed**: administration with a meal, **GMFE**: geometric mean fold error, **IR**: Isoptin film-coated tablet, **iv**: intravenous **obs**: observed, **po**: oral, **pred**: predicted, **qd**: once daily, **SR**: Isoptin RR retard formulation, **sol**: solution, **tab**: tablet, **tid**: three times daily

Table S2.5.2: Predicted and observed AUC and C_{max} values of verapamil and norverapamil *(continued)*

			AUC			C_{max}			
Route	Compound	Dose	Pred [h·ng/ml]	Obs [h·ng/ml]	Pred/Obs	Pred [ng/ml]	Obs [ng/ml]	Pred/Obs	Reference
po, sol	Norverapamil	80.00 mg	410.32	411.85	1.00	69.08	61.46	1.12	Maeda et al. 2011 [21]
po, tab	Verapamil	80.00 mg	303.23	232.40	1.31	65.54	57.40	1.14	Blume, Mutschler 1989 [22]
po, tab	Verapamil	80.00 mg	221.30	203.46	1.09	56.90	43.95	1.30	Ratiopharm 1988 [26]
po, tab	Norverapamil	80.00 mg	529.62	535.03	0.99	57.67	45.57	1.27	Ratiopharm 1988 [26]
po, tab	Verapamil	80.00 mg	236.69	223.71	1.06	62.55	55.57	1.13	Ratiopharm 1989 [26]
po, tab	Norverapamil	80.00 mg	560.42	425.06	1.32	60.94	39.29	1.55	Ratiopharm 1989 [26]
po, caps	Verapamil	80.00 mg (tid)	980.97	1474.22	0.67	127.92	199.20	0.64	Johnson et al. 2001 [27]
po, caps	Norverapamil	80.00 mg (tid)	2006.67	1982.81	1.01	148.45	158.30	0.94	Johnson et al. 2001 [27]
po, -	Verapamil	120.00 mg	378.87	458.97	0.83	128.42	168.46	0.76	Barbarash et al. 1988 [14]
po, -	Verapamil	120.00 mg	285.28	240.70	1.19	98.94	123.48	0.80	Smith et al. 1984 [17]
po, IR	R-Verapamil	120.00 mg	238.80	323.86	0.74	66.86	85.44	0.78	Boehringer 2018 [28]
po, IR	R-Norverapamil	120.00 mg	519.41	521.00	1.00	56.10	51.20	1.10	Boehringer 2018 [28]
po, IR	S-Verapamil	120.00 mg	44.16	49.43	0.89	16.78	16.79	1.00	Boehringer 2018 [28]
po, IR	S-Norverapamil	120.00 mg	142.25	142.40	1.00	24.82	19.15	1.30	Boehringer 2018 [28]
po, IR	R-Verapamil	120.00 mg	366.72	402.22	0.91	76.97	95.09	0.81	Härtter et al. 2012 [29]
po, IR	R-Norverapamil	120.00 mg	591.31	592.63	1.00	66.04	66.00	1.00	Härtter et al. 2012 [29]
po, IR	S-Verapamil	120.00 mg	79.72	77.90	1.02	19.49	16.95	1.15	Härtter et al. 2012 [29]
po, IR	S-Norverapamil	120.00 mg	228.91	250.05	0.92	29.01	26.28	1.10	Härtter et al. 2012 [29]
po, tab	Verapamil	120.00 mg	431.51	643.82	0.67	115.48	208.24	0.56	Abernethy et al. 1985 [13]
po, tab	Verapamil	120.00 mg	416.36	427.11	0.98	107.27	106.33	1.01	Blume, Mutschler 1987 [22]
po, tab	Verapamil	120.00 mg	452.31	424.51	1.07	94.45	118.87	0.80	Hla et al. 1987 [30]
po, tab	Verapamil	120.00 mg	331.26	348.40	0.95	115.36	103.00	1.12	Johnston et al. 1981 [12]
po, tab	Norverapamil	120.00 mg	502.03	413.68	1.21	110.84	74.76	1.48	Johnston et al. 1981 [12]
po, IR	R-Verapamil	120.00 mg (bid)	1558.54	1414.09	1.10	152.25	204.36	0.75	Härtter et al. 2012 [29]
po, IR	R-Norverapamil	120.00 mg (bid)	2307.58	1591.51	1.45	133.25	118.44	1.13	Härtter et al. 2012 [29]
po, IR	S-Verapamil	120.00 mg (bid)	443.96	330.07	1.35	45.00	47.09	0.96	Härtter et al. 2012 [29]
po, IR	S-Norverapamil	120.00 mg (bid)	922.57	705.73	1.31	60.47	56.07	1.08	Härtter et al. 2012 [29]
po, tab	Verapamil	120.00 mg (bid)	3294.92	2007.74	1.64	251.11	260.40	0.96	Hla et al. 1987 [30]
po, sol	R-Verapamil	160.00 mg	434.61	375.28	1.16	102.33	97.38	1.05	Mikus et al. 1990 [31]
po, sol	S-Verapamil	160.00 mg	119.67	73.32	1.63	25.96	16.04	1.62	Mikus et al. 1990 [31]
po, -, fed	Verapamil	180.00 mg (bid)	3176.72	2168.85	1.47	373.47	240.95	1.55	van Haarst et al. 2009 [32]
po, -, fed	Norverapamil	180.00 mg (bid)	983.17	504.90	1.95	329.35	182.00	1.81	van Haarst et al. 2009 [32]
po, SR	Verapamil	240.00 mg (qd)	2578.10	2713.10	0.95	241.35	253.08	0.95	Blume, Mutschler 1994 [22]
**GMFE**					**1.22 (1.00–2.02)**			**1.22 (1.00–1.83)**	
					**50/51 with GMFE ≤ 2**			**51/51 with GMFE ≤ 2**	
**Overall GMFE**					**1.24 (1.00–2.02)**			**1.22 (1.00–1.83)**	
					**67/68 with GMFE ≤ 2**			**51/51 with GMFE ≤ 2**	

-: not given/not calculated, **bid**: twice daily, **caps**: capsule, **drag**: dragée, **fed**: administration with a meal, **GMFE**: geometric mean fold error, **IR**: Isoptin film-coated tablet, **iv**: intravenous **obs**: observed, **po**: oral, **pred**: predicted, **qd**: once daily, **SR**: Isoptin RR retard formulation, **sol**: solution, **tab**: tablet, **tid**: three times daily

2.5.5 Sensitivity analysis

Sensitivity of the verapamil model to single parameters (local sensitivity analysis) was calculated as the relative change of the predicted total verapamil $AUC_{0\text{-}24}$ (Figure S2.5.3) and of the predicted total norverapamil $AUC_{0\text{-}24}$ (Figure S2.5.4) following a single dose of 120 mg racemic verapamil administered as immediate release formulation. Sensitivity analysis was carried out using a relative parameter perturbation of 1000 % (variation range 10.0, maximum number of 9 steps). Parameters were included into the analysis if they were optimized (CYP3A4 and Pgp k_{cat} values, lipophilicity, intestinal permeability, cellular permeability, Pgp non-competitive inhibition K_i), if they are associated with optimized parameters (CYP3A4 and Pgp K_m values, CYP3A4 MBI K_I values, CYP3A4 MBI k_{inact} values) or if they might have a strong impact due to calculation methods used in the model (solubility, fraction unbound in plasma, GFR fraction).

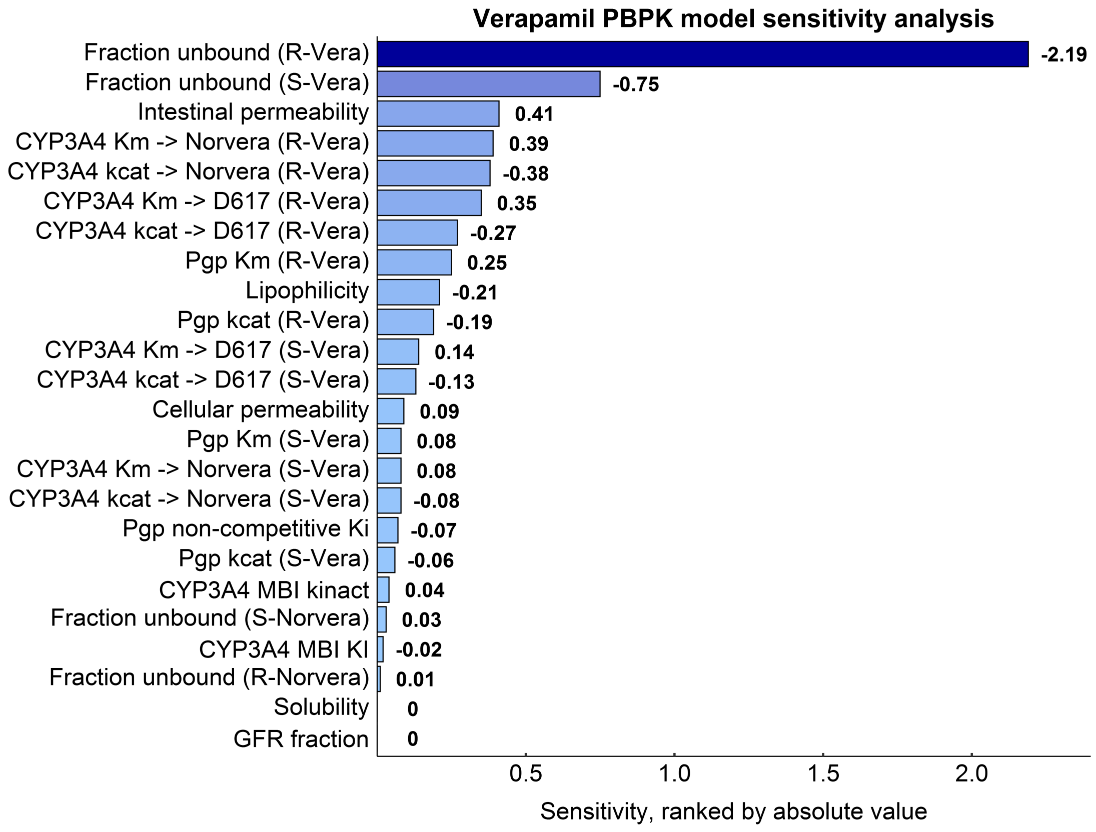

Figure S2.5.3: Verapamil PBPK model sensitivity analysis. Sensitivity of the model to single parameters, calculated as change of the simulated total verapamil $AUC_{0\text{-}24}$ following a single dose of 120 mg racemic verapamil as immediate release formulation. To simplify the plot, parameters that are modeled with the same parameter value for all 4 compounds (namely lipophilicity, intestinal permeability, cellular permeability, GFR fraction, solubility and Pgp K_i) are plotted only once, showing the sensitivity to the most impacting of the 4 compounds. CYP3A4 MBI K_I and CYP3A4 MBI k_{inact} sensitivity values are also reduced to the value of the one compound that the model is most sensitive to. **D617**: verapamil metabolite, **GFR**: glomerular filtration rate, **kcat**: catalytic rate constant (turnover number), **KI**: concentration for half-maximal inactivation, **Ki**: concentration for half-maximal inhibition, **kinact**: maximum inactivation rate, **Km**: Michaelis-Menten constant, **MBI**: mechanism-based inactivation, **Norvera**: norverapamil, **Vera**: verapamil

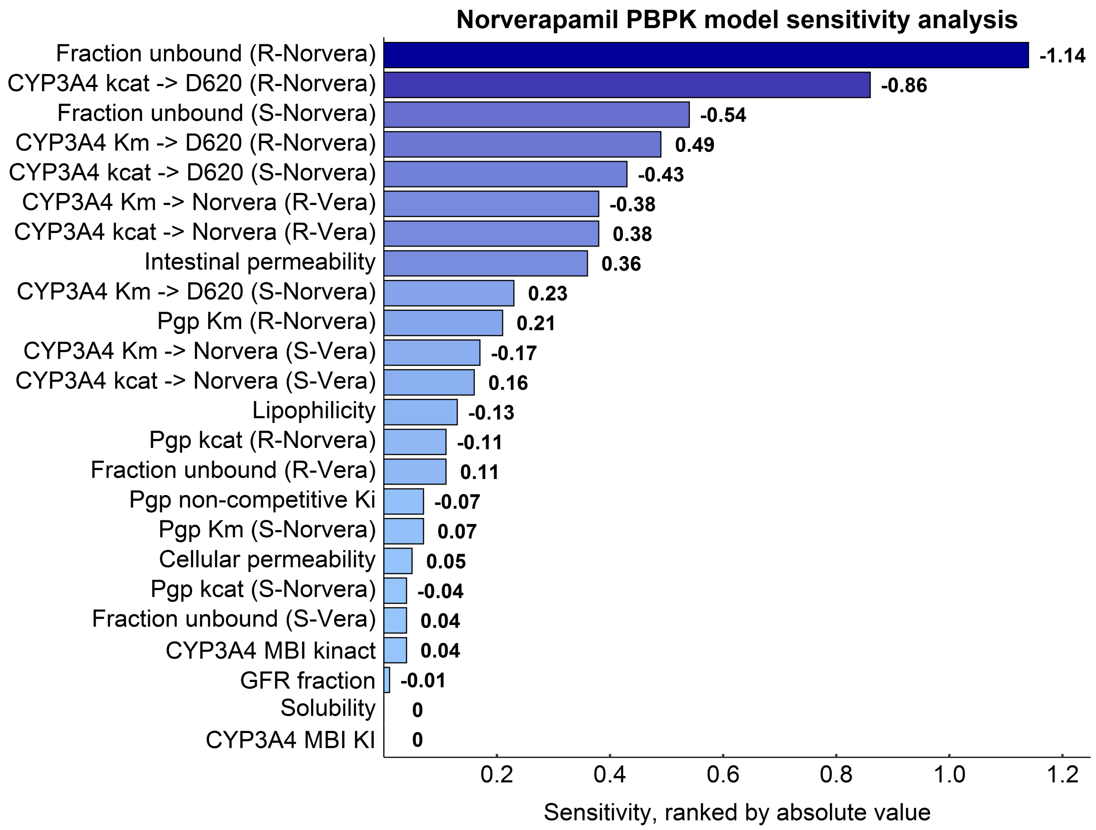

Figure S2.5.4: Norverapamil PBPK model sensitivity analysis. Sensitivity of the model to single parameters, calculated as change of the simulated total norverapamil $AUC_{0\text{-}24}$ following a single dose of 120 mg racemic verapamil as immediate release formulation. To simplify the plot, parameters that are modeled with the same parameter value for all 4 compounds (namely lipophilicity, intestinal permeability, cellular permeability, GFR fraction, solubility and Pgp K_i) are plotted only once, showing the sensitivity to the most impacting of the 4 compounds. CYP3A4 MBI K_I and CYP3A4 MBI k_{inact} sensitivity values are also reduced to the value of the one compound that the model is most sensitive to. **D620**: norverapamil metabolite, **GFR**: glomerular filtration rate, **kcat**: catalytic rate constant (turnover number), **KI**: concentration for half-maximal inactivation, **Ki**: concentration for half-maximal inhibition, **kinact**: maximum inactivation rate, **Km**: Michaelis-Menten constant, **MBI**: mechanism-based inactivation, **Norvera**: norverapamil, **Vera**: verapamil

3 Verapamil-midazolam drug-drug interaction (DDI)

3.1 DDI modeling

The verapamil-midazolam DDI was predicted using a previously established whole-body PBPK model of midazolam [46]. The drug-dependent parameters of this model are reproduced in Table S3.2.1.

The verapamil-midazolam interaction was modeled as mechanism-based inactivation of CYP3A4 midazolam metabolism using the intrinsic mechanism-based auto-inactivation processes that are part of the verapamil model to describe the auto-inactivation of CYP3A4 by R-verapamil, S-verapamil, R-norverapamil and S-norverapamil. K_I (corrected for binding in the microsomal assay) and k_{inact} values for these inactivation processes were obtained from in vitro literature [38] and are listed in the verapamil and norverapamil drug-dependent parameter tables (Tables S2.3.1 and S2.3.2).

Details on the predicted clinical DDI studies are given in Table S3.3.1. Model predictions of midazolam plasma concentration-time profiles before and during verapamil co-administration, compared to observed data, are shown in Figures S3.4.1 and S3.4.2. The correlation of predicted to observed DDI AUC ratios (AUC of the victim drug during perpetrator treatment/AUC of the victim drug alone) and DDI C_{max} ratios (C_{max} of the victim drug during perpetrator treatment/C_{max} of the victim drug alone) is shown in Figure S3.5.1. Table S3.5.1 lists the corresponding predicted and observed DDI AUC ratios, DDI C_{max} ratios, as well as GMFE values.

3.2 Midazolam drug-dependent parameters

The drug-dependent parameters of the midazolam model are summarized in Table S3.2.1 below. The associated system-dependent parameters are listed in Table S7.0.1.

Table S3.2.1: Drug-dependent parameters of the midazolam PBPK model (adopted from [46])

Parameter	Value	Unit	Source	Literature	Reference	Description
MW	325.77	g/mol	Literature	325.77	[33]	Molecular weight
pKa (base)	6.15	-	Literature	6.15	[47]	Acid dissociation constant
Solubility (pH 6.5)	0.049	g/l	Literature	0.049	[48]	Solubility
logP	3.13	-	Optimized	2.9, 3.9	[49, 50]	Lipophilicity
fu	1.6	%	Literature	1.6, 2.4	[49, 51]	Fraction unbound
CYP3A4 K_m	2.73	µmol/l	Literature	2.73	[52]	CYP3A4 Michaelis-Menten constant
CYP3A4 k_{cat}	13.0	1/min	Optimized	-	-	CYP3A4 catalytic rate constant
GFR fraction	1.00	-	Assumed	-	-	Fraction of filtered drug in the urine
EHC continuous fraction	1.00	-	Assumed	-	-	Fraction of bile continually released
Partition coefficients	Diverse	-	Calculated	R&R	[41, 42]	Cell to plasma partition coefficients
Cellular permeability	6.98E-02	cm/min	Calculated	PK-Sim	[2]	Permeability into the cellular space
Intestinal permeability	2.00E-05	cm/min	Optimized	1.88E-04	Calculated	Transcellular intestinal permeability
Tablet Weibull time	26.96	min	Optimized	-	-	Dissolution time (50% dissolved)
Tablet Weibull shape	0.70	-	Optimized	-	-	Dissolution profile shape

EHC: enterohepatic circulation, **GFR**: glomerular filtration rate, **PK-Sim**: PK-Sim standard calculation method, **R&R**: Rodgers and Rowland calculation method

3.3 Verapamil-midazolam clinical DDI studies

The clinical studies used to evaluate the verapamil-midazolam DDI model performance are summarized in Table S3.3.1.

Table S3.3.1: Verapamil-midazolam DDI study table

**Perpetrator** Verapamil	**Victim** Midazolam	**Dose gap [h]**	**n**	**Men** [%]	**Age** [years]	**Weight** [kg]	**Height** [cm]	**BMI** [kg/m^2]	**Dataset**	**Reference**
240 mg, po, SR, qd	0.05 mg/kg, iv, 30 min	0	8 [a]	-	-	-	-	-	test	Wang et al. 2005 [53]
240 mg, po, SR, qd	4 mg, po, sol	0	8 [a]	-	-	-	-	-	test	Wang et al. 2005 [53]
80 mg, po, tab, tid	15 mg, po, tab	1	9	0	(19-28)	(55-80)	-	-	test	Backman et al. 1994 [54]

[a] CYP3A5*3/*3 genotype i.e. CYP3A5 non-expressors, **-**: not given, **BMI**: body mass index, **iv**: intravenous, **n**: number of individuals studied, **po**: oral, **qd**: once daily, **SR**: sustained release, **sol**: solution, **tab**: tablet, **test**: test dataset (model evaluation), **tid**: three times daily

3.4 Profiles

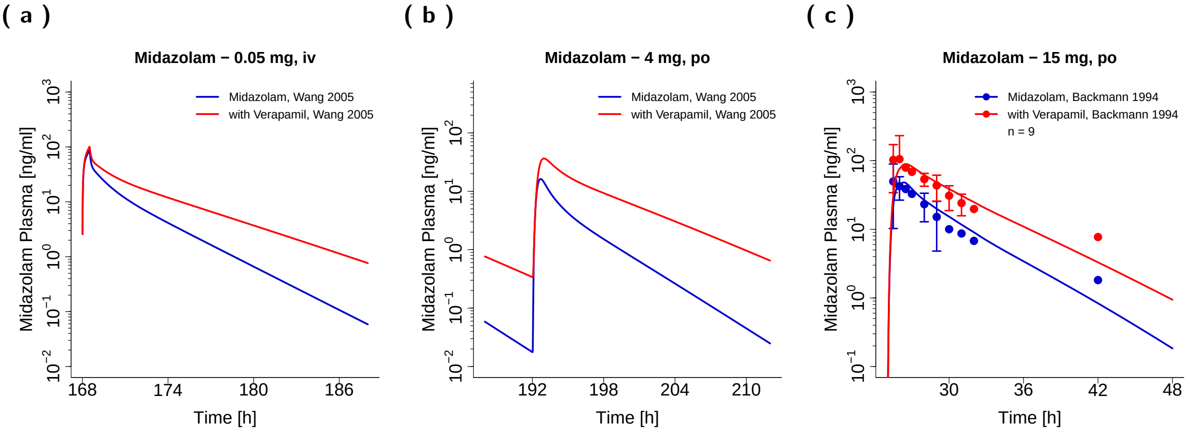

Figure S3.4.1: Midazolam plasma concentration-time profiles (semilogarithmic) before and during verapamil cotreatment. Observed data are shown as dots, if available ± standard deviation (SD). Simulations are shown as lines. The study by Wang et al. [53] only reported DDI AUC ratios without the associated plasma concentration-time profiles.

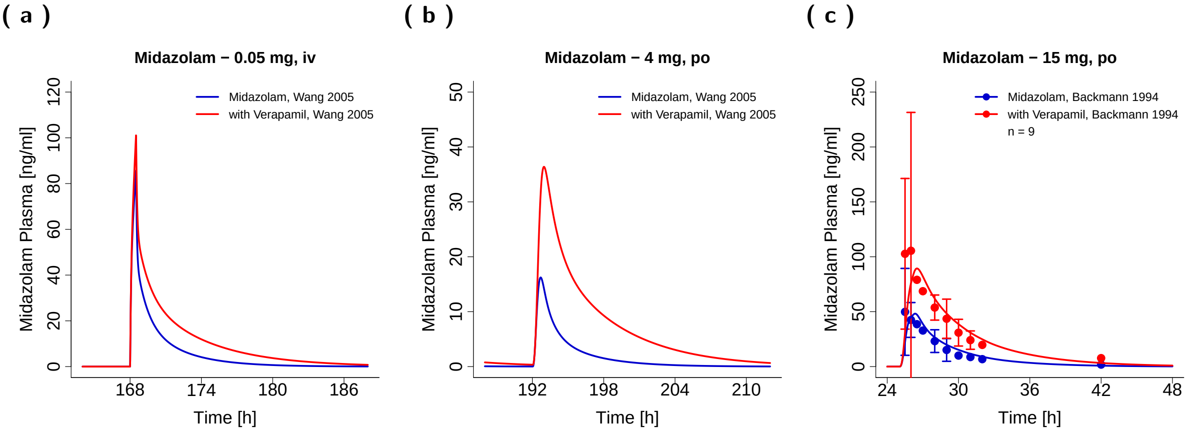

Figure S3.4.2: Midazolam plasma concentration-time profiles (linear) before and during verapamil cotreatment. Observed data are shown as dots, if available ± standard deviation (SD). Simulations are shown as lines. The study by Wang et al. [53] only reported DDI AUC ratios without the associated plasma concentration-time profiles.

3.5 Model evaluation

3.5.1 DDI AUC and C_{max} ratio goodness-of-fit plots

(a) DDI AUC ratios

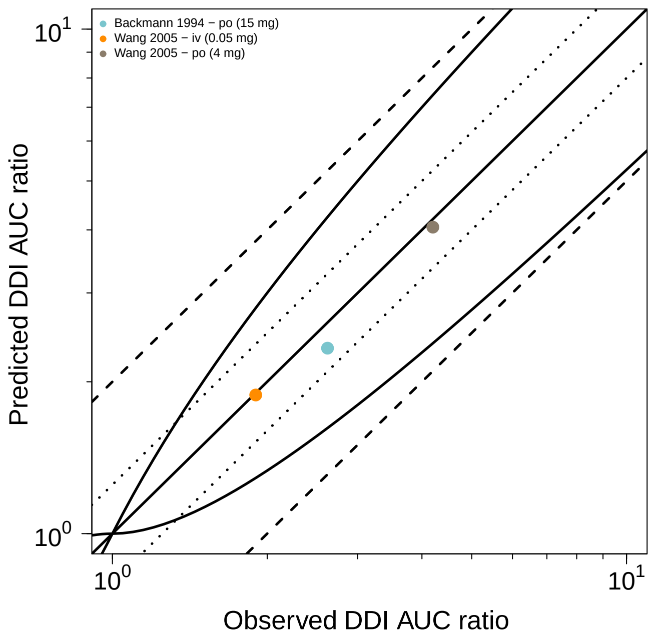

(b) DDI C_{max} ratios

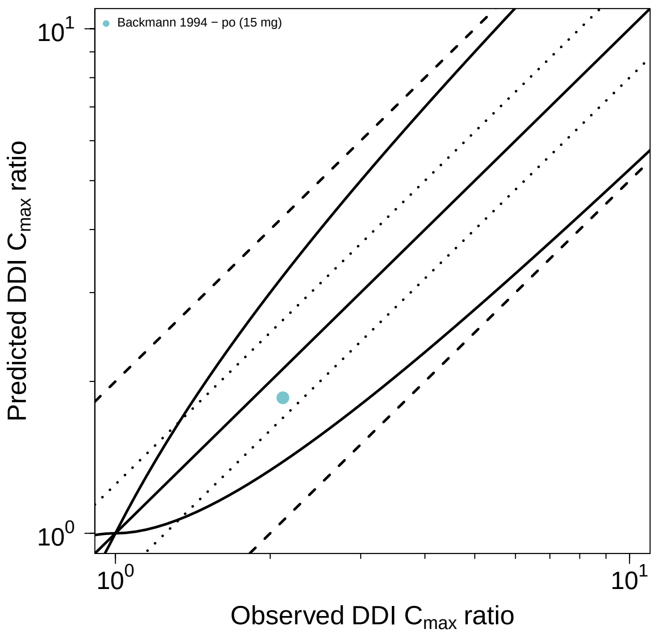

Figure S3.5.1: Predicted versus observed verapamil-midazolam DDI AUC ratios and DDI C_{max} ratios. Each symbol represents the DDI AUC or C_{max} ratio of a different study profile. The straight solid line (—) marks the line of identity. The dotted lines (⋯⋯) indicate 1.25-fold, the dashed lines (- -) indicate 2-fold deviation. The curved lines show the prediction success limits suggested by Guest et al. [55]. **AUC**: area under the plasma concentration–time curve from the time of administration to the last observed data point, **C_{max}**: maximum plasma concentration

3.5.2 Geometric mean fold error of predicted DDI AUC and C_{max} ratios

Table S3.5.1: Predicted and observed verapamil-midazolam DDI AUC ratios and DDI C_{max} ratios

**Perpetrator**	**Victim**			**DDI AUC ratio**			**DDI C_{max} ratio**			
Verapamil	Midazolam	Dose gap [h]	n	Pred	Obs	Pred/Obs	Pred	Obs	Pred/Obs	Reference
240 mg, po, SR, qd	0.05 mg/kg, iv, 30 min	0	8 [a]	1.88	1.90 [b]	0.99	-	-	-	Wang et al. 2005 [53]
240 mg, po, SR, qd	4 mg, po, sol	0	8 [a]	4.05	4.20 [b]	0.96	-	-	-	Wang et al. 2005 [53]
80 mg, po, tab, tid	15 mg, po, tab	1	9	2.33	2.62	0.89	1.86	2.12	0.88	Backman et al. 1994 [54]
**Overall GMFE**						**1.06 (1.01–1.12)**			**1.14**	
						**3/3 with GMFE ≤ 2**			**1/1 with GMFE ≤ 2**	

[a] CYP3A5*3/*3 genotype i.e. CYP3A5 non-expressors, [b] as stated in the reference, **-**: not given, **GMFE**: geometric mean fold error, **iv**: intravenous, **n**: number of individuals studied, **obs**: observed, **po**: oral, **pred**: predicted, **qd**: once daily, **SR**: sustained release, **sol**: solution, **tab**: tablet, **tid**: three times daily

4 Verapamil-digoxin drug-drug interaction (DDI)

4.1 DDI modeling

The verapamil-digoxin DDI was modeled using a previously established whole-body PBPK model of digoxin [46]. The drug-dependent parameters of this model are reproduced in Table S4.2.1.

The verapamil-digoxin interaction was modeled as non-competitive inhibition of Pgp digoxin transport by R-verapamil, S-verapamil, R-norverapamil and S-norverapamil. Non-stereospecific, equipotent inhibition by all 4 compounds was assumed, as described in the literature [56, 57]. The K_i = 0.038 µmol/l (see Tables S2.3.1 and S2.3.2) to model the Pgp inhibition was optimized using one of the 10 clinical verapamil-digoxin DDI studies [58] and was then applied to predict the remaining 9 studies.

Details on the modeled clinical DDI studies are given in Table S4.3.1. Model predictions of digoxin plasma concentration-time profiles before and during verapamil co-administration, compared to observed data, are shown in Figures S4.4.1 and S4.4.2. The correlation of predicted to observed DDI AUC ratios, DDI C_{max} ratios and DDI C_{trough} ratios is shown in Figure S4.5.1. Table S4.5.1 lists the corresponding predicted and observed DDI AUC ratios, DDI C_{max} ratios, DDI C_{trough} ratios, as well as GMFE values.

4.2 Digoxin drug-dependent parameters

The drug-dependent parameters of the digoxin model are summarized in Table S4.2.1 below. The associated system-dependent parameters are listed in Table S7.0.1.

Table S4.2.1: Drug-dependent parameters of the digoxin PBPK model (adopted from [46])

Parameter	Value	Unit	Source	Literature	Reference	Description
MW	780.93	g/mol	Literature	780.93	[33]	Molecular weight
pKa	-	-	Literature	-	[59]	Acid dissociation constant
Solubility (water)	0.0648	g/l	Literature	0.0648	[60]	Solubility
logP	1.40	-	Optimized	1.22 - 1.67	[61–63]	Lipophilicity
fu	71.0	%	Literature	71.0	[64]	Fraction unbound
ATP1A2 K_D	25.6	nmol/l	Literature	25.6	[65]	ATP1A2 dissociation constant
ATP1A2 k_{off}	9.89E-04	1/min	Optimized	-	-	ATP1A2 dissociation rate constant
Pgp K_m	177.0	µmol/l	Literature	177.0	[66]	Pgp Michaelis-Menten constant
Pgp k_{cat}	71.2	1/min	Optimized	-	-	Pgp transport rate constant
CL_{hep}	0.038	1/min	Optimized	-	-	Specific hepatic plasma clearance
GFR fraction	1.00	-	Assumed	-	-	Fraction of filtered drug in the urine
EHC continuous fraction	1.00	-	Assumed	-	-	Fraction of bile continually released
Partition coefficients	Diverse	-	Calculated	R&R	[41, 42]	Cell to plasma partition coefficients
Cellular permeability	1.01E-04	cm/min	Optimized	PK-Sim	[2]	Permeability into the cellular space
Intestinal permeability	2.76E-06	cm/min	Optimized	3.86E-08	Calculated	Transcellular intestinal permeability

ATP1A2: ATPase Na^+/K^+ transporting subunit alpha 2, **CL**: clearance, **EHC**: enterohepatic circulation, **GFR**: glomerular filtration rate, **Pgp**: P-glycoprotein, **PK-Sim**: PK-Sim standard calculation method, **R&R**: Rodgers and Rowland calculation method

4.3 Verapamil-digoxin clinical DDI studies

The clinical studies used to evaluate the verapamil-digoxin DDI model performance are summarized in Table S4.3.1.

Table S4.3.1: Verapamil-digoxin DDI study table

**Perpetrator** Verapamil	**Victim** Digoxin	**Dose gap [h]**	**n**	**Men** [%]	**Age [years]**	**Weight [kg]**	**Height [cm]**	**BMI [kg/m^2]**	**Dataset**	**Reference**
80 mg, po, - , tid	1.0 mg, iv, 15 min	0	12	100	30 (18-38)	75 (61-93)	-	-	test	Johnson et al. 1987 [67]
120 mg, po, - , tid	1.0 mg, iv, bolus	0	1	100	(21-32)	-	-	-	test	Pedersen et al. 1983 [68]
80 mg, po, - , tid	0.0625 mg, po, - , bid	0	7	86	(26-53)	-	-	-	training	Pedersen et al. 1982 [58]
80 mg, po, - , tid	0.125 mg, po, - , tid	0	12	100	(20-33)	(56-105)	-	-	test	Belz et al. 1983 [69]
80 mg, po, - , tid	0.125 mg, po, tab, tid	0	9	-	(26-45)	(51-71)	(164-176)	-	test	Doering 1983 [70]
120 mg, po, - , tid	0.125 mg, po, - , tid	0	12	100	(20-33)	(56-105)	-	-	test	Belz et al. 1983 [69]
120 mg, po, tab	0.25 mg, po, tab [a]	1	12	100	39 ± 12 (25-53)	86 ± 10 (72-104)	181 ± 6 (170-191)	26 ± 2 (22-29)	test	Boehringer 2018 [28]
80 mg, po, - , tid	0.25 mg, po, tab, qd	6	7 [b]	41	61 ± 10 (30-78)	-	-	-	test	Klein et al. 1982 [71]
80 mg, po, - , tid	0.25 mg, po, tab, bid	0.5	10	100	30 (23-40)	78 (62-94)	-	-	test	Rodin et al. 1988 [72]
80 mg, po, tab, qid	0.25-1.0 mg, po, tab, qd	21	10 [c]	70	61 ± 5 (52-69)	81 ± 21 (60-120)	-	-	test	Schwartz et al. 1982 [73]

[a] single dose of a 0.25 mg digoxin, 1.0 mg furosemide, 10 mg metformin and 10 mg rosuvastatin transporter probe drug cocktail, [b] chronic atrial fibrillation patients with different co-medications, [c] chronic atrial fibrillation patients with different comorbidities and co-medications, **-**: not given, **bid**: twice daily, **BMI**: body mass index, **iv**: intravenous, **n**: number of individuals studied, **po**: oral, **qd**: once daily, **qid**: four times daily, **tab**: tablet, **test**: test dataset (model evaluation), **tid**: three times daily, **training**: training dataset (model development and parameter optimization)

4.4 Profiles

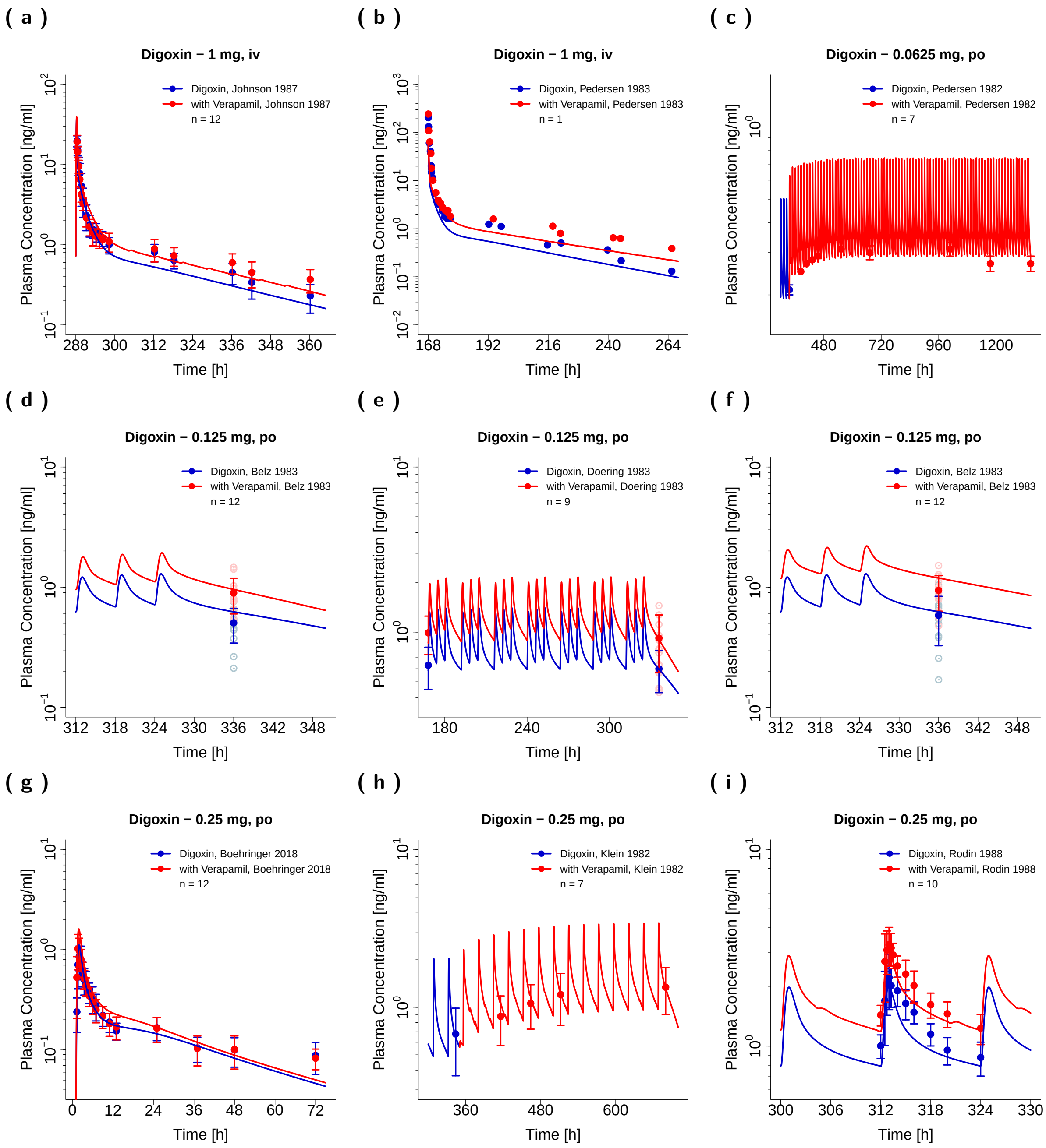

Figure S4.4.1: Digoxin plasma concentration-time profiles (semilogarithmic) before and during verapamil cotreatment. Observed data are shown as dots, if available ± standard deviation (SD); additional individual observed data are shown as light colored circles. Simulations are shown as lines.

(j)

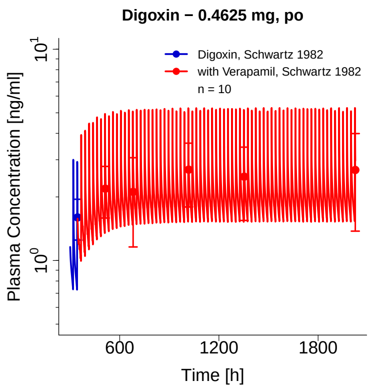

Figure S4.4.1: Digoxin plasma concentration-time profiles (semilogarithmic) before and during verapamil cotreatment. Observed data are shown as dots, if available ± standard deviation (SD); additional individual observed data are shown as light colored circles. Simulations are shown as lines. (continued)

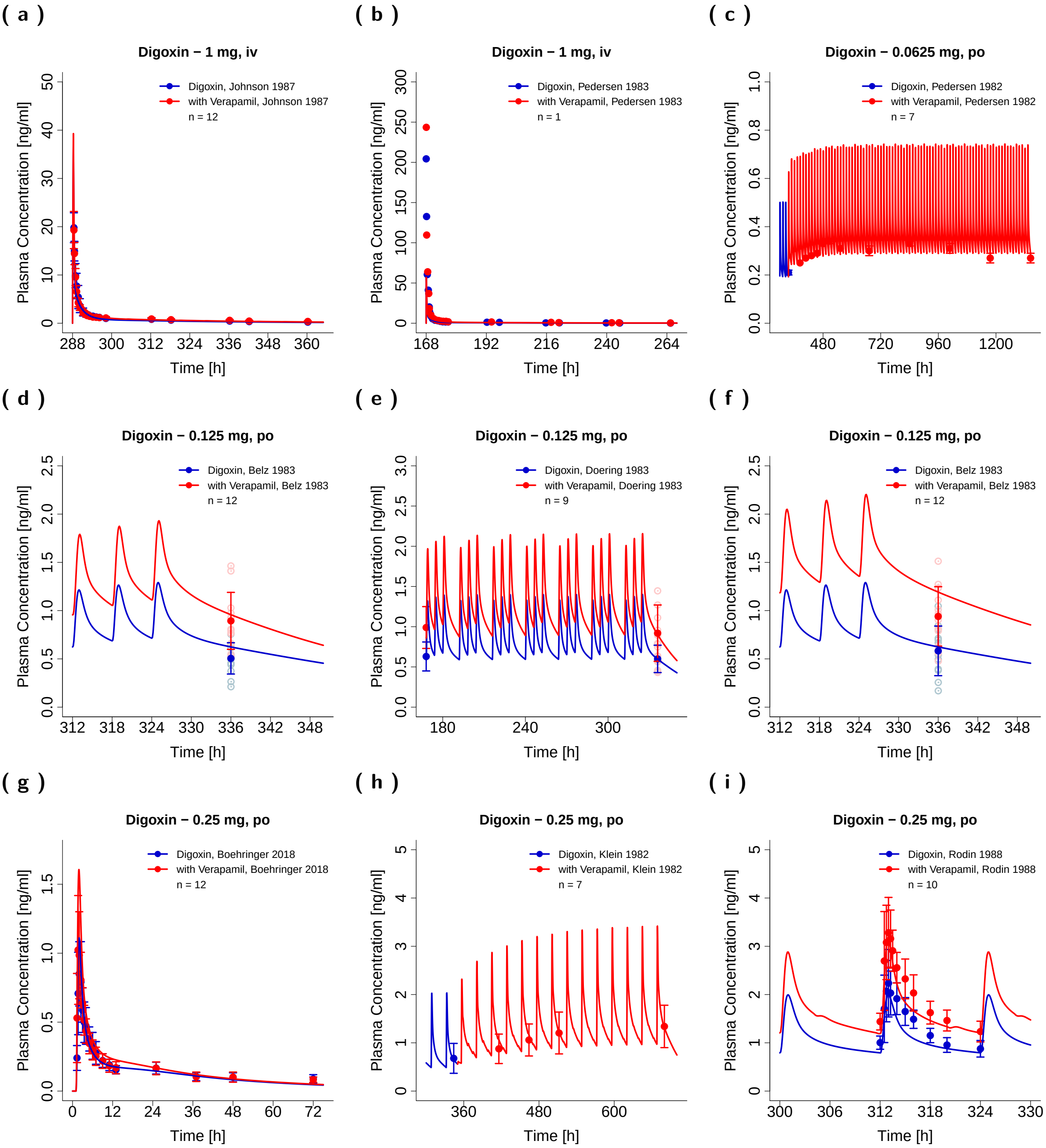

Figure S4.4.2: Digoxin plasma concentration-time profiles (linear) before and during verapamil cotreatment. Observed data are shown as dots, if available ± standard deviation (SD); additional individual observed data are shown as light colored circles. Simulations are shown as lines.

(j)

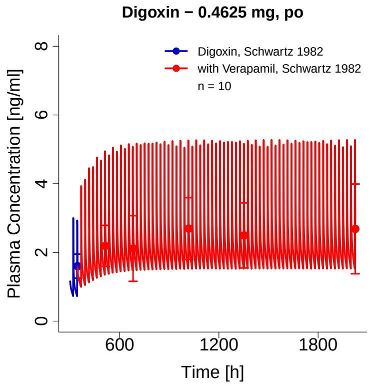

Figure S4.4.2: Digoxin plasma concentration-time profiles (linear) before and during verapamil cotreatment. Observed data are shown as dots, if available ± standard deviation (SD); additional individual observed data are shown as light colored circles. Simulations are shown as lines. (continued)

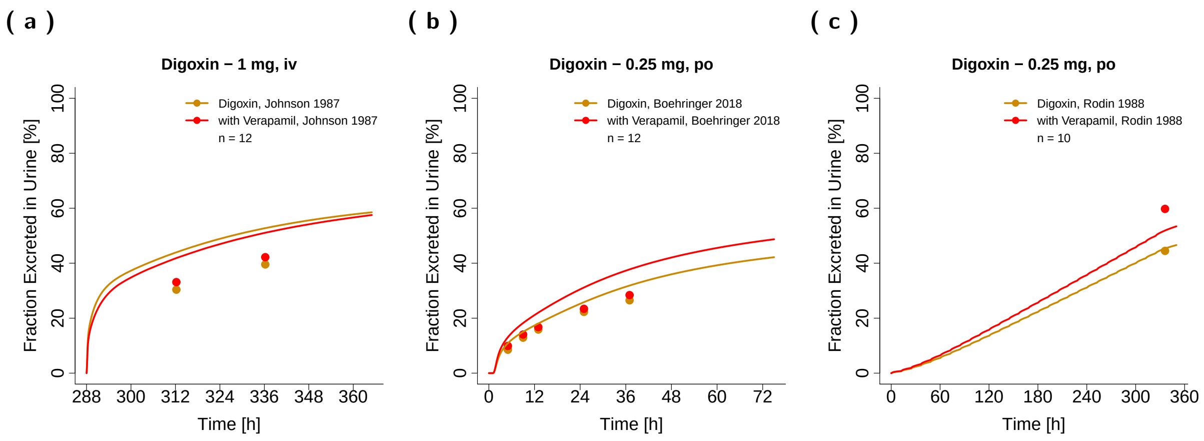

Figure S4.4.3: Digoxin fraction excreted in urine when administered alone and during verapamil cotreatment. Observed data are shown as dots. Simulations are shown as lines.

4.5 Model evaluation

4.5.1 DDI AUC, C_{max} and C_{trough} ratio goodness-of-fit plots

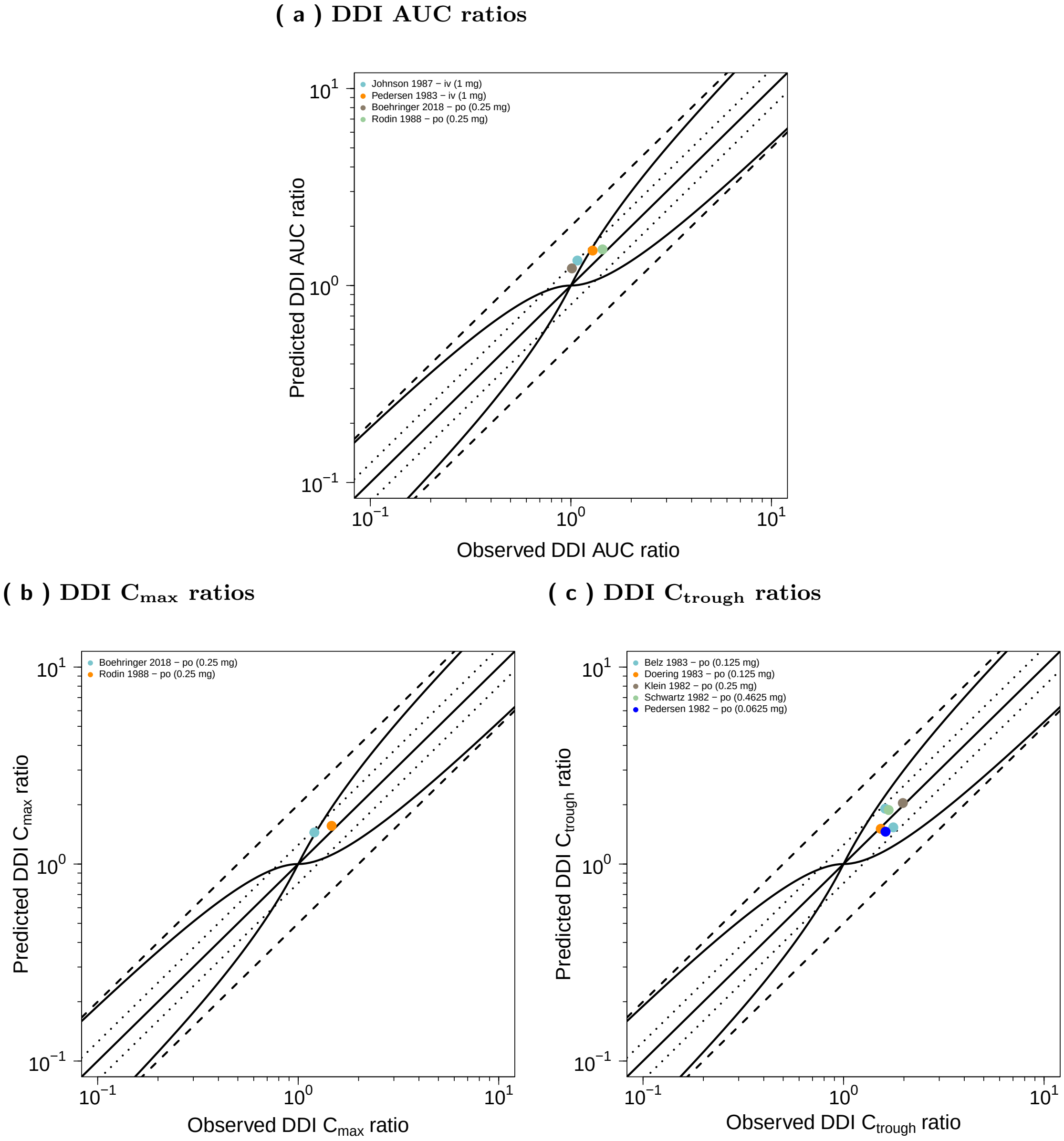

Figure S4.5.1: Predicted versus observed verapamil-digoxin DDI AUC ratios, DDI C_{max} ratios and DDI C_{trough} ratios. Each symbol represents the DDI AUC, C_{max} or C_{trough} ratio of a different study profile. The straight solid line (——) marks the line of identity. The dotted lines (·····) indicate 1.25-fold, the dashed lines (- -) indicate 2-fold deviation. The curved lines show the prediction success limits suggested by Guest et al. [55]. **AUC**: area under the plasma concentration–time curve from the time of administration to the last observed data point, $\mathbf{C_{max}}$: maximum plasma concentration, $\mathbf{C_{trough}}$: lowest plasma concentration before administration of the next dose

4.5.2 Geometric mean fold error of predicted DDI AUC, C_{max} and C_{trough} ratios

Table S4.5.1: Predicted and observed verapamil-digoxin DDI AUC ratios, DDI C_{max} ratios and DDI C_{trough} ratios

**Perpetrator**	**Victim**			**DDI AUC ratio**			**DDI C_{max} ratio**			**DDI C_{trough} ratio**			
Verapamil	Digoxin	Dose gap [h]	n	Pred	Obs	Pred/Obs	Pred	Obs	Pred/Obs	Pred	Obs	Pred/Obs	Reference
80 mg, po, - , tid	1.0 mg, iv, 15 min	0	12	1.34	1.08	1.24	-	-	-	-	-	-	Johnson et al. 1987 [67]
120 mg, po, - , tid	1.0 mg, iv, bolus	0	1	1.51	1.28	1.17	-	-	-	-	-	-	Pedersen et al. 1983 [68]
80 mg, po, - , tid	0.0625 mg, po, - , bid	0	7	-	-	-	-	-	-	1.46	1.62	0.90	Pedersen et al. 1982 [58]
80 mg, po, - , tid	0.125 mg, po, - , tid	0	12	-	-	-	-	-	-	1.54	1.77	0.87	Belz et al. 1983 [69]
80 mg, po, - , tid	0.125 mg, po, tab, tid	0	9	-	-	-	-	-	-	1.51	1.53	0.98	Doering 1983 [70]
120 mg, po, - , tid	0.125 mg, po, - , tid	0	12	-	-	-	-	-	-	1.91	1.61	1.18	Belz et al. 1983 [69]
120 mg, po, tab	0.25 mg, po, tab [a]	1	12	1.22	1.01	1.21	1.45	1.20	1.20	-	-	-	Boehringer 2018 [28]
80 mg, po, - , tid	0.25 mg, po, tab, qd	6	7 [b]	-	-	-	-	-	-	2.04	1.98	1.03	Klein et al. 1982 [71]
80 mg, po, - , tid	0.25 mg, po, tab, bid	0.5	10	1.53	1.44	1.06	1.56	1.47	1.06	-	-	-	Rodin et al. 1988 [72]
80 mg, po, tab, qid	0.25-1.0 mg, po, tab, qd	21	10 [c]	-	-	-	-	-	-	1.88	1.68	1.12	Schwartz et al. 1982 [73]
**Overall GMFE**						**1.17 (1.06–1.24)**			**1.13 (1.06–1.20)**			**1.10 (1.02–1.18)**	
						**4/4 with GMFE ≤ 2**			**2/2 with GMFE ≤ 2**			**6/6 with GMFE ≤ 2**	

[a] single dose of a 0.25 mg digoxin, 1.0 mg furosemide, 10 mg metformin and 10 mg rosuvastatin transporter probe drug cocktail, [b] chronic atrial fibrillation patients with different co-medications, [c] chronic atrial fibrillation patients with different comorbidities and co-medications, **-**: not given, **bid**: twice daily, **GMFE**: geometric mean fold error, **iv**: intravenous, **n**: number of individuals studied, **obs**: observed, **po**: oral, **pred**: predicted, **qd**: once daily, **qid**: four times daily, **tab**: tablet, **tid**: three times daily

5 Rifampicin-verapamil drug-drug interaction (DDI)

5.1 DDI modeling

The rifampicin-verapamil DDI was predicted using a previously established whole-body PBPK model of rifampicin [46]. The drug-dependent parameters of this model are reproduced in Table S5.2.1.

The rifampicin interaction was modeled as induction of CYP3A4 verapamil metabolism and Pgp verapamil transport with simultaneous competitive inhibition of CYP3A4 and Pgp by rifampicin. The parameters to model these interactions were obtained from literature (see Table S5.2.1) and have been qualified previously in several different DDI predictions [46, 74].

Details on the predicted clinical DDI studies are given in Table S5.3.1. Model predictions of verapamil plasma concentration-time profiles before and during rifampicin co-administration, compared to observed data, are shown in Figures S5.4.1 and S5.4.2. The correlation of predicted to observed DDI AUC ratios and DDI C_{max} ratios is shown in Figure S5.5.1. Table S5.5.1 lists the corresponding predicted and observed DDI AUC ratios, DDI C_{max} ratios, as well as GMFE values.

5.2 Rifampicin drug-dependent parameters

The drug-dependent parameters of the rifampicin model are summarized in Table S5.2.1 below. The associated system-dependent parameters are listed in Table S7.0.1.

Table S5.2.1: Drug-dependent parameters of the rifampicin PBPK model (adopted from [46])

Parameter	Value	Unit	Source	Literature	Reference	Description
MW	822.94	g/mol	Literature	822.94	[33]	Molecular weight
pKa (acid)	1.70	-	Literature	1.70	[75]	First acid dissociation constant
pKa (base)	7.90	-	Literature	7.90	[75]	Second acid dissociation constant
Solubility (pH 7.5)	2.80	g/l	Literature	2.80	[76]	Solubility
logP	2.50	-	Optimized	1.30, 2.70	[33, 77]	Lipophilicity
fu	17.00	%	Literature	17.00	[78]	Fraction unbound
B/P ratio	0.89	-	Calculated	0.90 °	[79]	Blood/plasma ratio
OATP1B1 K_m	1.50	µmol/l	Literature	1.50	[80]	OATP1B1 Michaelis-Menten constant
OATP1B1 k_{cat}	7.80	1/min	Optimized	-	-	OATP1B1 transport rate constant
AADAC K_m	195.10	µmol/l	Literature	195.10	[81]	AADAC Michaelis-Menten constant
AADAC k_{cat}	9.87	1/min	Optimized	-	-	AADAC catalytic rate constant
Pgp K_m	55.00	µmol/l	Literature	55.00	[82]	Pgp Michaelis-Menten constant
Pgp k_{cat}	0.61	1/min	Optimized	-	-	Pgp transport rate constant
GFR fraction	1.00	-	Assumed	-	-	Fraction of filtered drug in the urine
EHC continuous fraction	1.00	-	Assumed	-	-	Fraction of bile continually released
Induction EC_{50}	0.34	µmol/l	Literature	0.80*0.42 ‡	[78, 83]	Conc. for half-maximal induction
E_{max} OATP1B1	0.38	-	Optimized	-	-	Maximum in vivo induction effect
E_{max} AADAC	0.99	-	Optimized	-	-	Maximum in vivo induction effect
E_{max} Pgp	2.50	-	Literature	2.50	[84]	Maximum in vivo induction effect
E_{max} CYP3A4	9.00	-	Literature	9.00	[78]	Maximum in vivo induction effect
OATP1B1 K_i	0.48	µmol/l	Literature	0.48	[85]	Conc. for half-maximal inhibition
Pgp K_i	169.00	µmol/l	Literature	169.00	[86]	Conc. for half-maximal inhibition
CYP3A4 K_i	18.50	µmol/l	Literature	18.50	[87]	Conc. for half-maximal inhibition
Partition coefficients	Diverse	-	Calculated	R&R	[41, 42]	Cell to plasma partition coefficients
Cellular permeability	2.93E-05	cm/min	Calculated	PK-Sim	[2]	Permeability into the cellular space
Intestinal permeability	1.24E-05	cm/min	Optimized	3.84E-07	Calculated	Transcellular intestinal permeability

° Blood/serum concentration ratio, ‡ in vitro value corrected for binding in the assay, **AADAC**: arylacetamide deacetylase, **conc**: concentration, **CYP3A4**: cytochrome P450 3A4, **EHC**: enterohepatic circulation, **GFR**: glomerular filtration rate, **OATP1B1**: organic anion transporting polypeptide 1B1, **Pgp**: P-glycoprotein, **PK-Sim**: PK-Sim standard calculation method, **R&R**: Rodgers and Rowland calculation method

5.3 Rifampicin-verapamil clinical DDI studies

The clinical studies used to evaluate the rifampicin-verapamil DDI model performance are summarized in Table S5.3.1.

Table S5.3.1: Rifampicin-verapamil DDI study table

**Perpetrator** Rifampicin	**Victim** Verapamil	**Dose gap [h]**	**n**	**Men** [%]	**Age** [years]	**Weight** [kg]	**Height** [cm]	**BMI** [kg/m^2]	**Dataset**	**Reference**
600 mg, po, - , qd	10 mg, iv, 10 min	12	6	67	(24-37)	-	-	-	test	Barbarash et al. 1988 [14]
600 mg, po, - , qd	120 mg, po, -	12	6	67	(24-37)	-	-	-	test	Barbarash et al. 1988 [14]

-: not given, **BMI**: body mass index, **iv**: intravenous, **n**: number of individuals studied, **po**: oral, **qd**: once daily, **test**: test dataset (model evaluation)

5.4 Profiles

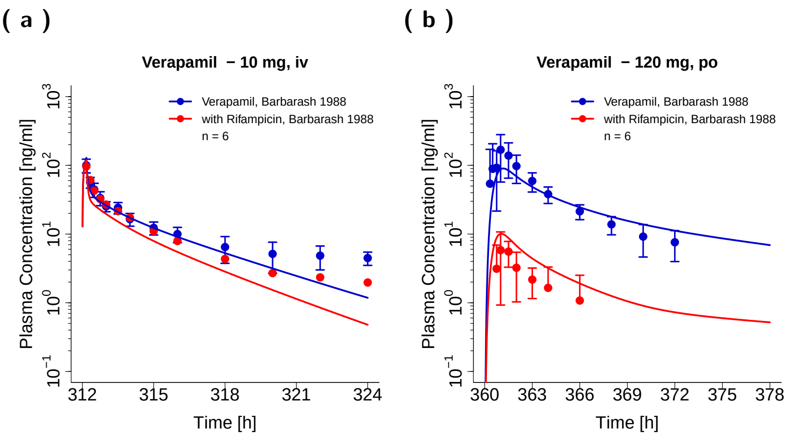

Figure S5.4.1: Verapamil plasma concentration-time profiles (semilogarithmic) before and during rifampicin cotreatment. Observed data are shown as dots, if available ± standard deviation (SD). Simulations are shown as lines.

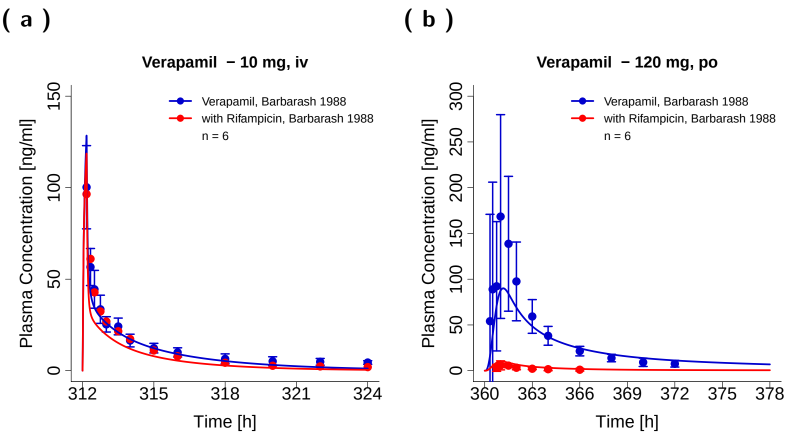

Figure S5.4.2: Verapamil plasma concentration-time profiles (linear) before and during rifampicin cotreatment. Observed data are shown as dots, if available ± standard deviation (SD). Simulations are shown as lines.

5.5 Model evaluation

5.5.1 DDI AUC and C_{max} ratio goodness-of-fit plots

(a) DDI AUC ratios

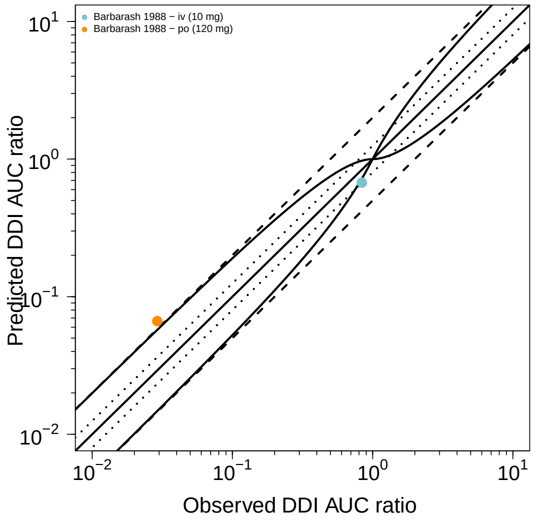

(b) DDI C_{max} ratios

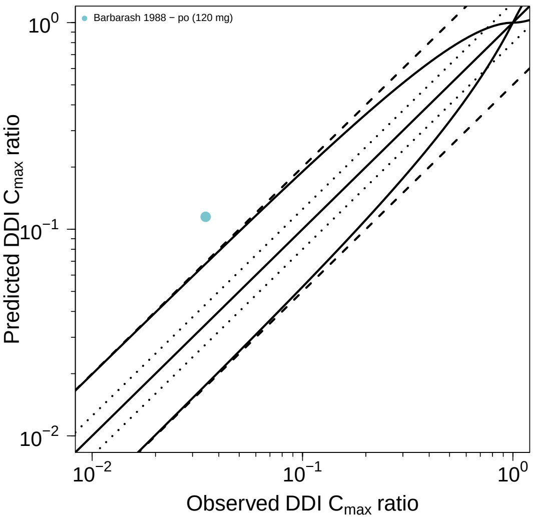

Figure S5.5.1: Predicted versus observed rifampicin-verapamil DDI AUC ratios and DDI C_{max} ratios. Each symbol represents the DDI AUC or C_{max} ratio of a different study profile. The straight solid line (—–) marks the line of identity. The dotted lines (·····) indicate 1.25-fold, the dashed lines (– –) indicate 2-fold deviation. The curved lines show the prediction success limits suggested by Guest et al. [55]. **AUC**: area under the plasma concentration–time curve from the time of administration to the last observed data point, **C_{max}**: maximum plasma concentration

5.5.2 Geometric mean fold error of predicted DDI AUC and C_{max} ratios

Table S5.5.1: Predicted and observed rifampicin-verapamil DDI AUC ratios and DDI C_{max} ratios

**Perpetrator**	**Victim**			**DDI AUC ratio**			**DDI C_{max} ratio**			
Rifampicin	Verapamil	Dose gap [h]	n	Pred	Obs	Pred/Obs	Pred	Obs	Pred/Obs	Reference
600 mg, po, - , qd	10 mg, iv, 10 min	12	6	0.67	0.84	0.80	-	-	-	Barbarash et al. 1988 [14]
600 mg, po, - , qd	120 mg, po, -	12	6	0.07	0.03	2.28	0.11	0.03	3.32	Barbarash et al. 1988 [14]
**Overall GMFE**						**1.68 (1.24–2.28)**			**3.32**	
						**1/2 with GMFE ≤ 2**			**0/1 with GMFE ≤ 2**	

-: not given, **GMFE**: geometric mean fold error, **iv**: intravenous, **n**: number of individuals studied, **obs**: observed, **po**: oral, **pred**: predicted, **qd**: once daily

6 Cimetidine-verapamil drug-drug interaction (DDI)

6.1 DDI modeling

The cimetidine-verapamil DDI was predicted using a previously established whole-body PBPK model of cimetidine [88]. The drug-dependent parameters of this model are reproduced in Table S6.2.1.

The cimetidine-verapamil interaction was modeled as competitive inhibition of CYP3A4 verapamil metabolism by cimetidine. The K_i = 268.0 µmol/l (see Table S6.2.1) for this weak inhibition was obtained from literature [89], determined using human liver microsomes. This value was not corrected for cimetidine binding in vitro, since no experimental values for cimetidine fraction unbound in microsomal incubations could be obtained and the prediction of $fu_{incubation}$ according to Austin et al. [90] resulted in a theoretical $fu_{incubation}$ of 0.97 that did not change the results significantly.

Details on the predicted clinical DDI studies are given in Table S6.3.1. Model predictions of verapamil plasma concentration-time profiles before and during cimetidine co-administration, compared to observed data, are shown in Figures S6.4.1 and S6.4.2. The correlation of predicted to observed DDI AUC ratios and DDI C_{max} ratios is shown in Figure S6.5.1. Table S6.5.1 lists the corresponding predicted and observed DDI AUC ratios, DDI C_{max} ratios, as well as GMFE values.

6.2 Cimetidine drug-dependent parameters

The drug-dependent parameters of the cimetidine model are summarized in Table S6.2.1 below. The associated system-dependent parameters are listed in Table S7.0.1.

Table S6.2.1: Drug-dependent parameters of the cimetidine PBPK model (adopted from [88])

Parameter	Value	Unit	Source	Literature	Reference	Description
MW	252.34	g/mol	Literature	252.34	[33]	Molecular weight
pKa1 (base)	6.93	-	Literature	6.93	[91]	First acid dissociation constant
pKa2 (acid)	13.38	-	Literature	13.38	[33]	Second acid dissociation constant
Solubility (pH 6.8)	24.00	g/l	Literature	24.00	[91]	Solubility
logP	1.66	-	Calculated from B/P ratio	0.48	[91]	Lipophilicity
fu	78.00	%	Literature	78.00	[92]	Fraction unbound
B/P ratio	0.98	-	Literature	0.98 [a]	[93]	Blood/plasma ratio
OCT1 K_m	2600.00	µmol/l	Literature	2600.00	[94]	OCT1 Michaelis-Menten constant
OCT1 k_{cat}	8.66E+04	1/min	Optimized	-	-	OCT1 transport rate constant
CL_{hep}	0.16	1/min	Optimized	-	[93]	Specific hepatic plasma clearance
OAT3 K_m	149.00	µmol/l	Literature	149.00	[95]	OAT3 Michaelis-Menten constant
OAT3 k_{cat}	5.75E+07	1/min	Optimized	-	-	OAT3 transport rate constant
MATE1 K_m	8.00	µmol/l	Literature	8.00	[96]	MATE1 Michaelis-Menten constant
MATE1 k_{cat}	32.37	1/min	Optimized	-	-	MATE1 transport rate constant
GFR fraction	1.00	-	Assumed	-	-	Fraction of filtered drug in the urine
EHC continuous fraction	1.00	-	Assumed	-	-	Fraction of bile continually released
OCT1 K_i	104.00	µmol/l	Literature	104.00	[97]	Conc. for half-maximal inhibition
OCT2 K_i	124.00	µmol/l	Literature	124.00	[97]	Conc. for half-maximal inhibition
MATE1 K_i	3.80	µmol/l	Literature	3.80	[97]	Conc. for half-maximal inhibition
CYP3A4 K_i	268.00	µmol/l	Literature	268.00	[89]	Conc. for half-maximal inhibition
Partition coefficients	Diverse	-	Calculated	R&R	[41, 42]	Cell to plasma partition coefficients
Cellular permeability	5.04E-03	cm/min	Calculated	PK-Sim	[2]	Permeability into the cellular space
Intestinal permeability	8.72E-07	cm/min	Optimized	1.12E-05	Calculated	Transcellular intestinal permeability
Tablet fasted [b]						

[a] in patients, [b] split dose administration with the fraction of dose and lag time for the second gastric emptying optimized in a NONMEM approach, **conc**: concentration, **EHC**: enterohepatic circulation, **GFR**: glomerular filtration rate, **PK-Sim**: PK-Sim standard calculation method, **R&R**: Rodgers and Rowland calculation method

6.3 Cimetidine-verapamil clinical DDI studies

The clinical studies used to evaluate the cimetidine-verapamil DDI model performance are summarized in Table S6.3.1.

Table S6.3.1: Cimetidine-verapamil DDI study table

**Perpetrator** Cimetidine	**Victim** Verapamil	**Dose gap [h]**	**n**	**Men [%]**	**Age [years]**	**Weight [kg]**	**Height [cm]**	**BMI [kg/m^2]**	**Dataset**	**Reference**
200/400 mg, po, - , qid	10 mg, iv, 10 min	0	1	100	21	70	-	-	test	Wing et al. 1985 [15]
300 mg, po, - , qid	10 mg, iv, 10 min	0	1	100	24	67	-	-	test	Abernethy et al. 1985 [13]
300 mg, po, - , qid	10 mg, iv, bolus	0	8	100	27 ± 5 (24-38)	-	-	-	test	Smith et al. 1984 [17]
200/400 mg, po, - , qid	80 mg, po, -	0	1	100	21	70	-	-	test	Wing et al. 1985 [15]
300 mg, po, - , qid	120 mg, po, -	0	1	100	24	67	-	-	test	Abernethy et al. 1985 [13]
300 mg, po, - , qid	120 mg, po, -	0	8	100	27 ± 5 (24-38)	-	-	-	test	Smith et al. 1984 [17]
400 mg, po, - , bid	160 mg, po, sol	0.5	1	100	(25-43)	(66-87)	-	-	test	Mikus et al. 1990 [31]

-: not given, **bid**: twice daily, **BMI**: body mass index, **iv**: intravenous, **n**: number of individuals studied, **po**: oral, **qid**: four times daily, **sol**: solution, **test**: test dataset (model evaluation)

6.4 Profiles

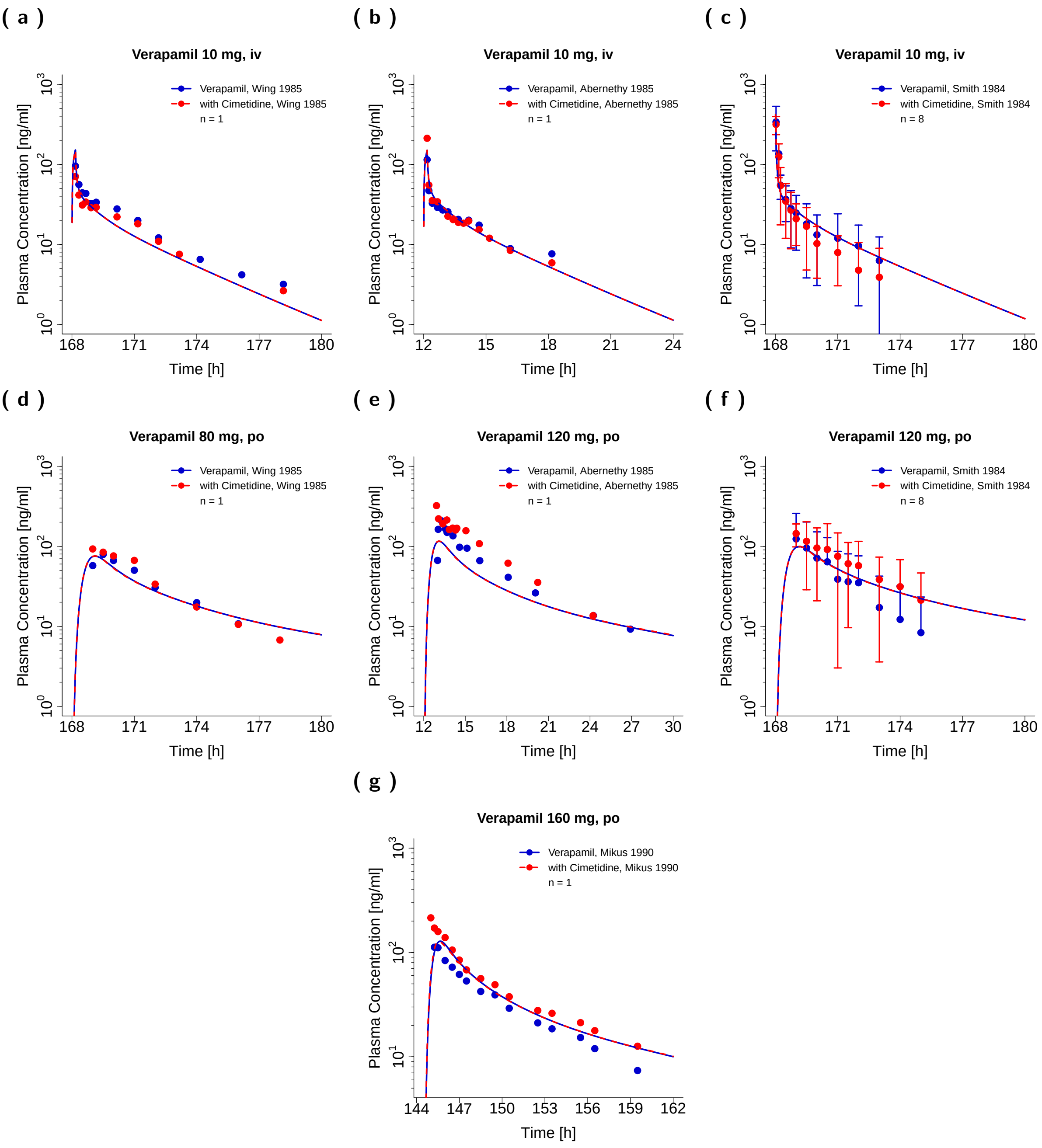

Figure S6.4.1: Verapamil plasma concentration-time profiles (semilogarithmic) before and during cimetidine cotreatment. Observed data are shown as dots, if available ± standard deviation (SD). Simulations are shown as lines.

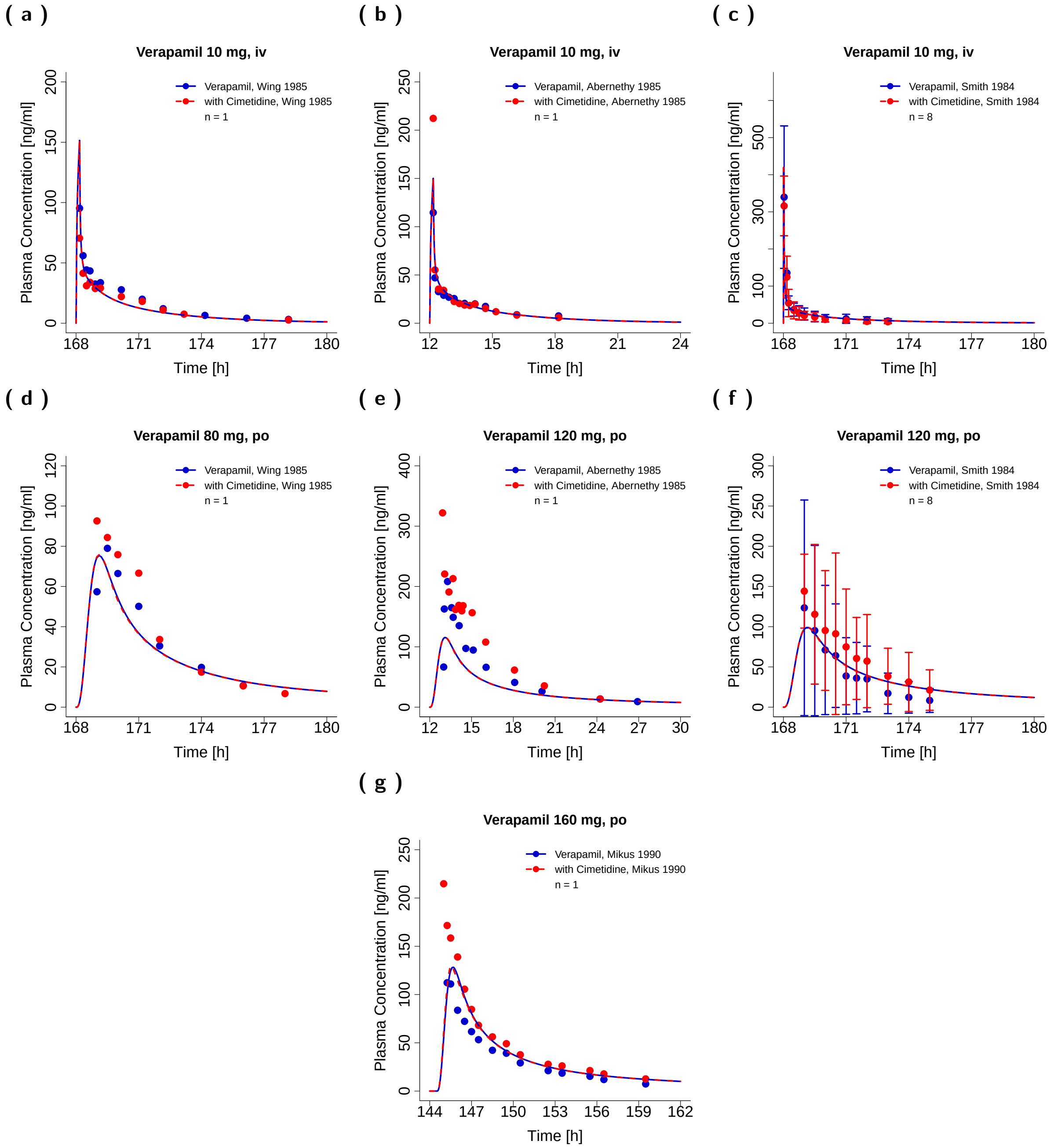

Figure S6.4.2: Verapamil plasma concentration-time profiles (linear) before and during cimetidine cotreatment. Observed data are shown as dots, if available ± standard deviation (SD). Simulations are shown as lines.

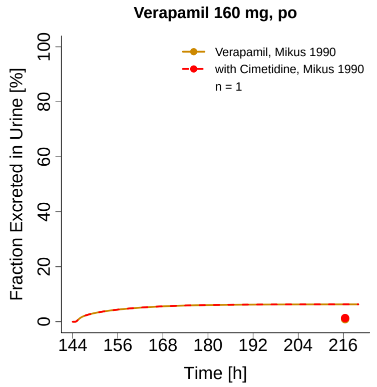

Figure S6.4.3: Verapamil fraction excreted in urine when administered alone and during cimetidine cotreatment. Observed data are shown as dots. Simulations are shown as lines.

6.5 Model evaluation

6.5.1 DDI AUC and C_{max} ratio goodness-of-fit plots

(a) DDI AUC ratios

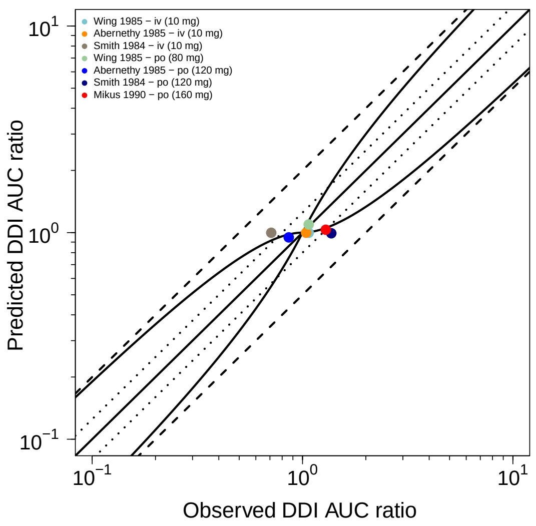

(b) DDI C_{max} ratios

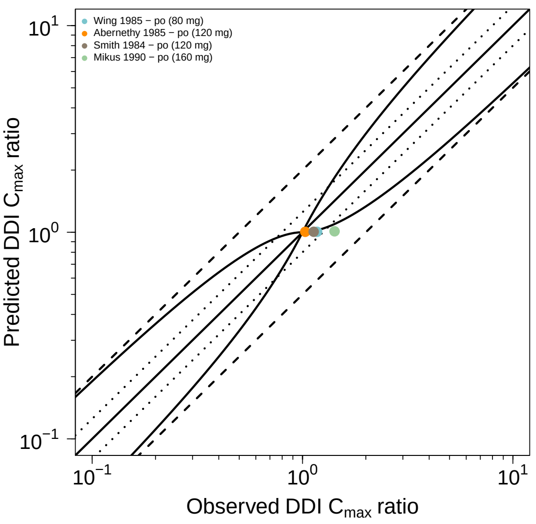

Figure S6.5.1: Predicted versus observed cimetidine-verapamil DDI AUC ratios and DDI C_{max} ratios. Each symbol represents the DDI AUC or C_{max} ratio of a different study profile. The straight solid line (—) marks the line of identity. The dotted lines (·····) indicate 1.25-fold, the dashed lines (- -) indicate 2-fold deviation. The curved lines show the prediction success limits suggested by Guest et al. [55]. **AUC**: area under the plasma concentration–time curve from the time of administration to the last observed data point, $\mathbf{C_{max}}$: maximum plasma concentration

6.5.2 Geometric mean fold error of predicted DDI AUC and C_{max} ratios

Table S6.5.1: Predicted and observed cimetidine-verapamil DDI AUC ratios and DDI C_{max} ratios

**Perpetrator**	**Victim**			**DDI AUC ratio**			**DDI C_{max} ratio**			
Cimetidine	Verapamil	Dose gap [h]	n	Pred	Obs	Pred/Obs	Pred	Obs	Pred/Obs	Reference
200/400 mg, po, - , qid	10 mg, iv, 10 min	0	8	1.00	1.07 [a]	0.93	-	-	-	Wing et al. 1985 [15]
300 mg, po, - , qid	10 mg, iv, 10 min	0	9	1.00	1.04 [a]	0.96	-	-	-	Abernethy et al. 1985 [13]
300 mg, po, - , qid	10 mg, iv, bolus	0	8	1.00	0.71 [a]	1.41	-	-	-	Smith et al. 1984 [17]
200/400 mg, po, - , qid	80 mg, po, -	0	8	1.10	1.07 [a]	1.02	1.01	1.17 [b]	0.86	Wing et al. 1985 [15]
300 mg, po, - , qid	120 mg, po, -	0	9	0.95	0.86 [a]	1.10	1.01	1.03 [a]	0.98	Abernethy et al. 1985 [13]
300 mg, po, - , qid	120 mg, po, -	0	8	0.99	1.37 [a]	0.72	1.01	1.13 [a]	0.89	Smith et al. 1984 [17]
400 mg, po, - , bid	160 mg, po, sol	0.5	6	1.03	1.29 [a]	0.80	1.01	1.42 [a]	0.71	Mikus et al. 1990 [31]
**Overall GMFE**						**1.17 (1.02–1.41)**			**1.17 (1.02–1.41)**	
						**7/7 with GMFE ≤ 2**			**4/4 with GMFE ≤ 2**	

[a] as stated in the reference, [b] calculated from n=1 plasma profile, **-**: not given, **bid**: twice daily, **GMFE**: geometric mean fold error, **iv**: intravenous, **n**: number of individuals studied, **obs**: observed, **po**: oral, **pred**: predicted, **qid**: four times daily, **sol**: solution

7 System-dependent parameters

Details on the expression of metabolizing enzymes, transport proteins and protein binding partners implemented to model the pharmacokinetics of verapamil, midazolam, digoxin, rifampicin and cimetidine are summarized in Table S7.0.1.

Although there are early reports of MATE2-K expression in the kidney [98, 99], a recent study that quantified the protein expression of renal transporters via LC-MS/MS found that the predominant transporters in the kidney are MATE1, OAT1, OAT3 and OCT2 with only negligible amounts of MATE2-K [100].

OCT1 is an uptake transporter that is strongly expressed in the liver [101–103]. Initially allocated to the basolateral membrane of intestinal epithelial cells, recent literature rather supports an apical localization and function of OCT1 in the gut [104, 105].

Table S7.0.1: System-dependent parameters

	Reference concentration					Half-life [h]	
**Enzyme/Transporter**	**Mean** [a]	**GSD** [b]	**Relative expression** [c]	**Localization**	**Direction**	**Liver**	**Intestine**
AADAC	1.00 [d] [106]	1.40 [e]	RT-PCR [109]	Intracellular	-	36	23
ATP1A2	0.48 [46]	1.40 [e]	Array [110]	Membrane	-	36	23
CYP3A4	4.32 [111]	1.18 liver [1]<br>1.46 intestine [1]	RT-PCR [112]	Intracellular	-	36 [113]	23 [114]
MATE1	0.13 [f] [100, 107]	1.53 [100]	Kidney [98, 99]	Apical	Efflux	36	-
OAT3	0.09 [f] [100, 107]	1.53 [100]	RT-PCR [115]	Basolateral	Influx	36	-
OATP1B1	1.00 [d] [106]	1.54 [108]	RT-PCR [115]	Basolateral	Influx	36	-
OCT1	0.16 [g] [108, 116]	1.50 [116]	Array [110], large intestinal mucosa → 0	Basolateral, in enterocytes apical	Influx	36	23
Pgp	1.41 [46]	1.60 [108]	RT-PCR [115], intestinal mucosa → factor 3.57 [46]	Apical	Efflux	36	23

AADAC: arylacetamide deacetylase, **ATP1A2**: ATPase Na^+/K^+ transporting subunit alpha 2, **CYP3A4**: cytochrome P450 3A4, **MATE1**: multidrug and toxin extrusion 1, **OAT3**: organic anion transporter 3, **OATP1B1**: organic anion transporting polypeptide 1B1, **OCT1**: organic cation transporter 1, **Pgp**: P-glycoprotein

[a] µmol protein/l in the tissue of highest expression

[b] Geometric standard deviation of the reference concentration

[c] In the different organs (PK-Sim® expression database profile)

[d] If no information was available, the mean reference concentration was set to 1.0 µmol/l and the catalytic rate constant (k_{cat}) was optimized [106]

[e] If no information was available, a moderate variability of 35 % CV was assumed (= 1.40 GSD)

[f] Calculated from transporter per mg membrane protein × 26.2 mg human kidney microsomal protein per g kidney [107]

[g] Calculated from transporter per mg membrane protein × 37.0 mg membrane protein per g liver [108]

References

[1] Open Systems Pharmacology Suite Community (2018) PK-Sim® Ontogeny Database Documentation, Version 7.3. https://github.com/Open-Systems-Pharmacology/OSPSuite.Documentation/blob/master/PK-SimOntogenyDatabaseVersion7.3.pdf, accessed: 2020-02-25

[2] Open Systems Pharmacology Suite Community (2018) Open Systems Pharmacology Suite Manual, Version 7.4. https://github.com/Open-Systems-Pharmacology/OSPSuite.Documentation/blob/master/OpenSystemsPharmacologySuite.pdf, accessed: 2020-02-25

[3] Schomerus M, Spiegelhalder B, Stieren B, Eichelbaum M (1976) Physiological disposition of verapamil in man. Cardiovascular research 10(5):605–12

[4] Eichelbaum M, Ende M, Remberg G, Schomerus M, Dengler HJ (1979) The metabolism of DL-[14C]verapamil in man. Drug metabolism and disposition: the biological fate of chemicals 7(3):145–8

[5] US Food and Drug Administration (2019) Drug development and drug interactions: Table of substrates, inhibitors and inducers. https://www.fda.gov/drugs/drug-interactions-labeling/drug-development-and-drug-interactions-table-substrates-inhibitors-and-inducers, accessed: 2020-02-25

[6] Sandström R, Karlsson A, Knutson L, Lennernäs H (1998) Jejunal absorption and metabolism of R/S-verapamil in humans. Pharmaceutical research 15(6):856–62

[7] Luurtsema G, Molthoff CFM, Windhorst AD, Smit JW, Keizer H, Boellaard R, Lammertsma AA, Franssen EJF (2003) (R)- and (S)-[11C]verapamil as PET-tracers for measuring P-glycoprotein function: in vitro and in vivo evaluation. Nuclear medicine and biology 30(7):747–51

[8] Engman H, Tannergren C, Artursson P, Lennernäs H (2003) Enantioselective transport and CYP3A4-mediated metabolism of R/S-verapamil in Caco-2 cell monolayers. European journal of pharmaceutical sciences : official journal of the European Federation for Pharmaceutical Sciences 19(1):57–65

[9] Eichelbaum M, Mikus G, Vogelgesang B (1984) Pharmacokinetics of (+)-, (-)- and (+/-)-verapamil after intravenous administration. British journal of clinical pharmacology 17(4):453–8

[10] Mooy J, Schols M, v Baak M, v Hooff M, Muytjens A, Rahn KH (1985) Pharmacokinetics of verapamil in patients with renal failure. European journal of clinical pharmacology 28(4):405–10

[11] Streit M, Göggelmann C, Dehnert C, Burhenne J, Riedel KD, Menold E, Mikus G, Bärtsch P, Haefeli WE (2005) Cytochrome P450 enzyme-mediated drug metabolism at exposure to acute hypoxia (corresponding to an altitude of 4,500 m). European journal of clinical pharmacology 61(1):39–46

[12] Johnston A, Burgess CD, Hamer J (1981) Systemic availability of oral verapamil and effect on PR interval in man. British journal of clinical pharmacology 12(3):397–400

[13] Abernethy DR, Schwartz JB, Todd EL (1985) Lack of interaction between verapamil and cimetidine. Clinical pharmacology and therapeutics 38(3):342–9

[14] Barbarash RA, Bauman JL, Fischer JH, Kondos GT, Batenhorst RL (1988) Near-total reduction in verapamil bioavailability by rifampin. Electrocardiographic correlates. Chest 94(5):954–9

[15] Wing LM, Miners JO, Lillywhite KJ (1985) Verapamil disposition–effects of sulphinpyrazone and cimetidine. British journal of clinical pharmacology 19(3):385–91

[16] McAllister RG, Kirsten EB (1982) The pharmacology of verapamil. IV. Kinetic and dynamic effects after single intravenous and oral doses. Clinical pharmacology and therapeutics 31(4):418–26

[17] Smith MS, Benyunes MC, Bjornsson TD, Shand DG, Pritchett EL (1984) Influence of cimetidine on verapamil kinetics and dynamics. Clinical pharmacology and therapeutics 36(4):551–4

[18] Freedman SB, Richmond DR, Ashley JJ, Kelly DT (1981) Verapamil kinetics in normal subjects and patients with coronary artery spasm. Clinical pharmacology and therapeutics 30(5):644–52

[19] Abernethy DR, Wainer IW, Longstreth JA, Andrawis NS (1993) Stereoselective verapamil disposition and dynamics in aging during racemic verapamil administration. The Journal of pharmacology and experimental therapeutics 266(2):904–11

[20] Vogelgesang B, Echizen H, Schmidt E, Eichelbaum M (1984) Stereoselective first-pass metabolism of highly cleared drugs: studies of the bioavailability of L- and D-verapamil examined with a stable isotope technique. British journal of clinical pharmacology 18(5):733–40

[21] Maeda K, Takano J, Ikeda Y, Fujita T, Oyama Y, Nozawa K, Kumagai Y, Sugiyama Y (2011) Nonlinear pharmacokinetics of oral quinidine and verapamil in healthy subjects: a clinical microdosing study. Clinical pharmacology and therapeutics 90(2):263–70

[22] Blume H, Mutschler E (1989) Bioäquivalenz: Qualitätsbewertung wirkstoffgleicher Fertigarzneimittel: Anleitung, Methoden, Materialien. Govi-Verlag

[23] John DN, Fort S, Lewis MJ, Luscombe DK (1992) Pharmacokinetics and pharmacodynamics of verapamil following sublingual and oral administration to healthy volunteers. British journal of clinical pharmacology 33(6):623–7

[24] Sawicki W, Janicki S (2002) Pharmacokinetics of verapamil and its metabolite norverapamil from a buccal drug formulation. International journal of pharmaceutics 238(1-2):181–9

[25] Choi DH, Shin WG, Choi JS (2008) Drug interaction between oral atorvastatin and verapamil in healthy subjects: effects of atorvastatin on the pharmacokinetics of verapamil and norverapamil. European journal of clinical pharmacology 64(5):445–9

[26] ratiopharm GmbH (2016) Fachinformation Verapamil-ratiopharm® N 40 mg / 80 mg Filmtabletten

[27] Johnson BF, Cheng SL, Venitz J (2001) Transient kinetic and dynamic interactions between verapamil and dofetilide, a class III antiarrhythmic. Journal of clinical pharmacology 41(11):1248–56

[28] Boehringer Ingelheim Pharma GmbH & Co KG (2018) The effect of potent inhibitors of drug transporters (verapamil, rifampin, cimetidine, probenecid) on pharmacokinetics of a transporter probe drug cocktail consisting of digoxin, furosemide, metformin and rosuvastatin. EudraCT 2017-001549-29. https://clinicaltrials.gov/ct2/show/record/NCT03307252, accessed: 2020-02-25

[29] Härtter S, Sennewald R, Nehmiz G, Reilly P (2012) Oral bioavailability of dabigatran etexilate (Pradaxa(®)) after co-medication with verapamil in healthy subjects. British journal of clinical pharmacology 75(4):1053–62

[30] Hla KK, Henry JA, Latham AN (1987) Pharmacokinetics and pharmacodynamics of two formulations of verapamil. British journal of clinical pharmacology 24(5):661–4

[31] Mikus G, Eichelbaum M, Fischer C, Gumulka S, Klotz U, Kroemer HK (1990) Interaction of verapamil and cimetidine: stereochemical aspects of drug metabolism, drug disposition and drug action. The Journal of pharmacology and experimental therapeutics 253(3):1042–8

[32] van Haarst AD, Dijkmans AC, Weimann HJ, Kemme MJB, Bosch JJ, Schoemaker RC, Cohen AF, Burggraaf J (2009) Clinically important interaction between tedisamil and verapamil. Journal of clinical pharmacology 49(5):560–7

[33] Wishart DS, Knox C, Guo AC, Shrivastava S, Hassanali M, Stothard P, Chang Z, Woolsey J (2006) DrugBank: a comprehensive resource for in silico drug discovery and exploration. Nucleic Acids Research 34(Supplement 1):D668–D672

[34] Hasegawa J, Fujita T, Hayashi Y, Iwamoto K, Watanabe J (1984) pKa determination of verapamil by liquid-liquid partition. Journal of pharmaceutical sciences 73(4):442–5

[35] Vogelpoel H, Welink J, Amidon GL, Junginger HE, Midha KK, Möller H, Olling M, Shah VP, Barends DM (2004) Biowaiver monographs for immediate release solid oral dosage forms based on biopharmaceutics classification system (BCS) literature data: verapamil hydrochloride, propranolol hydrochloride, and atenolol. Journal of pharmaceutical sciences 93(8):1945–56

[36] Hansch C, Leo A, Hoekman D (1995) Exploring QSAR: Hydrophobic, electronic, and steric constants. American Chemical Society, Washington, DC

[37] Sanaee F, Clements JD, Waugh AWG, Fedorak RN, Lewanczuk R, Jamali F (2011) Drug-disease interaction: Crohn's disease elevates verapamil plasma concentrations but reduces response to the drug proportional to disease activity. British journal of clinical pharmacology 72(5):787–97

[38] Wang J, Xia S, Xue W, Wang D, Sai Y, Liu L, Liu X (2013) A semi-physiologically-based pharmacokinetic model characterizing mechanism-based auto-inhibition to predict stereoselective pharmacokinetics of verapamil and its metabolite norverapamil in human. European journal of pharmaceutical sciences : official journal of the European Federation for Pharmaceutical Sciences 50(3-4):290–302

[39] Shirasaka Y, Sakane T, Yamashita S (2008) Effect of P-glycoprotein expression levels on the concentration-dependent permeability of drugs to the cell membrane. Journal of pharmaceutical sciences 97(1):553–65

[40] Döppenschmitt S, Langguth P, Regårdh CG, Andersson TB, Hilgendorf C, Spahn-Langguth H (1999) Characterization of binding properties to human P-glycoprotein: development of a [3H]verapamil radioligand-binding assay. The Journal of pharmacology and experimental therapeutics 288(1):348–57

[41] Rodgers T, Leahy D, Rowland M (2005) Physiologically based pharmacokinetic modeling 1: Predicting the tissue distribution of moderate-to-strong bases. Journal of Pharmaceutical Sciences 94(6):1259–1276

[42] Rodgers T, Rowland M (2006) Physiologically based pharmacokinetic modelling 2: Predicting the tissue distribution of acids, very weak bases, neutrals and zwitterions. Journal of Pharmaceutical Sciences 95(6):1238–1257

[43] Sigma-Aldrich Inc (2013) A Case Study in SPE Method Development - Understanding the Dual Interaction Properties of Discovery DSC-SCX SPE Using Verapamil (and Metabolite) from Serum as a Test Example. https://www.sigmaaldrich.com/technical-documents/articles/reporter-eu/a-case-study-in-spe.html, accessed: 2020-02-25

[44] Tracy TS, Korzekwa KR, Gonzalez FJ, Wainer IW (1999) Cytochrome P450 isoforms involved in metabolism of the enantiomers of verapamil and norverapamil. British journal of clinical pharmacology 47(5):545–52

[45] Pauli-Magnus C, von Richter O, Burk O, Ziegler A, Mettang T, Eichelbaum M, Fromm MF (2000) Characterization of the major metabolites of verapamil as substrates and inhibitors of P-glycoprotein. The Journal of pharmacology and experimental therapeutics 293(2):376–82

[46] Hanke N, Frechen S, Moj D, Britz H, Eissing T, Wendl T, Lehr T (2018) PBPK models for CYP3A4 and P-gp DDI prediction: A modeling network of rifampicin, itraconazole, clarithromycin, midazolam, alfentanil, and digoxin. CPT: Pharmacometrics & Systems Pharmacology 7(10):647–659

[47] Walser A, Benjamin LE, Flynn T, Mason C, Schwartz R, Fryer RI (1978) Quinazolines and 1,4-benzodiazepines. 84. Synthesis and reactions of imidazo[1,5-a][1,4]benzodiazepines. The Journal of Organic Chemistry 43(5):936–944

[48] Heikkinen AT, Baneyx G, Caruso A, Parrott N (2012) Application of PBPK modeling to predict human intestinal metabolism of CYP3A substrates – an evaluation and case study using GastroPlus® . European Journal of Pharmaceutical Sciences 47(2):375–386

[49] Vossen M, Sevestre M, Niederalt C, Jang IJ, Willmann S, Edginton AN (2007) Dynamically simulating the interaction of midazolam and the CYP3A4 inhibitor itraconazole using individual coupled whole-body physiologically-based pharmacokinetic (WB-PBPK) models. Theoretical Biology and Medical Modelling 4(1):13

[50] Lemaitre F, Hasni N, Leprince P, Corvol E, Belhabib G, Fillâtre P, Luyt CE, Leven C, Farinotti R, Fernandez C, Combes A (2015) Propofol, midazolam, vancomycin and cyclosporine therapeutic drug monitoring in extracorporeal membrane oxygenation circuits primed with whole human blood. Critical Care 19(1):40

[51] Björkman S, Wada D, Berling B, Benoni G (2001) Prediction of the disposition of midazolam in surgical patients by a physiologically based pharmacokinetic model. Journal of Pharmaceutical Sciences 90(9):1226–1241

[52] Patki KC, von Moltke LL, Greenblatt DJ (2003) In vitro metabolism of midazolam, triazolam, nifedipine, and testosterone by human liver microsomes and recombinant cytochromes P450: role of CYP3A4 and CYP3A5. Drug Metabolism and Disposition 31(7):938–944

[53] Wang Y, Jin Y, Hilligoss JK, Ho H, Hamman MA, Hu Z, Gorski JC, Hall SD (2005) Effect of CYP3A5 genotype on the extent of CYP3A inhibition by verapamil. Clinical Pharmacology & Therapeutics 77(2):P3–P3

[54] Backman JT, Olkkola KT, Aranko K, Himberg JJ, Neuvonen PJ (1994) Dose of midazolam should be reduced during diltiazem and verapamil treatments. British journal of clinical pharmacology 37(3):221–5

[55] Guest EJ, Aarons L, Houston JB, Rostami-Hodjegan A, Galetin A (2011) Critique of the two-fold measure of prediction success for ratios: Application for the assessment of drug-drug interactions. Drug Metabolism and Disposition 39(2):170–173

[56] Ito S, Woodland C, Harper PA, Koren G (1993) The mechanism of the verapamil-digoxin interaction in renal tubular cells (LLC-PK1). Life sciences 53(24):PL399–403

[57] Woodland C, Koren G, Wainer IW, Batist G, Ito S (2003) Verapamil metabolites: potential P-glycoprotein-mediated multidrug resistance reversal agents. Canadian journal of physiology and pharmacology 81(8):800–5

[58] Pedersen KE, Dorph-Pedersen A, Hvidt S, Klitgaard NA, Pedersen KK (1982) The long-term effect of verapamil on plasma digoxin concentration and renal digoxin clearance in healthy subjects. European journal of clinical pharmacology 22(2):123–7

[59] Liu Y, Hunt CA (2005) Studies of intestinal drug transport using an in silico epithelio-mimetic device. Bio Systems 82(2):154–67

[60] Yalkowsky SH, Dannenfelser RM (1992) Aquasol database of aqueous solubility

[61] Hinderling PH (1984) Kinetics of partitioning and binding of digoxin and its analogues in the subcompartments of blood. Journal of pharmaceutical sciences 73(8):1042–53

[62] Alsenz J, Meister E, Haenel E (2007) Development of a partially automated solubility screening (PASS) assay for early drug development. Journal of pharmaceutical sciences 96(7):1748–62

[63] Atkinson HC, Begg EJ (1988) Relationship between human milk lipid-ultrafiltrate and octanol-water partition coefficients. Journal of pharmaceutical sciences 77(9):796–8

[64] Neuhoff S, Yeo KR, Barter Z, Jamei M, Turner DB, Rostami-Hodjegan A (2013) Application of permeability-limited physiologically-based pharmacokinetic models: part I-digoxin pharmacokinetics incorporating P-glycoprotein-mediated efflux. Journal of pharmaceutical sciences 102(9):3145–60

[65] Katz A, Lifshitz Y, Bab-Dinitz E, Kapri-Pardes E, Goldshleger R, Tal DM, Karlish SJD (2010) Selectivity of digitalis glycosides for isoforms of human Na,K-ATPase. The Journal of biological chemistry 285(25):19582–92

[66] Troutman MD, Thakker DR (2003) Efflux ratio cannot assess P-glycoprotein-mediated attenuation of absorptive transport: asymmetric effect of P-glycoprotein on absorptive and secretory transport across Caco-2 cell monolayers. Pharmaceutical research 20(8):1200–9

[67] Johnson BF, Wilson J, Marwaha R, Hoch K, Johnson J (1987) The comparative effects of verapamil and a new dihydropyridine calcium channel blocker on digoxin pharmacokinetics. Clinical pharmacology and therapeutics 42(1):66–71

[68] Pedersen KE, Thayssen P, Klitgaard NA, Christiansen BD, Nielsen-Kudsk F (1983) Influence of verapamil on the inotropism and pharmacokinetics of digoxin. European journal of clinical pharmacology 25(2):199–206

[69] Belz GG, Doering W, Munkes R, Matthews J (1983) Interaction between digoxin and calcium antagonists and antiarrhythmic drugs. Clinical pharmacology and therapeutics 33(4):410–7

[70] Doering W (1983) Effect of coadministration of verapamil and quinidine on serum digoxin concentration. European journal of clinical pharmacology 25(4):517–21

[71] Klein HO, Lang R, Weiss E, Di Segni E, Libhaber C, Guerrero J, Kaplinsky E (1982) The influence of verapamil on serum digoxin concentration. Circulation 65(5):998–1003

[72] Rodin SM, Johnson BF, Wilson J, Ritchie P, Johnson J (1988) Comparative effects of verapamil and isradipine on steady-state digoxin kinetics. Clinical pharmacology and therapeutics 43(6):668–72

[73] Schwartz JB, Keefe D, Kates RE, Kirsten E, Harrison DC (1982) Acute and chronic pharmacodynamic interaction of verapamil and digoxin in atrial fibrillation. Circulation 65(6):1163–70

[74] Türk D, Hanke N, Wolf S, Frechen S, Eissing T, Wendl T, Schwab M, Lehr T (2019) Physiologically Based Pharmacokinetic Models for Prediction of Complex CYP2C8 and OATP1B1 (SLCO1B1) Drug-Drug-Gene Interactions: A Modeling Network of Gemfibrozil, Repaglinide, Pioglitazone, Rifampicin, Clarithromycin and Itraconazole. Clinical pharmacokinetics 58(12):1595–1607

[75] Merck Research Laboratories (2006) The Merck Index 14th edition: Rifampin. Merck & Co., Inc., Whitehouse Station, NJ, USA

[76] Boman G, Ringberger VA (1974) Binding of rifampicin by human plasma proteins. European journal of clinical pharmacology 7(5):369–73

[77] Baneyx G, Parrott N, Meille C, Iliadis A, Lavé T (2014) Physiologically based pharmacokinetic modeling of CYP3A4 induction by rifampicin in human: influence of time between substrate and inducer administration. European journal of pharmaceutical sciences : official journal of the European Federation for Pharmaceutical Sciences 56:1–15

[78] Templeton IE, Houston JB, Galetin A (2011) Predictive utility of in vitro rifampin induction data generated in fresh and cryopreserved human hepatocytes, Fa2N-4, and HepaRG cells. Drug metabolism and disposition: the biological fate of chemicals 39(10):1921–9

[79] Loos U, Musch E, Jensen JC, Mikus G, Schwabe HK, Eichelbaum M (1985) Pharmacokinetics of oral and intravenous rifampicin during chronic administration. Klinische Wochenschrift 63(23):1205–11

[80] Tirona RG, Leake BF, Wolkoff AW, Kim RB (2003) Human organic anion transporting polypeptide-C (SLC21A6) is a major determinant of rifampin-mediated pregnane X receptor activation. The Journal of pharmacology and experimental therapeutics 304(1):223–8

[81] Nakajima A, Fukami T, Kobayashi Y, Watanabe A, Nakajima M, Yokoi T (2011) Human arylacetamide deacetylase is responsible for deacetylation of rifamycins: rifampicin, rifabutin, and rifapentine. Biochemical pharmacology 82(11):1747–56

[82] Collett A, Tanianis-Hughes J, Hallifax D, Warhurst G (2004) Predicting P-glycoprotein effects on oral absorption: correlation of transport in Caco-2 with drug pharmacokinetics in wild-type and mdr1a(-/-) mice in vivo. Pharmaceutical research 21(5):819–26

[83] Shou M, Hayashi M, Pan Y, Xu Y, Morrissey K, Xu L, Skiles GL (2008) Modeling, prediction, and in vitro in vivo correlation of CYP3A4 induction. Drug metabolism and disposition: the biological fate of chemicals 36(11):2355–70

[84] Greiner B, Eichelbaum M, Fritz P, Kreichgauer HP, von Richter O, Zundler J, Kroemer HK (1999) The role of intestinal P-glycoprotein in the interaction of digoxin and rifampin. The Journal of clinical investigation 104(2):147–53

[85] Hirano M, Maeda K, Shitara Y, Sugiyama Y (2006) Drug-drug interaction between pitavastatin and various drugs via OATP1B1. Drug metabolism and disposition: the biological fate of chemicals 34(7):1229–36

[86] Reitman ML, Chu X, Cai X, Yabut J, Venkatasubramanian R, Zajic S, Stone JA, Ding Y, Witter R, Gibson C, Roupe K, Evers R, Wagner JA, Stoch A (2011) Rifampin's acute inhibitory and chronic inductive drug interactions: experimental and model-based approaches to drug-drug interaction trial design. Clinical pharmacology and therapeutics 89(2):234–42

[87] Kajosaari LI, Laitila J, Neuvonen PJ, Backman JT (2005) Metabolism of repaglinide by CYP2C8 and CYP3A4 in vitro: effect of fibrates and rifampicin. Basic & clinical pharmacology & toxicology 97(4):249–56

[88] Hanke N, Türk D, Selzer D, Ishiguro N, Ebner T, Wiebe S, Müller F, Stopfer P, Nock V, Lehr T (2020) A Comprehensive Whole-Body Physiologically Based Pharmacokinetic Drug-Drug-Gene Interaction Model of Metformin and Cimetidine in Healthy Adults and Renally Impaired Individuals. Clinical Pharmacokinetics p accepted for publication

[89] Wrighton SA, Ring BJ (1994) Inhibition of human CYP3A catalyzed 1'-hydroxy midazolam formation by ketoconazole, nifedipine, erythromycin, cimetidine, and nizatidine. Pharmaceutical Research 11(6):921–924

[90] Austin RP, Barton P, Cockroft SL, Wenlock MC, Riley RJ (2002) The influence of nonspecific microsomal binding on apparent intrinsic clearance, and its prediction from physicochemical properties. Drug Metabolism and Disposition 30(12):1497–1503

[91] Avdeef A, Berger CM (2001) pH-metric solubility. 3. Dissolution titration template method for solubility determination. European Journal of Pharmaceutical Sciences 14(4):281–291

[92] Taylor DC, Cresswell PR, Bartlett DC (1978) The metabolism and elimination of cimetidine, a histamine H2-receptor antagonist, in the rat, dog, and man. Drug Metabolism and Disposition 6(1):21–30

[93] Somogyi A, Gugler R (1983) Clinical Pharmacokinetics of Cimetidine. Clinical Pharmacokinetics 8(6):463–495

[94] Umehara KI, Iwatsubo T, Noguchi K, Kamimura H (2007) Functional involvement of organic cation transporter1 (OCT1/Oct1) in the hepatic uptake of organic cations in humans and rats. Xenobiotica 37(8):818–831

[95] Tahara H, Kusuhara H, Endou H, Koepsell H, Imaoka T, Fuse E, Sugiyama Y (2005) A Species Difference in the Transport Activities of H2 Receptor Antagonists by Rat and Human Renal Organic Anion and Cation Transporters. Journal of Pharmacology and Experimental Therapeutics 315(1):337–345

[96] Ohta Ky, Inoue K, Yasujima T, Ishimaru M, Yuasa H (2010) Functional Characteristics of Two Human MATE Transporters: Kinetics of Cimetidine Transport and Profiles of Inhibition by Various Compounds. Journal of Pharmacy & Pharmaceutical Sciences 12(3):388–396

[97] Ito S, Kusuhara H, Yokochi M, Toyoshima J, Inoue K, Yuasa H, Sugiyama Y (2012) Competitive Inhibition of the Luminal Efflux by Multidrug and Toxin Extrusions, but Not Basolateral Uptake by Organic Cation Transporter 2, Is the Likely Mechanism Underlying the Pharmacokinetic Drug-Drug Interactions Caused by Cimetidine in the Kidney. Journal of Pharmacology and Experimental Therapeutics 340(2):393–403

[98] Otsuka M, Matsumoto T, Morimoto R, Arioka S, Omote H, Moriyama Y (2005) A human transporter protein that mediates the final excretion step for toxic organic cations. Proceedings of the National Academy of Sciences of the United States of America 102(50):17923–17928

[99] Masuda S, Terada T, Yonezawa A, Tanihara Y, Kishimoto K, Katsura T, Ogawa O, Inui KI (2006) Identification and functional characterization of a new human kidney-specific H+/organic cation antiporter, kidney-specific multidrug and toxin extrusion 2. Journal of the American Society of Nephrology 17(8):2127–2135

[100] Prasad B, Johnson K, Billington S, Lee C, Chung GW, Brown CDA, Kelly EJ, Himmelfarb J, Unadkat JD (2016) Abundance of drug transporters in the human kidney cortex as quantified by quantitative targeted proteomics. Drug Metabolism and Disposition 44(12):1920–1924

[101] Gorboulev V, Ulzheimer JC, Akhoundova A, Ulzheimer-Teuber I, Karbach U, Quester S, Baumann C, Lang F, Busch AE, Koepsell H (1997) Cloning and Characterization of Two Human Polyspecific Organic Cation Transporters. DNA and Cell Biology 16(7):871–881

[102] Nies AT, Koepsell H, Winter S, Burk O, Klein K, Kerb R, Zanger UM, Keppler D, Schwab M, Schaeffeler E (2009) Expression of organic cation transporters OCT1 (SLC22A1) and OCT3 (SLC22A3) is affected by genetic factors and cholestasis in human liver. Hepatology 50(4):1227–1240

[103] Prasad B, Gaedigk A, Vrana M, Gaedigk R, Leeder JS, Salphati L, Chu X, Xiao G, Hop C, Evers R, Gan L, Unadkat JD (2016) Ontogeny of hepatic drug transporters as quantified by LC-MS/MS proteomics. Clinical Pharmacology & Therapeutics 100(4):362–370

[104] Han TK, Everett RS, Proctor WR, Ng CM, Costales CL, Brouwer KLR, Thakker DR (2013) Organic Cation Transporter 1 (OCT1/mOct1) Is Localized in the Apical Membrane of Caco-2 Cell Monolayers and Enterocytes. Molecular Pharmacology 84(2):182–189

[105] Han TK, Proctor WR, Costales CL, Cai H, Everett RS, Thakker DR (2015) Four cation-selective transporters contribute to apical uptake and accumulation of metformin in caco-2 cell monolayers. Journal of Pharmacology and Experimental Therapeutics 352(3):519–528

[106] Meyer M, Schneckener S, Ludewig B, Kuepfer L, Lippert J (2012) Using Expression Data for Quantification of Active Processes in Physiologically Based Pharmacokinetic Modeling. Drug Metabolism and Disposition 40(5):892–901

[107] Scotcher D, Billington S, Brown J, Jones CR, Brown CDA, Rostami-Hodjegan A, Galetin A (2017) Microsomal and cytosolic scaling factors in dog and human kidney cortex and application for in vitro-in vivo extrapolation of renal metabolic clearance. Drug Metabolism and Disposition 45(5):556–568

[108] Prasad B, Evers R, Gupta A, Hop CECA, Salphati L, Shukla S, Ambudkar SV, Unadkat JD (2014) Interindividual variability in hepatic organic anion-transporting polypeptides and P-Glycoprotein (ABCB1) protein expression: Quantification by liquid chromatography tandem mass spectroscopy and influence of genotype, age, and sex. Drug Metabolism and Disposition 42(1):78–88

[109] Nishimura M, Naito S (2006) Tissue-specific mRNA expression profiles of human phase I metabolizing enzymes except for cytochrome P450 and phase II metabolizing enzymes. Drug metabolism and pharmacokinetics 21(5):357–74

[110] Kolesnikov N, Hastings E, Keays M, Melnichuk O, Tang YA, Williams E, Dylag M, Kurbatova N, Brandizi M, Burdett T, Megy K, Pilicheva E, Rustici G, Tikhonov A, Parkinson H, Petryszak R, Sarkans U, Brazma A (2015) ArrayExpress update—simplifying data submissions. Nucleic Acids Research 43(D1):D1113–D1116

[111] Rodrigues AD (1999) Integrated cytochrome P450 reaction phenotyping: attempting to bridge the gap between cDNA-expressed cytochromes P450 and native human liver microsomes. Biochemical pharmacology 57(5):465–480

[112] Nishimura M, Yaguti H, Yoshitsugu H, Naito S, Satoh T (2003) Tissue distribution of mRNA expression of human cytochrome P450 isoforms assessed by high-sensitivity real-time reverse transcription PCR. Yakugaku Zasshi 123(5):369–375

[113] Yeo KR, Walsky RL, Jamei M, Rostami-Hodjegan A, Tucker GT (2011) Prediction of time-dependent CYP3A4 drug–drug interactions by physiologically based pharmacokinetic modelling: Impact of inactivation parameters and enzyme turnover. European Journal of Pharmaceutical Sciences 43(3):160–173

[114] Greenblatt DJ, von Moltke LL, Harmatz JS, Chen G, Weemhoff JL, Jen C, Kelley CJ, LeDuc BW, Zinny MA (2003) Time course of recovery of cytochrome p450 3a function after single doses of grapefruit juice. Clinical Pharmacology & Therapeutics 74(2):121–129

[115] Nishimura M, Naito S (2005) Tissue-specific mRNA expression profiles of human ATP-binding cassette and solute carrier transporter superfamilies. Drug Metabolism and Pharmacokinetics 20(6):452–477

[116] Wang L, Prasad B, Salphati L, Chu X, Gupta A, Hop CECA, Evers R, Unadkat JD (2015) Interspecies Variability in Expression of Hepatobiliary Transporters across Human, Dog, Monkey, and Rat as Determined by Quantitative Proteomics. Drug Metabolism and Disposition 43(3):367–374
